# Making the longest sugars: a chemical synthesis of heparin-related [4]_*n*_ oligosaccharides from 16-mer to 40-mer†
†Electronic supplementary information (ESI) available. See DOI: 10.1039/c5sc02091c


**DOI:** 10.1039/c5sc02091c

**Published:** 2015-07-24

**Authors:** Steen U. Hansen, Gavin J. Miller, Matthew J. Cliff, Gordon C. Jayson, John M. Gardiner

**Affiliations:** a Manchester Institute of Biotechnology and School of Chemistry , University of Manchester , 131 Princess Street , M1 7DN , UK . Email: gardiner@manchester.ac.uk ; Tel: +44 (0)161 306 4530; b Manchester Institute of Biotechnology and Faculty of Life Sciences , The University of Manchester , 131 Princess Street , Manchester M1 7DN , UK; c Institute or Cancer Studies , University of Manchester , Manchester , UK

## Abstract

The chemical synthesis of long oligosaccharides remains a major challenge. In particular, the synthesis of glycosaminoglycan (GAG) oligosaccharides belonging to the heparin and heparan sulfate (H/HS) family has been a high profile target, particularly with respect to the longer heparanome. Herein we describe a synthesis of the longest heparin-related oligosaccharide to date and concurrently provide an entry to the longest synthetic oligosaccharides of any type yet reported. Specifically, the iterative construction of a series of [4]_*n*_-mer heparin-backbone oligosaccharides ranging from 16-mer through to the 40-mer in length is described. This demonstrates for the first time the viability of generating long sequence heparanoids by chemical synthesis, *via* practical solution-phase synthesis. Pure-Shift HSQC NMR provides a dramatic improvement in anomeric signal resolution, allowing full resolution of all 12 anomeric protons and extrapolation to support anomeric integrity of the longer species. A chemically pure 6-*O*-desfulfated GlcNS-IdoAS icosasaccharide (20-mer) represents the longest pure synthetic heparin-like oligosaccharide.

## Abstract 2

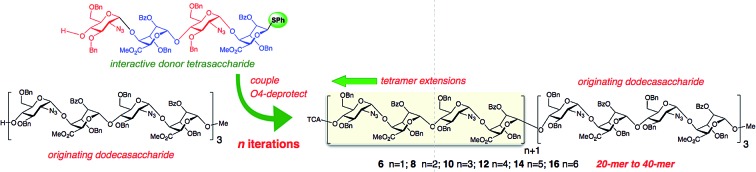
Synthesis of the longest heparin-related oligosaccharide backbones is enabled by efficient iterative [4]_*n*_-mer homologations *via* solution-phase synthesis. Pure-Shift HSQC NMR provides a dramatic improvement in anomeric signal resolution.

## Introduction

The synthesis of long sequence oligosaccharides (≥10 monosaccharide units) is an area of enduring challenge for synthetic carbohydrate chemistry across a diversity of oligosaccharide types. Despite advances in synthetic methods and recent progress in supported and automated chemistry,^1^–^5^ access to longer oligosaccharides remains highly demanding in both synthetic input and in characterization.

Heparin and heparan sulfate (H/HS) are highly polydisperse examples of glycosaminoglycan (GAG) saccharides, structurally characterized by alternating 1 → 4-linked d-glucosamine and uronic acid units, the latter constituting either d-glucuronic or l-iduronic acid. H/HS typically occurs in oligomers up to 40–50 units in length and though native oligomers range to >150 in length, most biological recognition relates to shorter oligomers, spanning the 6–40-mer range. Additionally, the backbone is modified by a variety of different sulfation patterns, mostly involving the 2-amino and O6 of d-GlcN and the O2 of the uronic acid, with rarer d-GlcN O3 sulfation, and are organized into low- and high-sulfation domains with heparin typified by higher iduronate content and sulfation than heparan sulfate. These oligosaccharides are known to bind to over one hundred proteins and play many diverse regulatory roles in biology, including angiogenesis, inflammation, stem-cell differentiation, pathogen infectivity and are also essentially involved in chemokine biology, regulating oligomerization dynamics and cell-migration properties.^6^–^8^ There is considerable interest in the potential to define therapeutically-viable H/HS sequences,^9^,^10^ underscored by the development of Arixtra (Fondaparinux) as a synthetic anti-coagulant heparin fragment,^11^,^12^ and advances in biocatalytic access to longer sequence replacements for medicinal heparin.^13^,^14^

The diverse structural microheterogeneity of the natural oligosaccharides and these pervasive regulatory roles ensure that defining the effects of specific structural differences on binding and biological effects of H/HS sequences remains at the forefront of carbohydrate chemical biology, whilst also amongst the most structurally-diverse and functionally-complex of oligosaccharide targets. There is considerable evidence that native H/HS oligosaccharides interact with various heparin-binding proteins across a wide range of oligomer lengths. Recent structural work has shown that even H/HS disaccharides can provide binding selectivity for the important growth factors FGF1 15 and FGF2,^16^ however, sequences of 8–12 monosaccharides are established as essential lengths for many HS-mediated biological effects. Protein binding selectivity does not necessarily correlate to observed biological effects, which involve more complex processes of assembling HS ligand–protein–receptor complexes, including in some cases essential dimerization, to effect biological signaling. The nature of the uronic acid residues is established as central to biological roles,^17^ and there is good evidence of biological effects of sulfation levels of GlcN O6.^18^ With respect to structurally-pure synthetic GAGs, prior work has indicated that homogenous sulfation levels and locations can also affect the binding preferences of other GAGs to target proteins^19^–^21^ and there is importance in revealing evidence for differential *in vitro* and *in vivo* biological consequences of changes in sulfation locations.^13^

Synthetic access to chemically pure H/HS species (most typically with targets in the 4–8 length range^22^–^34^) has been critical to advancing understanding of HS chemical and structural biology. Syntheses of H/HS sequences, however, remains technically challenging with typical requirements for lengthy routes. This, combined with the inclusion of iduronate as the uronic acid in many biologically-relevant H/HS oligosaccharides, has until recently limited access to small scale, particularly for sequences ≥8-mer. In addition to our own recent reports (*vide infra*) several other syntheses of H/HS-related 10-mer and 12-mer oligosaccharides have been described,^13^,^35^,^36^ whilst the recent development of multi-enzymatic routes have also added to strategies for access to the ≤12 length heparanome, offering new potential towards engineered H/HS targets.^13^,^37^

Longer H/HS sequences are involved in a number of significant interactions, some relevant to protein dimerization implicit in signalling processes and central to regulation of chemokine-mediated biology. The monomer–dimer equilibria, critical to regulating the biological effects of interleukin-8 (CXCL8), is believed to involve binding to HS sequence(s) of >20 units.^38^ The natural occurrence of H/HS across the longer heparanome (16–40-mers) almost certainly means that a diversity of other critical biological interactions involve longer sequence interactions. Viable synthetic access to structurally-homogeneous longer H/HS sequences as defined molecular tools would facilitate advancing our understanding of unexplored GAG chemical biology of the longer heparanome.

Synthetic access to the longer heparanome backbones has been elusive. A review has reported a 16-mer^39^ and a dimeric 8-mer [equivalent to 16-mer] has been described^40^ whilst the seminal work in addressing longer heparin-mimetics by chemical synthesis remains the synthesis of a series of glucose-based GAG mimetics up to the 20-mer,^41^ which, though providing valuable tools to probe factor Xa/thrombin effects, is not a native H/HS sequence. Chemoenzymatic examples of a mixed heparin-like 21-mer were also reported^37^ and examples of the native-type longer heparin-like backbones beyond this, have not hitherto been reported by chemical syntheses.

Recent advance in the area of less functionalized GAGs is highlighted by a report on the synthesis of hyaluronic acid, a simpler unsulfated GAG constituting a repeating GlcA-GlcNAc unit, up to a 15-mer.^1^

We recently described a scalable iduronate synthesis facilitating multi-gram access to GlcN-IdoA disaccharides suitable for oligosaccharide homologation,^42^ and synthesis of several different heparin-related oligosaccharides up to 12-mers. This included homogeneously fully 6-*O*-desulfated 12-mer on gram scale,^43^ and the first synthetic per-6-*O*-sulfated LMWH-like 12-mer.^44^ However, even scalable and efficient +[2]_*n*_ iterations are likely to become limited in practicability for access to substantively longer sequences due to pathway length, separation convergence and possible cumulative isomerism. We also therefore recently reported the synthesis of a related heparin-like dodecasaccharide by replacing disaccharide iteration with a GlcN-IdoA-GlcN-IdoA tetrasaccharide block approach, exploiting the utility of iduronate thioglycoside donor capability at the tetrasaccharide, rather than disaccharide, level,^45^ to demonstrate a shorter 2-cycle access to a dodecasaccharide target. The efficiency and selectivity of these +[4]_*n*_ iterations, coupled with our multi-gram scale access to tetrasaccharide modules, suggested a +[4]_*n*_ block iteration strategy might provide a viable homologation to substantially longer GAG sequences, which have not been addressable hitherto. Block synthesis has proven effective in a number of elegant branched oligosaccharide syntheses,^46^ but has not seen application to GAG oligosaccharides above dodecasaccharides. Here we report an iterative tetrasaccharide block chemical synthesis providing the first access to all the [4]_*n*_ heparin-like [GlcN-IdoA]_*n*_ backbone oligosaccharides in the 20 to 40-mer range, which had previously eluded synthetic access. This more than doubles the length of heparin-like oligosaccharides which have previously been amenable to chemical synthesis, and also provides the longest synthetic oligosaccharides (32-mer, 36-mer and 40-mer) of any type yet reported by solution or by solid-supported methods, exceeding the recent advance reported for small scale provision of a uniform non-GAG 30-mer oligomannoside,^2^ and the solution-phase 28-mer reported by Fraser-Reid.^47^ Application of Pure-Shift HSQC NMR data resolution enhancement is applied to confirm high anomeric integrity and the first chemical synthesis of a native heparin-like NS, 2S-20-mer is also reported.

## Results and discussion

### Strategy

**Fig. 1:**
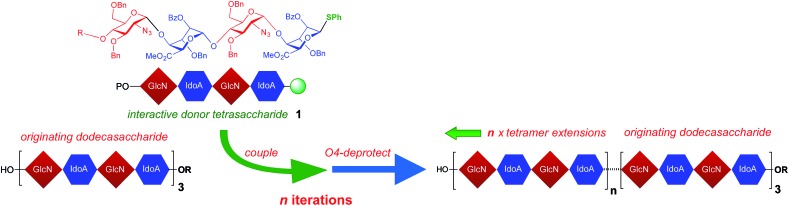
Strategy for iterative long heparin-like oligomer syntheses *via* tetrasaccharide **1** block homologation.

We sought to evaluate the use of a [GlcN-IdoA]_2_ tetrasaccharide donor **1** 45 for iterative homologation, proceeding from our previously-described^43^ scalable 12-mer synthesis, to deliver the first examples of synthetic heparin-like oligosaccharide sequences up to ≥20-mer (Fig. 1), ideally spanning the longer native heparanome lengths.

It was also important to demonstrate that this route to extended oligosaccharides could deliver usable quantities of materials with reproducible homologation efficiencies and anomeric integrity throughout the sequence. For all oligosaccharide synthetic routes the reproducibility of coupling efficiency (and anomeric integrity) for longer chains is a recurrent challenge. Similarly, convergence of separability of product from incompletely glycosylated long acceptors presents difficulties as the sequences become longer. We reasoned that application of our block strategy could assist both aspects, by significantly reducing the number of sequential steps (fewer glycosylations and decreasing cycles for any accumulating loss of anomeric coupling integrity) and providing a more significant structural change upon each homologation step (thus aiding separation and purification).

### Iterative synthesis of longest oligosaccharides

**Fig. 2:**
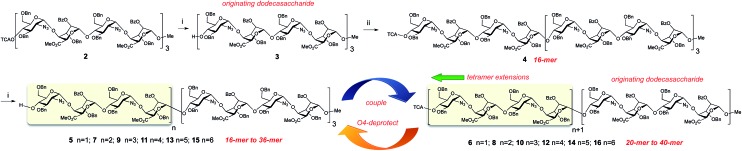
Elongation from 12-mer **3** through to 40-mer **15**. (i) MeOH, pyridine, (ii) NIS, AgOTf (cat.), DCM, **1**. *n* = 1: **4**, 76%, **5**, 90%; *n* = 2: **6**, 81%, **7**, 97%; *n* = 3: **8**, 77%, **9**, 95%; *n* = 4: **10**, 75%, **11**, 93%; *n* = 5: **12**, 78%, **13**, 93%; *n* = 6: **14**, 72%, **15**, 87%, **16**, 64%; *n* = 7.

A coupling–deprotection cycle of two-step homologations using donor tetrasaccharide **1** 45 was envisioned for extending oligosaccharides from the dodecasaccharide level in a +4 iteration sequence. Starting from O4-protected dodecasaccharide **2** 43 selective release of O4 afforded the starting dodecasaccharide acceptor **3** in very high yield (Fig. 2). The subsequent homologations to 16-mer and thence to 20-mer proceeded in very good overall yield (68% and 79% respectively) for each two-step homologation. These steps could be performed on practicable scale, providing 300 mg of this novel 20-mer. This is a significant scale of synthesis for any oligosaccharide intermediate even at shorter lengths, and is essentially underpinned by our multi-gram access to tetrasaccharide **1** resulting from scalable iduronate synthesis.^42^,^43^ With this effective homologation to 20-mer in hand, we then sought to evaluate further iterations. The glycosylations of increasingly long acceptors using **1** proved reliable throughout a further series of +[4] iterations affording the 24-mer, 28-mer, 32-mer, 36-mer and 40-mer. The homologations proceeded in high yields both at the glycosylation and O4-deprotection steps (56–78% yield over the two steps in each cycle, 8% overall for 13 steps) and with high anomeric selectivity (Fig. 2).

To demonstrate that these syntheses could be performed on usable scales, each of these homologations delivered 100–300 mg samples of protected oligosaccharides through the entire 16-mer to 32-mer series and at 30–76 mg scale for the 36-mer and 40-mer. These are substantive scales for syntheses of oligosaccharides of this type, and would be challenging even for considerably shorter heparin-like sequences.

This series also compares favourably to the automated chemical synthesis of the longest monosaccharide-based oligosaccharide, which provides mg amounts of a partially protected (non-GAG) oligomannoside.^2^ Purification of all long heparins **4–15** by standard chromatography proved effective, employing our previously reported toluene/acetone solvent system,^43^ also evidencing that the block approach assists the viability of solution phase synthesis and purification by greater substrate-product differentiations (ESI Fig. 74†). A further aid to purification was the insolubility of longer oligosaccharides in EtOAc/hexane mixtures, making precipitation a reliable method to remove unwanted small molecule traces from reactions.

This establishes a chemical synthesis of the longest synthetic oligosaccharides of any structural type yet reported and also significantly increases the length of synthetic heparanoid oligosaccharides now accessible by chemical synthesis. This provides a paradigm-shift in planning access to the longer heparanome by demonstrating an effective solution-phase strategy that can both span the entire target length range of likely biological relevance and do so on viable scales.

### Structural characterization and sequence anomeric integrity

**Fig. 3:**
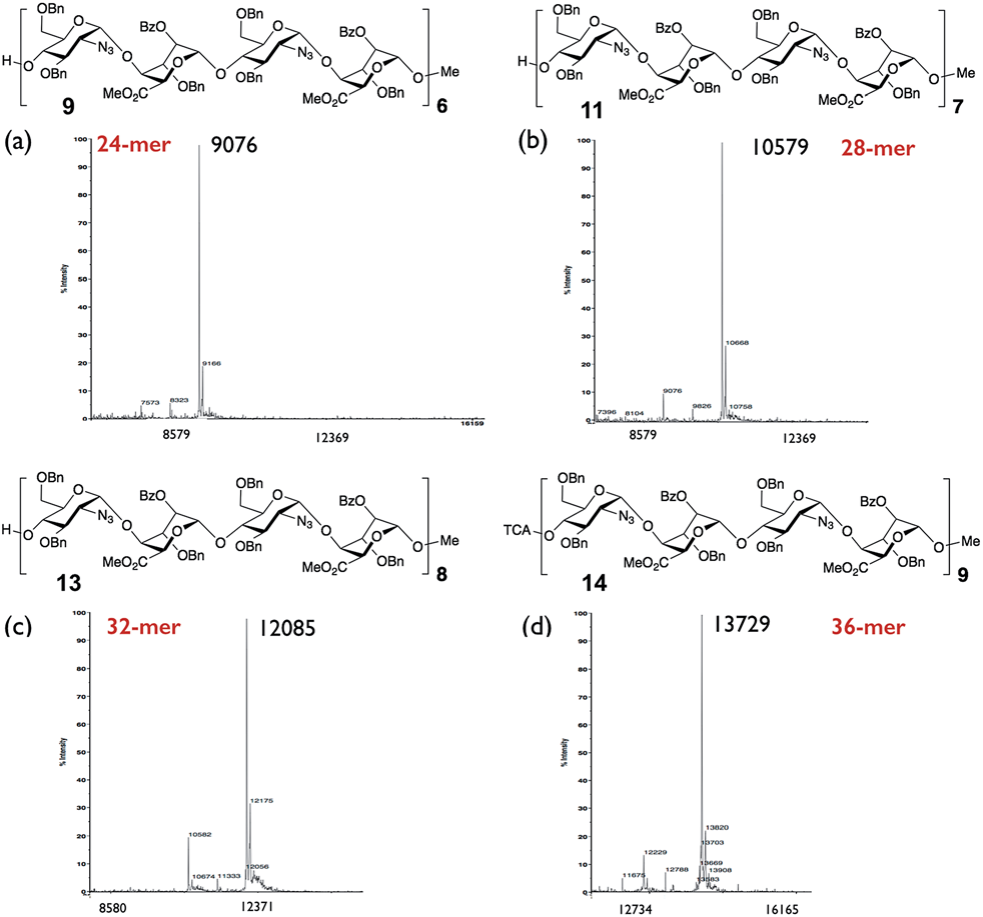
MALDI mass spectra of protected long oligosaccharides. (a) 24-mer, **9**. (b) 28-mer, **11**. (c) 32-mer, **13**. (d) 36-mer, **14**.

Mass spectral data for the series of oligosaccharides provided convincing supporting data for structure, with molecular ions present for [M + Na]^+^ as the base peak for all species, illustrated in Fig. 3 by base peaks for the series of longest oligosaccharides across the 24-mer, 28-mer, 32-mer and 36-mer series (see also: ESI Fig. 39, 46, 53 and 58†), at this level of oligomerization these fall within the macromolecule region (9–13 kDa).

**Fig. 4:**
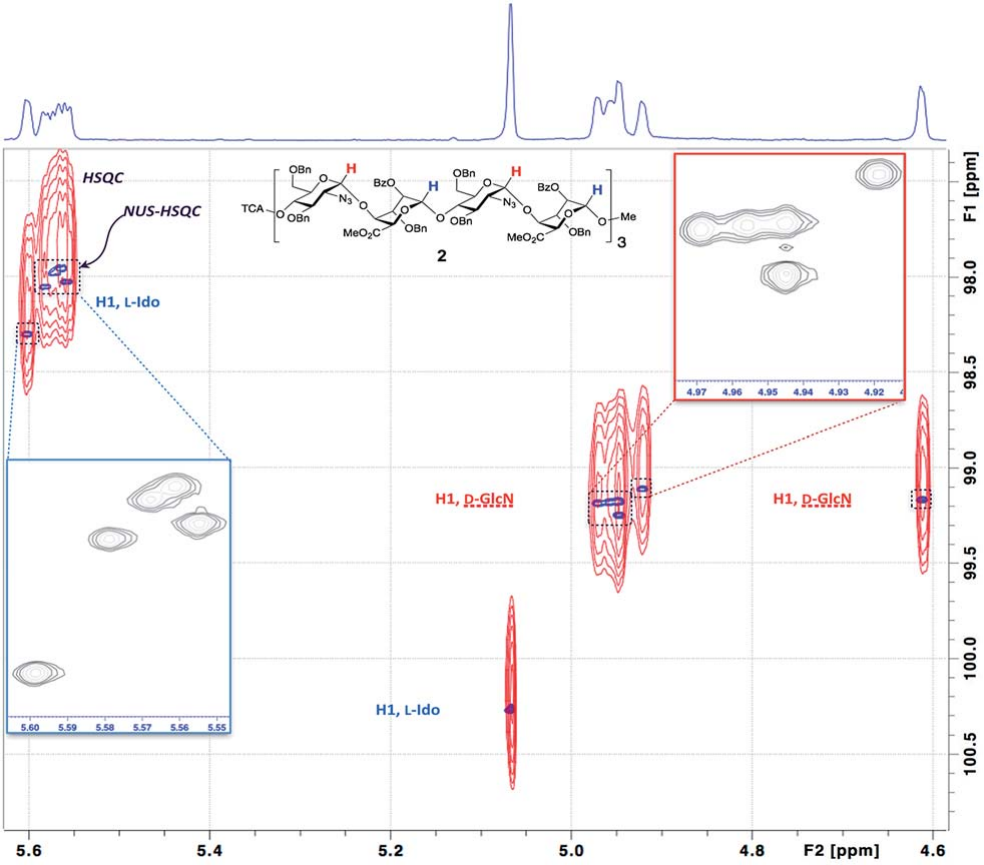
NUS Pure Shift HSQC spectra of dodecasaccharide **2**. Overlay of anomeric regions from standard HSQC (cross-peaks in red) *vs.* NUS Pure Shift HSQC (cross-peaks in blue), allowing resolution of all 12 anomeric protons NUS Pure Shift HSQC expanded as inserts.

**Fig. 5:**
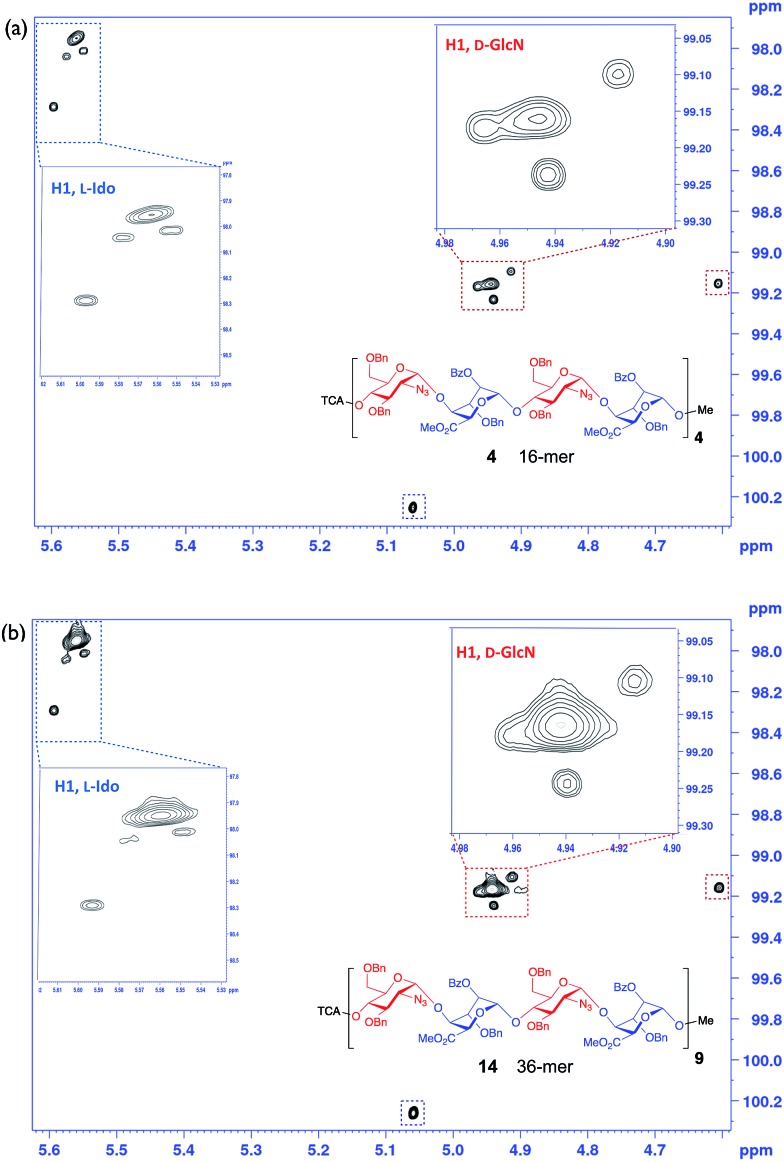
NUS HSQC 800 MHz data for long oligosaccharides: (a) 16-mer, **4**. (b) 36-mer, **14**. Inset expansions.

We wished to use NMR to confirm the anomeric integrity across this series. Significant resolution enhancement of the twelve anomeric resonances for the 12-mer we reasoned may then facilitate extrapolation to provide sufficient resolution improvement for the longer series, important for defining anomeric integrity in the longer sequence syntheses. NMR confirmation of the purity of the final products was attempted prior to deprotection of the sugar hydroxyls, the protecting aromatic groups providing extra dispersion through ring current shifts, and also low viscosity organic solvent (CDCl_3_) resulting in faster correlation times and therefore sharper linewidths.§
§The poorer linewidths and dispersion in a deprotected, aqueous sample of the 12-mer precluded resolution of all the anomeric signals, even using NUS-HSQC. However, to record 2D spectra with sufficient acquisition time in the indirect dimension to resolve all the peaks would have taken a prohibitively long time, so non-uniform sampling (NUS)^48^ of the indirect dimension of the ^1^H^13^C-HSQC spectra was employed. Resolution was improved further by homonuclear decoupling during acquisition using a BIRD pulse train^49^ (Fig. 4 and 5).

This provided dramatic enhancement to achieve sufficient resolution of all twelve anomeric protons (Fig. 4). The signal to noise ratio in the 2D-HSQC of the ^1^H^13^C-HSQC for dodecasaccharide **2** was ∼50 : 1 and as there is an absence of any resonances arising from the other possible anomeric linkages the stereoselectivity of the synthesis can thus be defined as better than 98% (Fig. 4; ESI Fig. 55–57†). In the longer oligomers >12-mer, the similarity in chemical environment in the central units results in little dispersion of those additional anomeric resonances, which precludes further resolution by NMR, but the crosspeak intensities for these atoms scale consistently with the number of monomers in each oligomer (ESI Fig. 57b†). Furthermore, there are evidently no novel cross peaks in the HSQC spectra of the longer oligomers, supporting the extrapolation and indicating a very high level of anomeric fidelity (Fig. 5).

### Accessing synthetic 2-*O*-sulfated and 2-*O*,*N*-sulfated 20-mers

**Fig. 6:**
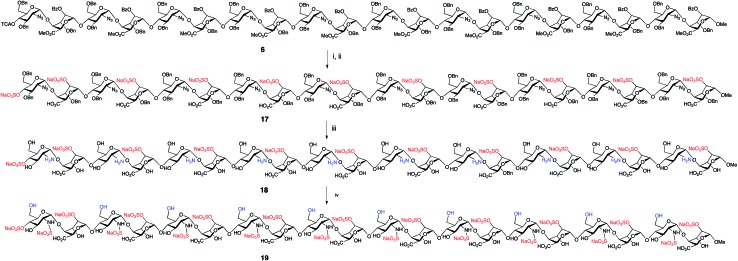
Deprotection and sulfation to afford homogeneous heparin-like 20-mers [GlcNH_2_-IdoA2S]_10_-OMe, **18** and [Glc6SNS-IdoA2S]_10_-OMe, **19**. (i) LiOH, THF/MeOH/H_2_O, 48%. (ii) NMe_3_˙SO_3_, DMF, μW,^44^ 90%. (iii) Pd(OH)_2_/C, EtOH/H_2_O, H_2_, 89%. (iv) Pyridine SO_3_ complex, H_2_O.

**Fig. 7:**
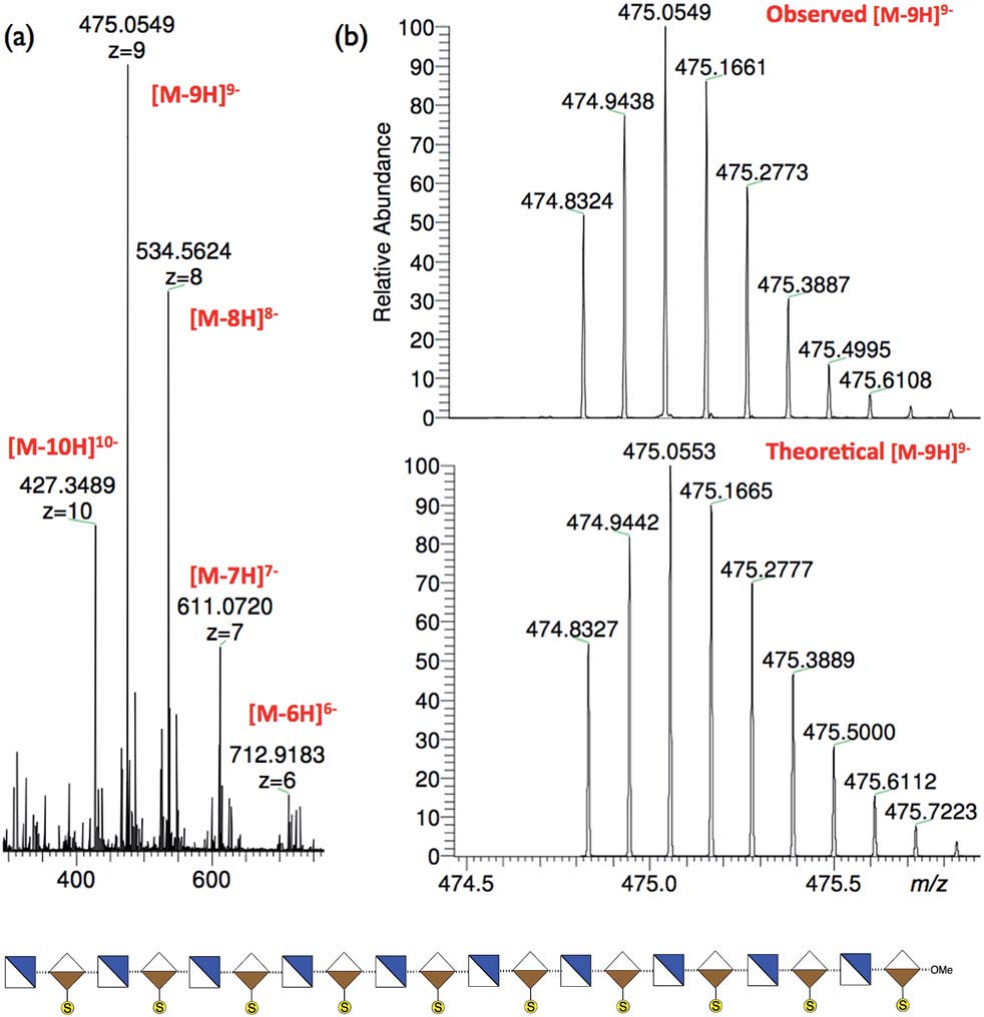
Nano-electrospray mass spectral data for icosasaccharide [GlcNH_2_-IdoA2S]_10_-OMe **18**. (a) Main ions observed for *z* = 7 (27%), 8 (72%), 9 (100%) and 10 (41%). (b) Observed and theoretical isotope patterns for major ion [M – 9H]^9–^ (LTQ Oribitrap XL).

To illustrate access to sulfated, biologically-relevant, long heparanoid systems we selected fully protected icosasaccharide **6** to evaluate conversion to the native heparin-type *N*- and 2-*O*-sulfated 20-mer **19** (Fig. 6) *via* 20-mer **18**. The efficiency of the polysulfation steps and purification of longer sulfated structures are often challenging in H/HS syntheses. Gratifyingly, 20-mer **6** (40 mg) was saponified, *O*-sulfated and hydrogenated^43^–^45^ in 38% overall yield for three steps to afford 2-*O*-sulfated, *N*- and 6-*O*-desulfated icosasaccharide amine (A4I2-[A0I2]_9_)-OMe^50^**18** (Fig. 6). This material was characterized using nanoelectrospray MS (Fig. 7), which showed multiple signals for different charge states of the expected parent ion, along with matching isotope patterns, fully consistent with successful installation of all *O*-sulfate groups. Additionally, 800 MHz NMR spectra confirmed complete hydrogenolysis of benzyl protecting groups and reduction of azides to deliver the structurally homogenous **18** (see ESI Fig. 64–66†).

**Fig. 8:**
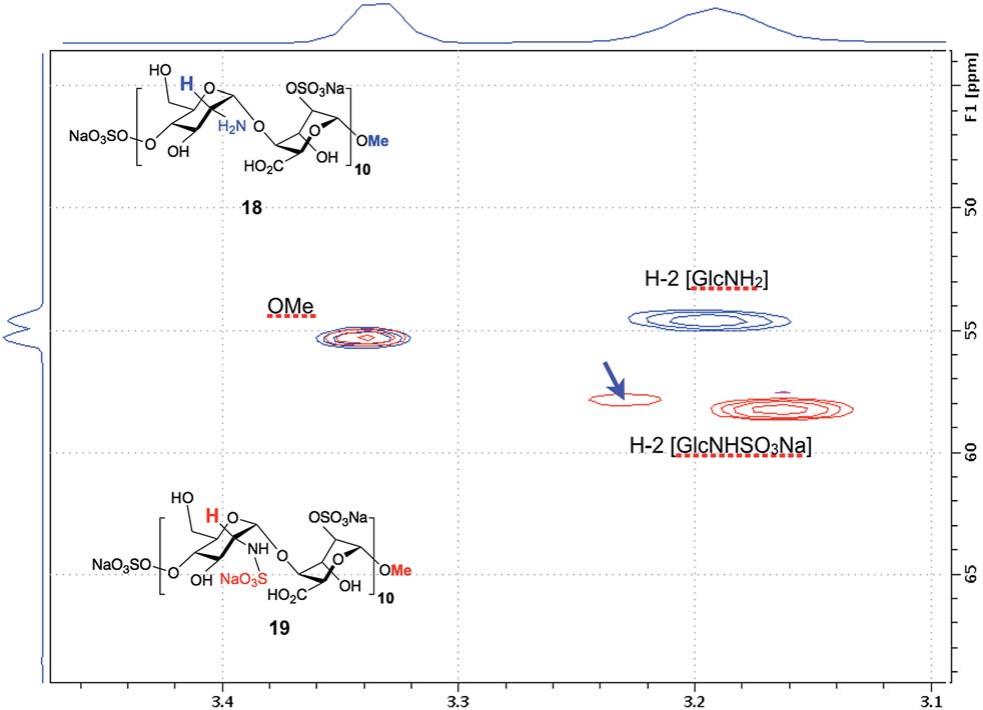
NMR analysis of GlcN *N*-sulfation of icosasaccharide (A4I2-[A0I2]_9_)-OMe **18** to icosasaccharide (S4I2-[S0I2]_9_)-OMe **19**. Overlay spectra for **18** (blue) and **19** (red), show coincidence of terminal methoxy resonances, and definitive shift of H2 signals due to *N*-2-sulfation in **19**. Resolved single GlcNS marked by blue arrow (see ESI Fig. S70†).

The *O*-2-sulfated icosasaccharide (A4I2-[A0I2]_9_)-OMe **18** was then *N*-sulfated to provide the IdoA 2-*O*- and glucosamine *N*-sulfated (S4I2-[S0I2]_9_)-OMe 20-mer **19** in 93% yield. Shift of H2 signals confirm no remaining NH_2_ sites (Fig. 8) with two H2 NS signals appearing for **19**, and that the minor of which equates to approximately one residue was confirmed by COSY and HSQC data (ESI Fig. 70†). This is the first synthetic heparin-like material of this length reported. The efficiency and completeness of the *N*-sulfation was confirmed by 800 MHz NMR (Fig. 8 and ESI Fig. 70–72†) evidenced by the definitive shift of the H2 signals for the GlcN rings. PAGE analysis showed close comparison to commercial digest heparin dp20, but with higher purity (ESI Fig. 73†). Such a process provides, for the first time, a synthetic access to two homogeneous 20-mer HS-like oligosaccharides of differing native-like sulfation levels, redefining the boundaries for a solution phase approach to native HS systems.

## Conclusions

The first purely synthetic chemical approach to provide native-backbone heparin-like oligosaccharides in the longer heparanome (>16-mer) is reported. This demonstrates that a tetrasaccharide block-wise iterative approach enables syntheses up to and including the longest synthetic oligosaccharide (of any type) yet described (40-mer). This now facilitates chemical synthesis to this previously inaccessible area of glycospace with sequence homogeneity. This work shows that adapting solution phase synthesis using block-synthetic modules can provide a practicable route to long saccharides of high purity and stereochemical integrity. This report also describes the chemical synthesis of a pure heparin-like *N*,*O*-2-sulfated icosasaccharide (20-mer) which has significance for the current importance of developing pure synthetic heparin-type replacements. Additionally, this report describes an important advance in NMR structural assignment at 12-mer level and quantification of anomeric integrity in long oligosaccharides. NUS Pure-Shift HSQC is shown to provide very dramatic resolution enhancement and was applied to (i) provide the first clear resolution of the 12 anomeric signals for a synthetic heparin-related dodecasaccharide and (ii) thereby allowed comparative data up to a synthetic 36-mer providing direct, robust NMR support regarding the anomeric integrity in these very long synthetic oligosaccharides. This will have value for the analysis of other long synthetic oligosaccharide systems.

## Supplementary Material

Supplementary information
