# Supplementary material for: Making the longest sugars: a chemical synthesis of heparin-related [4]n oligosaccharides from 16-mer to 40-mer
Source: Chem Sci. 2015 Jul 24;6(11):6158–64. doi: 10.1039/c5sc02091c (PMC6054106; doi:10.1039/c5sc02091c)
Supplement: Supplementary file 1 [file SC-006-C5SC02091C-s001.pdf]

Electronic Supplementary Material (ESI) for Chemical Science.
This journal is © The Royal Society of Chemistry 2015

Making the longest sugars: A chemical synthesis of heparin-related $[4]_n$ oligosaccharides from 16-mer to 40-mer

Steen U. Hansen,[†1] Gavin J. Miller,[†1] Matthew J. Cliff,[2] Gordon C. Jayson[3] and John M. Gardiner[1*]

Electronic Supplementary Information

1. Experimental procedures: Synthesis

General methods

All the chemicals used were purchased from commercial sources without further purification. All reactions were monitored by TLC on Merck silica gel plates 60 F254. Silica gel 60 (particle size 0.035-0.070 mm) was used for column chromatography. 1H NMR spectra were recorded at 400 or 800 MHz and 13C spectra at 100 or 200 MHz respectively on Bruker DPX spectrometers. Mass spectra (MS) were recorded using a Micromass Platform II spectrometer using an electro spray ionization source or via the EPSRC National Mass Spectrometry Service (Swansea). Isotope patterns for compounds with mass > 1000 are included in the SI. Infrared spectra were obtained by using a Bruker Alpha instrument. Melting points were determined using Stuart Scientific SMP10 apparatus and are uncorrected. Optical rotations were obtained using an AA-1000 polarimeter. Elemental analyses were performed by Micro Analytical Laboratory, School of Chemistry, The University of Manchester. NMR Data were reprocessed using iNMR 4 from Nucleomatica or Topspin. Lass spectra repot monoisotopic values where indicated, and most probable mass otherwise. Microwave reactor was a CEM Microwave Organic Synthesis Reactor. LCMS data were recorded using a LC-MSD-Trap-SL using a Supelco Ascentis C8, 5 micron column (4.6 x 30mm).

Methyl (2-azido-3,6-di-*O*-benzyl-2-deoxy-α-D-glucopyranosyl)-(1⟶4)-(methyl 2-*O*-benzoyl-3-*O*-benzyl-α-L-idopyranosyl uronate)-(1⟶4)-2-azido-3,6-di-*O*-benzyl-2-deoxy-α-D-glucopyranosyl)-(1⟶4)-(methyl 2-*O*-benzoyl-3-*O*-benzyl-α-L-idopyranosyl uronate)-(1⟶4)-2-azido-3,6-di-*O*-benzyl-2-deoxy-α-D-glucopyranosyl-(1⟶4)-(methyl 2-*O*-benzoyl-3-*O*-benzyl-α-L-idopyranosyl uronate)-(1⟶4)-2-azido-3,6-di-*O*-benzyl-2-deoxy-α-D-glucopyranosyl-(1⟶4)-(methyl 2-*O*-benzoyl-3-*O*-benzyl-α-L-idopyranosyl uronate)-(1⟶4)-2-azido-3,6-di-*O*-benzyl-2-deoxy-α-D-glucopyranosyl-(1⟶4)-(methyl 2-*O*-benzoyl-3-*O*-benzyl-α-L-idopyranosyl uronate)-(1⟶4)-2-azido-3,6-di-*O*-benzyl-2-deoxy-α-D-glucopyranosyl-(1⟶4)-(methyl 2-*O*-benzoyl-3-*O*-benzyl-α-L-idopyranoside) uronate, 3

The dodecasaccharide **2** (452 mg, 0.096 mmol) was dissolved in a mixture of MeOH/pyridine (5 mL/2 mL) and heated to 50 °C for 7 h. The solvents were evaporated and co-evaporated with toluene (2x10 mL). The crude product was purified using flash column chromatography (EtOAc/hexane gradient 3:5 to 2:3). This yielded **3** (416 mg, 95%) as a white foam. R_f 0.19 (toluene/

[1]Manchester Instutute of Biotechnology, School of Chemistry, Faculty of EPS, The University of Manchester 131 Princess Street, , Manchester M1 7DN, UK. [2]Manchester Institute of Biotechnology, The University of Manchester 131 Princess Street, Manchester M1 7DN, UK. [3]Institute of Cancer Sciences, The University of Manchester, Wilmslow Road, Manchester M20 4BX, UK. [†]Equal contributions.

acetone 10:1). $[\alpha]_D^{20}$ = +21.5 (*c* = 0.26, CH_2Cl_2). ^1H NMR (400 MHz; $CDCl_3$): δ 8.10-8.08 (m, 2H, Bz), 7.98-7.90 (m, 10H, Bz), 7.53-6.98 (m, 108H, Ph), 5.60-5.52 (m, 5H, H^{CEGIK}-1), 5.22-5.15 (m, 5H, H^{CEGIK}-2), 5.07-5.06 (m, 1H, H^A-2), 5.04-5.03 (m, 1H, H^A-1), 4.99-4.87 (m, 6H, H^{BDFHJL}-1), 4.83-4.30 (m, 42H, H^{ACEGIK}-5, 18xCH_2Ph), 4.25-4.15 (m, 5H, H^{CEGIK}-3), 4.06-3.16 (m, 43H, H^A-3, H^{ACEGIK}-4, H^{BDFHJL}-2, H^{BDFHJL}-3, H^{BDFHJL}-4, H^{BDFHJL}-5, H^{BDFHJL}-6_{ab}), 3.47 (s, 3H, COOCH_3), 3.46 (s, 3H, COOCH_3), 3.44 (s, 3H, COOCH_3), 3.29 (s, 3H, COOCH_3), 3.27 (s, 3H, COOCH_3), 3.26 (s, 3H, COOCH_3), 3.22 (s, 3H, OCH_3). ^{13}C NMR (101 MHz; $CDCl_3$): δ 169.6, 169.3, 169.3, 169.2, 165.6, 165.2, 165.1, 138.0, 137.9, 137.9, 137.8, 137.8, 137.7, 137.6, 137.4, 137.4, 137.3, 133.6, 133.5, 129.9, 129.9, 129.8, 129.5, 129.3, 129.3, 129.2, 128.8, 128.7, 128.6, 128.6, 128.4, 128.3, 128.2, 128.2, 128.2, 128.1, 128.0, 128.0, 127.9, 127.8, 127.8, 127.7, 127.6, 127.5, 127.4, 127.3, 127.3, 100.3, 99.3, 99.2, 99.2, 98.1, 98.0, 98.0, 98.0, 79.2, 78.3, 78.2, 78.1, 77.4, 77.3, 77.0, 76.7, 75.9, 75.8, 75.8, 75.6, 75.5, 75.5, 75.4, 75.3, 74.8, 74.8, 74.5, 74.4, 74.2, 74.1, 74.1, 73.8, 73.7, 73.6, 72.4, 72.4, 72.3, 71.3, 71.2, 71.1, 70.1, 70.6, 70.5, 70.5, 70.4, 70.4, 69.4, 67.9, 67.3, 67.3, 67.3, 67.3, 67.2, 67.1, 67.1, 63.4, 63.1, 63.0, 62.7, 56.2, 52.1, 52.0, 51.7, 51.6. MALDI TOF MS: monoisotopic m/z: calcd for $C_{247}H_{250}N_{18}NaO_{67}$ $[M+Na]^+$: 4562.7; found: 4562.6.

Methyl (2-azido-3,6-di-*O*-benzyl-2-deoxy-4-*O*-trichloroacetyl-α-D-glucopyranosyl)-(1→4)-(methyl 2-*O*-benzoyl-3-*O*-benzyl-α-L-idopyranosyl uronate)-(1→4)-2-azido-3,6-di-*O*-benzyl-2-deoxy-α-D-glucopyranosyl-(1→4)-(methyl 2-*O*-benzoyl-3-*O*-benzyl-α-L-idopyranosyl uronate)-(1→4)-2-azido-3,6-di-*O*-benzyl-2-deoxy-α-D-glucopyranosyl-(1→4)-(methyl 2-*O*-benzoyl-3-*O*-benzyl-α-L-idopyranosyl uronate)-(1→4)-2-azido-3,6-di-*O*-benzyl-2-deoxy-α-D-glucopyranosyl-(1→4)- (methyl 2-*O*-benzoyl-3-*O*-benzyl-α-L-idopyranosyl uronate)-(1→4)-2-azido-3,6-di-*O*-benzyl-2-deoxy-α-D-glucopyranosyl-(1→4)-(methyl 2-*O*-benzoyl-3-*O*-benzyl-α-L-idopyranosyl uronate)-(1→4)-2-azido-3,6-di-*O*-benzyl-2-deoxy-α-D-glucopyranosyl-(1→4)-(methyl 2-*O*-benzoyl-3-*O*-benzyl-α-L-idopyranosyl uronate)-(1→4)-2-azido-3,6-di-*O*-benzyl-2-deoxy-α-D-glucopyranosyl-(1→4)-(methyl 2-*O*-benzoyl-3-*O*-benzyl-α-L-idopyranosyl uronate)-(1→4)-2-azido-3,6-di-*O*-benzyl-2-deoxy-α-D-glucopyranosyl-(1→4)-(methyl 2-*O*-benzoyl-3-*O*-benzyl-α-L-idopyranoside) uronate, 4

Acceptor decasaccharide **3** (114 mg, 0.025 mmol) and thioglycoside tetrasaccharide donor **1** (53 mg, 0.030 mmol) was dissolved in dry DCM (1 mL) under N_2. Freshly activated 4Å powdered molecular sieves (130 mg) was added and the solution cooled to 0 °C in an icebath. After 10 min. NIS (19 mg, 0.084 mmol) was added, and after another 10 min. AgOTf (catalytic amount) was added. The suspension changed colour from pale yellow to deep red, was stirred for 30 min. and the reaction was quenched into a separating funnel containing a mixture of DCM (30 mL), saturated aqueous $NaHCO_3$ (20 mL) and $Na_2S_2O_3$ (1 mL, 10% aqueous). After shaking until the iodine colour was removed the suspension was filtered through a short pad of Celite® washing with water and DCM. The layers were separated and the aqueous extracted with DCM (10 mL). The organic layers were combined, dried ($MgSO_4$) and solvent removed *in vacuo*. The crude was purified by silica gel flash column chromatography (toluene/acetone 20:1) to give **4** (118 mg, 76%) as a white foam. R_f 0.32 (toluene/acetone 10:1). $[\alpha]_D^{20}$ = +31.9 (*c* = 0.45, CH_2Cl_2). ^1H NMR (400 MHz; $CDCl_3$): δ 8.15-8.13 (m, 2H, Bz), 8.07-8.05 (m, 2H, Bz), 8.00-7.96 (m, 12H, Bz), 7.57-7.02 (m, 144H, Ph), 5.64-5.56 (m, 7H, $H^{CEGIKMO}$-1), 5.35 (t, *J* = 9.6 Hz, 1H, H^P-4), 5.25-5.18 (m, 7H, $H^{CEGIKMO}$-2), 5.12-5.11 (m, 1H, H^A-2), 5.08-5.07 (m, 1H, H^A-1), 4.99-4.91 (m, 8H, $H^{BDFHJLNP}$-1), 4.86-4.35 (m, 56H, $H^{ACEGIKMO}$-5, 24xCH_2Ph), 4.29-4.19 (m, 7H, $H^{CEGIKMO}$-3), 4.12-3.20 (m, 80H, H^A-3, $H^{ACEGIKMO}$-4, $H^{BDFHJLNP}$-2, $H^{BDFHJLNP}$-3, $H^{BDFHJLN}$-4, $H^{BDFHJLNP}$-5, $H^{BDFHJLNP}$-6_{ab}, 8xCOOCH_3), 3.27 (s, 3H, OCH_3). ^{13}C NMR (101 MHz; $CDCl_3$): δ 169.5, 169.3, 169.2, 165.5, 165.2, 165.1, 160.2,

138.0, 137.9, 137.8, 137.8, 137.7, 137.6, 137.4, 137.4, 137.4, 137.2, 137.2, 136.7, 133.6, 133.6, 133.5, 130.0, 129.9, 129.9, 129.8, 129.7, 129.5, 129.4, 129.3, 129.2, 129.0, 129.0, 128.9, 128.8, 128.7, 128.6, 128.5, 128.4, 128.3, 128.2, 128.2, 128.1, 128.1, 128.0, 127.9, 127.8, 127.8, 127.8, 127.7, 127.6, 127.5, 127.3, 125.3, 100.3, 99.2, 99.1, 98.3, 98.0, 98.0, 89.7, 78.2, 78.1, 78.0, 77.3, 77.3, 77.0, 77.0, 76.7, 76.7, 76.6, 75.9, 75.8, 75.7, 75.6, 75.5, 75.4, 75.3, 74.9, 74.8, 74.7, 74.5, 74.4, 74.3, 74.2, 74.1, 73.8, 73.7, 73.6, 72.4, 72.3, 71.3, 71.2, 71.1, 71.1, 70.6, 70.6, 70.5, 70.5, 70.5, 70.4, 70.4, 69.6, 69.2, 69.2, 68.0, 67.3, 67.3, 67.3, 67.2, 67.1, 67.1, 63.4, 63.2, 63.0, 56.2, 52.0, 51.9, 51.7, 51.7, 51.6. MALDI TOF MS: m/z: calcd for $C_{331}H_{331}Cl_3N_{24}NaO_{90}$ $[M+Na]^+$: monoisotopic m/z: 6209.1; found: 6209.1.

Methyl (2-azido-3,6-di-*O*-benzyl-2-deoxy-α-D-glucopyranosyl)-(1→4)-(methyl 2-*O*-benzoyl-3-*O*-benzyl-α-L-idopyranosyl uronate)-(1→4)-2-azido-3,6-di-*O*-benzyl-2-deoxy-α-D-glucopyranosyl-(1→4)-(methyl 2-*O*-benzoyl-3-*O*-benzyl-α-L-idopyranosyl uronate)-(1→4)-2-azido-3,6-di-*O*-benzyl-2-deoxy-α-D-glucopyranosyl-(1→4)-(methyl 2-*O*-benzoyl-3-*O*-benzyl-α-L-idopyranosyl uronate)-(1→4)-2-azido-3,6-di-*O*-benzyl-2-deoxy-α-D-glucopyranosyl-(1→4)-(methyl 2-*O*-benzoyl-3-*O*-benzyl-α-L-idopyranosyl uronate)- (1→4)-2-azido-3,6-di-*O*-benzyl-2-deoxy-α-D-glucopyranosyl-(1→4)-(methyl 2-*O*-benzoyl-3-*O*-benzyl-α-L-idopyranosyl uronate)-(1→4)-2-azido-3,6-di-*O*-benzyl-2-deoxy-α-D-glucopyranosyl-(1→4)-(methyl 2-*O*-benzoyl-3-*O*-benzyl-α-L-idopyranosyl uronate)-(1→4)-2-azido-3,6-di-*O*-benzyl-2-deoxy-α-D-glucopyranosyl-(1→4)-(methyl 2-*O*-benzoyl-3-*O*-benzyl-α-L-idopyranosyl uronate)-(1→4)-2-azido-3,6-di-*O*-benzyl-2-deoxy-α-D-glucopyranosyl-(1→4)-(methyl 2-*O*-benzoyl-3-*O*-benzyl-α-L-idopyranoside) uronate, 5

The hexadecasaccharide **4** (279 mg, 0.045 mmol) was dissolved in a mixture of MeOH/pyridine (3 mL/1 mL) and heated to 40 °C for 12 h. The solvents were evaporated and co-evaporated with toluene (2x10 mL). The crude product was purified using flash column chromatography (toluene/ acetone 20:1 to 15:1). This yielded **5** (245 mg, 90%) as a white solid. R_f 0.18 (toluene/acetone 10:1). $[\alpha]_D^{20}$ = +43.6 (*c* = 0.36, CH_2Cl_2). ^1H NMR (400 MHz; $CDCl_3$): δ 8.10-8.08 (m, 2H, Bz), 7.98-7.90 (m, 14H, Bz), 7.55-7.00 (m, 144H, Ph), 5.58-5.50 (m, 7H, $H^{CEGIKMO}$-1), 5.22-5.14 (m, 7H, $H^{CEGIKMO}$-2), 5.07-5.06 (m, 1H, H^A-2), 5.04-5.03 (m, 1H, H^A-1), 4.99-4.92 (m, 8H, $H^{BDFHJLNP}$-1), 4.87-4.33 (m, 56H, $H^{ACEGIKMO}$-5, 24xCH_2Ph), 4.24-4.15 (m, 7H, $H^{CEGIKMO}$-3), 4.06-3.17 (m, 81H, H^A-3, $H^{ACEGIKMO}$-4, $H^{BDFHJLNP}$-2, $H^{BDFHJLNP}$-3, $H^{BDFHJLNP}$-4, $H^{BDFHJLNP}$-5, $H^{BDFHJLNP}$-6_{ab}, 8xCOOCH_3), 3.22 (s, 3H, OCH_3). MALDI TOF MS: m/z: calcd for $C_{329}H_{332}N_{24}NaO_{89}$ $[M+Na]^+$: monoisotopic m/z: 6065.2; found: 6065.1.

Methyl (2-azido-3,6-di-*O*-benzyl-2-deoxy-4-*O*-trichloroacetyl-α-D-glucopyranosyl)-(1→4)-(methyl 2-*O*-benzoyl-3-*O*-benzyl-α-L-idopyranosyl uronate)-(1→4)-2-azido-3,6-di-*O*-benzyl-2-deoxy-α-D-glucopyranosyl-(1→4)-(methyl 2-*O*-benzoyl-3-*O*-benzyl-α-L-idopyranosyl uronate)-(1→4)-2-azido-3,6-di-*O*-benzyl-2-deoxy-α-D-glucopyranosyl-(1→4)-(methyl 2-*O*-benzoyl-3-*O*-benzyl-α-L-idopyranosyl uronate)-(1→4)-2-azido-3,6-di-*O*-benzyl-2-deoxy-α-D-glucopyranosyl-(1→4)- (methyl 2-*O*-benzoyl-3-*O*-benzyl-α-L-idopyranosyl uronate)-(1→4)-2-azido-3,6-di-*O*-benzyl-2-deoxy-α-D-glucopyranosyl-(1→4)-(methyl 2-*O*-benzoyl-3-*O*-benzyl-α-L-idopyranosyl uronate)-(1→4)-2-azido-3,6-di-*O*-benzyl-2-deoxy-α-D-glucopyranosyl-(1→4)-(methyl 2-*O*-benzoyl-3-*O*-benzyl-α-L-idopyranosyl uronate)-(1→4)-2-azido-3,6-di-*O*-benzyl-2-deoxy-α-D-glucopyranosyl-(1→4)-(methyl 2-*O*-benzoyl-3-*O*-benzyl-α-L-idopyranosyl uronate)-(1→4)-2-azido-3,6-di-*O*-benzyl-2-deoxy-α-D-glucopyranosyl-(1→4)-(methyl 2-*O*-benzoyl-3-*O*-benzyl-α-L-

idopyranosyl uronate)-(1→4)-2-azido-3,6-di-*O*-benzyl-2-deoxy-α-D-glucopyranosyl-(1→4)-(methyl 2-*O*-benzoyl-3-*O*-benzyl-α-L-idopyranosyl uronate)-(1→4)-2-azido-3,6-di-*O*-benzyl-2-deoxy-α-D-glucopyranosyl-(1→4)-(methyl 2-*O*-benzoyl-3-*O*-benzyl-α-L-idopyranoside) uronate, 6

Acceptor hexadecasaccharide **5** (286 mg, 0.047 mmol) and thioglycoside tetrasaccharide donor **1** (108 mg, 0.061 mmol) was dissolved in dry DCM (2 mL) under N_2. Freshly activated 4Å powdered molecular sieves (171 mg) was added and the solution cooled to 0 °C in an icebath. After 10 min. NIS (33 mg, 0.147 mmol) was added, and after another 10 min. AgOTf (catalytic amount) was added. The suspension changed colour from pale yellow to deep red, was stirred for 30 min. and the reaction was quenched into a separating funnel containing a mixture of DCM (30 mL), saturated aqueous $NaHCO_3$ (20 mL) and $Na_2S_2O_3$ (1 mL, 10% aqueous). After shaking until the iodine colour was removed the suspension was filtered through a short pad of Celite® washing with water and DCM. The layers were separated and the aqueous extracted with DCM (10 mL). The organic layers were combined, dried ($MgSO_4$) and solvent removed *in vacuo*. The crude was purified by silica gel flash column chromatography (toluene/acetone 20:1 to 15:1 to 10:1) followed by precipitation (dissolved in EtOAc (5 mL) and hexane (5 mL) added) and filtration to give **6** (295 mg, 81%) as a white solid. R_f 0.31 (toluene/acetone 10:1). $[\alpha]_D^{20}$ = +52.5 (c = 0.33, CH_2Cl_2). ^{1}H NMR (800 MHz; $CDCl_3$) δ 8.10-8.09 (m, 2H, Bz), 8.02-8.01 (m, 2H, Bz), 7.94-7.91 (m, 16H, Bz), 7.55-7.02 (m, 180H, Ph), 5.58-5.53 (m, 9H, $H^{CEGIKMOQS}$-1), 5.31 (t, J = 9.6 Hz, 1H, H^T-4), 5.19-5.14 (m, 9H, $H^{CEGIKMOQS}$-2), 5.09-5.08 (m, 1H, H^A-2), 5.05-5.04 (m, 1H, H^A-1), 4.95-4.88 (m, 10H, $H^{BDFHJLNPRT}$-1), 4.83-4.34 (m, 70H, $H^{ACEGIKMOQS}$-5, 30xCH_2Ph), 4.26-4.16 (m, 9H, $H^{CEGIKMOQS}$-3), 4.06-3.18 (m, 100H, H^A-3, $H^{ACEGIKMOQS}$-4, $H^{BDFHJLNPRT}$-2, $H^{BDFHJLNPRT}$-3, $H^{BDFHJLNPR}$-4, $H^{BDFHJLNPRT}$-5, $H^{BDFHJLNPRT}$-6$_{ab}$, 10xCOOCH_3), 3.24 (s, 3H, OCH_3). ^{13}C NMR (201 MHz; $CDCl_3$): δ 169.6, 169.4, 165.7, 165.2, 160.3, 138.0, 137.9, 137.5, 137.3, 133.7, 133.5, 130.0, 129.9, 129.4, 128.8, 128.5, 128.1, 128.0, 127.9, 127.7, 127.6, 127.4, 127.2, 99.3, 98.4, 98.1, 78.1, 77.0, 76.1, 76.0, 75.6, 75.5, 75.0, 74.8, 74.5, 74.3, 73.9, 73.8, 72.7, 72.6, 71.4, 70.8, 70.7, 70.6, 69.8, 69.3, 67.4, 63.5, 63.1, 52.1, 52.0, 51.7. MALDI TOF MS: monoisotopic m/z: calcd for $C_{413}H_{413}Cl_3N_{30}NaO_{112}$ $[M+Na]^+$: 7711.7; found: 7711.8.

Methyl (2-azido-3,6-di-*O*-benzyl-2-deoxy-α-D-glucopyranosyl)-(1→4)-(methyl 2-*O*-benzoyl-3-*O*-benzyl-α-L-idopyranosyl uronate)-(1→4)-2-azido-3,6-di-*O*-benzyl-2-deoxy-α-D-glucopyranosyl-(1→4)-(methyl 2-*O*-benzoyl-3-*O*-benzyl-α-L-idopyranosyl uronate)-(1→4)-2-azido-3,6-di-*O*-benzyl-2-deoxy-α-D-glucopyranosyl-(1→4)-(methyl 2-*O*-benzoyl-3-*O*-benzyl-α-L-idopyranosyl uronate)-(1→4)-2-azido-3,6-di-*O*-benzyl-2-deoxy-α-D-glucopyranosyl-(1→4)- (methyl 2-*O*-benzoyl-3-*O*-benzyl-α-L-idopyranosyl uronate)-(1→4)-2-azido-3,6-di-*O*-benzyl-2-deoxy-α-D-glucopyranosyl-(1→4)-(methyl 2-*O*-benzoyl-3-*O*-benzyl-α-L-idopyranosyl uronate)-(1→4)-2-azido-3,6-di-*O*-benzyl-2-deoxy-α-D-glucopyranosyl-(1→4)-(methyl 2-*O*-benzoyl-3-*O*-benzyl-α-L-idopyranosyl uronate)-(1→4)-2-azido-3,6-di-*O*-benzyl-2-deoxy-α-D-glucopyranosyl-(1→4)-(methyl 2-*O*-benzoyl-3-*O*-benzyl-α-L-idopyranosyl uronate)-(1→4)-2-azido-3,6-di-*O*-benzyl-2-deoxy-α-D-glucopyranosyl-(1→4)-(methyl 2-*O*-benzoyl-3-*O*-benzyl-α-L-idopyranosyl uronate)-(1→4)-2-azido-3,6-di-*O*-benzyl-2-deoxy-α-D-glucopyranosyl-(1→4)-(methyl 2-*O*-benzoyl-3-*O*-benzyl-α-L-idopyranosyl uronate)-(1→4)-2-azido-3,6-di-*O*-benzyl-2-deoxy-α-D-glucopyranosyl-(1→4)-(methyl 2-*O*-benzoyl-3-*O*-benzyl-α-L-idopyranoside) uronate, 7

The icosasaccharide **6** (278 mg, 0.036 mmol) was dissolved in a mixture of MeOH/pyridine (5 mL/ 2.5 mL) and heated to 60°C for 5 h. The solvents were evaporated and co-evaporated with toluene (2x10 mL). The crude product was purified using flash column chromatography (toluene/acetone

15:1) followed by precipitation (dissolved in EtOAc (5 mL) and hexane (5 mL) added) and filtration to yield **7** (264 mg, 97%) as a white solid. R_f 0.18 (toluene/acetone 10:1). $[\alpha]_D^{20}$ = +59.7 (c = 0.29, CH_2Cl_2). ^1H NMR (400 MHz; $CDCl_3$) δ 8.11-8.09 (m, 2H, Bz), 7.99-7.92 (m, 18H, Bz), 7.55-7.01 (m, 180H, Ph), 5.60-5.55 (m, 9H, $H^{CEGIKMOQS}$-1), 5.23-5.16 (m, 9H, $H^{CEGIKMOQS}$-2), 5.08-5.07 (m, 1H, H^A-2), 5.04-5.03 (m, 1H, H^A-1), 4.99-4.87 (m, 10H, $H^{BDFHJLNPRT}$-1), 4.83-4.34 (m, 70H, $H^{ACEGIKMOQS}$-5, 30xCH_2Ph), 4.24-4.15 (m, 9H, $H^{CEGIKMOQS}$-3), 4.06-3.18 (m, 101H, H^A-3, $H^{ACEGIKMOQS}$-4, $H^{BDFHJLNPRT}$-2, $H^{BDFHJLNPRT}$-3, $H^{BDFHJLNPRT}$-4, $H^{BDFHJLNPRT}$-5, $H^{BDFHJLNPRT}$-6_{ab}, 10xCOOCH_3), 3.24 (s, 3H, OCH_3), 2.66 (broad s, 1H, OH). MALDI TOF MS: monoisotopic m/z: calcd for $C_{411}H_{414}N_{30}NaO_{111}$ $[M+Na]^+$: 7567.8; found: 7567.9.

Methyl (2-azido-3,6-di-*O*-benzyl-2-deoxy-4-*O*-trichloroacetyl-α-D-glucopyranosyl)-(1→4)-(methyl 2-*O*-benzoyl-3-*O*-benzyl-α-L-idopyranosyl uronate)-(1→4)-2-azido-3,6-di-*O*-benzyl-2-deoxy-α-D-glucopyranosyl-(1→4)-(methyl 2-*O*-benzoyl-3-*O*-benzyl-α-L-idopyranosyl uronate)-(1→4)-2-azido-3,6-di-*O*-benzyl-2-deoxy-α-D-glucopyranosyl-(1→4)-(methyl 2-*O*-benzoyl-3-*O*-benzyl-α-L-idopyranosyl uronate)-(1→4)-2-azido-3,6-di-*O*-benzyl-2-deoxy-α-D-glucopyranosyl-(1→4)- (methyl 2-*O*-benzoyl-3-*O*-benzyl-α-L-idopyranosyl uronate)-(1→4)-2-azido-3,6-di-*O*-benzyl-2-deoxy-α-D-glucopyranosyl-(1→4)-(methyl 2-*O*-benzoyl-3-*O*-benzyl-α-L-idopyranosyl uronate)-(1→4)-2-azido-3,6-di-*O*-benzyl-2-deoxy-α-D-glucopyranosyl-(1→4)-(methyl 2-*O*-benzoyl-3-*O*-benzyl-α-L-idopyranosyl uronate)-(1→4)-2-azido-3,6-di-*O*-benzyl-2-deoxy-α-D-glucopyranosyl-(1→4)-(methyl 2-*O*-benzoyl-3-*O*-benzyl-α-L-idopyranosyl uronate)-(1→4)-2-azido-3,6-di-*O*-benzyl-2-deoxy-α-D-glucopyranosyl-(1→4)-(methyl 2-*O*-benzoyl-3-*O*-benzyl-α-L-idopyranosyl uronate)-(1→4)-2-azido-3,6-di-*O*-benzyl-2-deoxy-α-D-glucopyranosyl-(1→4)-(methyl 2-*O*-benzoyl-3-*O*-benzyl-α-L-idopyranosyl uronate)-(1→4)-2-azido-3,6-di-*O*-benzyl-2-deoxy-α-D-glucopyranosyl-(1→4)-(methyl 2-*O*-benzoyl-3-*O*-benzyl-α-L-idopyranosyl uronate)-(1→4)-2-azido-3,6-di-*O*-benzyl-2-deoxy-α-D-glucopyranosyl-(1→4)-(methyl 2-*O*-benzoyl-3-*O*-benzyl-α-L-idopyranoside) uronate 8

Acceptor icosasaccharide **7** (254 mg, 0.034 mmol) and thioglycoside tetrasaccharide donor **1** (77 mg, 0.044 mmol) was dissolved in dry DCM (2 mL) under N_2. Freshly activated 4Å powdered molecular sieves (185 mg) was added and the solution cooled to 0 °C in an icebath. After 10 min. NIS (19 mg, 0.084 mmol) was added, and after another 10 min. AgOTf (catalytic amount) was added. The suspension changed colour from pale yellow to deep red, was stirred for 30 min. and the reaction was quenched into a separating funnel containing a mixture of DCM (30 mL), saturated aqueous $NaHCO_3$ (20 mL) and $Na_2S_2O_3$ (1 mL, 10% aqueous). After shaking until the iodine colour was removed the suspension was filtered through a short pad of Celite® washing with water and DCM. The layers were separated and the aqueous extracted with DCM (10 mL). The organic layers were combined, dried ($MgSO_4$) and solvent removed *in vacuo*. The crude was purified by silica gel flash column chromatography (toluene/acetone 20:1 to 15:1 to 10:1) followed by precipitation (dissolved in warm EtOAc (5 mL) and hexane (5 mL) added) and filtration to give **8** (239 mg, 77%) as a white solid. Also acceptor starting material (42 mg, 16%) was recovered. R_f 0.29 (toluene/acetone 10:1). $[\alpha]_D^{20}$ = +37.9 (c = 0.45, CH_2Cl_2). ^1H NMR (400 MHz; $CDCl_3$) δ 8.17-8.15 (m, 2H, Bz), 8.09-8.07 (m, 2H, Bz), 8.02-7.97 (m, 20H, Bz), 7.59-7.05 (m, 216H, Ph), 5.62-5.58 (m, 11H, $H^{CEGIKMOQSUW}$-1), 5.31 (t, J = 9.6 Hz, 1H, H^X-4), 5.26-5.22 (m, 9H, $H^{CEGIKMOQSUW}$-2), 5.14-5.13 (m, 1H, H^A-2), 5.10-5.09 (m, 1H, H^A-1), 4.99-4.92 (m, 12H, $H^{BDFHJLNPRTVX}$-1), 4.88-4.36 (m, 84H, $H^{ACEGIKMOQSUW}$-5, 36xCH_2Ph), 4.30-4.19 (m, 11H, $H^{CEGIKMOQSUW}$-3), 4.12-3.20 (m, 120H,

H^{A}-3, $H^{ACEGIKMOQSUW}$-4, $H^{BDFHJLNPRTVX}$-2, $H^{BDFHJLNPRTVX}$-3, $H^{BDFHJLNPRTV}$-4, $H^{BDFHJLNPRTVX}$-5, $H^{BDFHJLNPRTVX}$-6_{ab}, 12xCOOC*H*$_3$), 3.24 (s, 3H, OC*H*$_3$). MALDI TOF MS: m/z: calcd for $C_{495}H_{495}Cl_3N_{36}NaO_{134}$ [*M*+Na]$^+$: 9221; found: 9221.

Methyl (2-azido-3,6-di-*O*-benzyl-2-deoxy-α-D-glucopyranosyl)-(1⟶4)-(methyl 2-*O*-benzoyl-3-*O*-benzyl-α-L-idopyranosyl uronate)-(1⟶4)-2-azido-3,6-di-*O*-benzyl-2-deoxy-α-D-glucopyranosyl-(1⟶4)-(methyl 2-*O*-benzoyl-3-*O*-benzyl-α-L-idopyranosyl uronate)-(1⟶4)-2-azido-3,6-di-*O*-benzyl-2-deoxy-α-D-glucopyranosyl-(1⟶4)-(methyl 2-*O*-benzoyl-3-*O*-benzyl-α-L-idopyranosyl uronate)-(1⟶4)-2-azido-3,6-di-*O*-benzyl-2-deoxy-α-D-glucopyranosyl-(1⟶4)- (methyl 2-*O*-benzoyl-3-*O*-benzyl-α-L-idopyranosyl uronate)-(1⟶4)-2-azido-3,6-di-*O*-benzyl-2-deoxy-α-D-glucopyranosyl-(1⟶4)-(methyl 2-*O*-benzoyl-3-*O*-benzyl-α-L-idopyranosyl uronate)-(1⟶4)-2-azido-3,6-di-*O*-benzyl-2-deoxy-α-D-glucopyranosyl-(1⟶4)-(methyl 2-*O*-benzoyl-3-*O*-benzyl-α-L-idopyranosyl uronate)-(1⟶4)-2-azido-3,6-di-*O*-benzyl-2-deoxy-α-D-glucopyranosyl-(1⟶4)-(methyl 2-*O*-benzoyl-3-*O*-benzyl-α-L-idopyranosyl uronate)-(1⟶4)-2-azido-3,6-di-*O*-benzyl-2-deoxy-α-D-glucopyranosyl-(1⟶4)-(methyl 2-*O*-benzoyl-3-*O*-benzyl-α-L-idopyranosyl uronate)-(1⟶4)-2-azido-3,6-di-*O*-benzyl-2-deoxy-α-D-glucopyranosyl-(1⟶4)-(methyl 2-*O*-benzoyl-3-*O*-benzyl-α-L-idopyranosyl uronate)-(1⟶4)-2-azido-3,6-di-*O*-benzyl-2-deoxy-α-D-glucopyranosyl-(1⟶4)-(methyl 2-*O*-benzoyl-3-*O*-benzyl-α-L-idopyranosyl uronate)-(1⟶4)-2-azido-3,6-di-*O*-benzyl-2-deoxy-α-D-glucopyranosyl-(1⟶4)-(methyl 2-*O*-benzoyl-3-*O*-benzyl-α-L-idopyranosyl uronate)-(1⟶4)-2-azido-3,6-di-*O*-benzyl-2-deoxy-α-D-glucopyranosyl-(1⟶4)-(methyl 2-*O*-benzoyl-3-*O*-benzyl-α-L-idopyranoside) uronate, 9

The tetracosasaccharide **8** (186 mg, 0.020 mmol) was dissolved in a mixture of MeOH/pyridine (3 mL/2 mL) and heated to 60 °C for 6 h. The solvents were evaporated and co-evaporated with toluene (2x10 mL). The crude product was purified using flash column chromatography (toluene/ acetone 15:1 to 10:1) followed by precipitation (dissolved in warm EtOAc (5 mL) and hexane (5 mL) added) and filtration to yield **9** (173 mg, 95%) as a white solid. R_f 0.18 (toluene/acetone 10:1). $[\alpha]_D^{20}$ = +44.0 (*c* = 0.36, CH_2Cl_2). ^{1}H NMR (400 MHz; $CDCl_3$) δ 8.10-8.08 (m, 2H, Bz), 7.99-7.90 (m, 22H, Bz), 7.56-7.01 (m, 216H, Ph), 5.60-5.54 (m, 11H, $H^{CEGIKMOQSUW}$-1), 5.24-5.16 (m, 11H, $H^{CEGIKMOQSUW}$-2), 5.08-5.07 (m, 1H, H^{A}-2), 5.04-5.03 (m, 1H, H^{A}-1), 4.99-4.87 (m, 12H, $H^{BDFHJLNPRTVX}$-1), 4.84-4.34 (m, 84H, $H^{ACEGIKMOQSUW}$-5, 36xC*H*$_2$Ph), 4.26-4.16 (m, 11H, $H^{CEGIKMOQSUW}$-3), 4.06-3.18 (m, 121H, H^{A}-3, $H^{ACEGIKMOQSUW}$-4, $H^{BDFHJLNPRTVX}$-2, $H^{BDFHJLNPRTVX}$-3, $H^{BDFHJLNPRTVX}$-4, $H^{BDFHJLNPRTVX}$-5, $H^{BDFHJLNPRTVX}$-6_{ab}, 12xCOOC*H*$_3$), 3.23 (s, 3H, OC*H*$_3$), 2.56 (d, *J* = 2.5 Hz, 1H, O*H*). MALDI TOF MS: m/z: calcd for $C_{493}H_{496}N_{36}NaO_{133}$ [*M*+Na]$^+$: 9076; found: 9076.

Methyl (2-azido-3,6-di-*O*-benzyl-2-deoxy-4-*O*-trichloroacetyl-α-D-glucopyranosyl)-(1⟶4)-(methyl 2-*O*-benzoyl-3-*O*-benzyl-α-L-idopyranosyl uronate)-(1⟶4)-2-azido-3,6-di-*O*-benzyl-2-deoxy-α-D-glucopyranosyl-(1⟶4)-(methyl 2-*O*-benzoyl-3-*O*-benzyl-α-L-idopyranosyl uronate)-(1⟶4)-2-azido-3,6-di-*O*-benzyl-2-deoxy-α-D-glucopyranosyl-(1⟶4)-(methyl 2-*O*-benzoyl-3-*O*-benzyl-α-L-idopyranosyl uronate)-(1⟶4)-2-azido-3,6-di-*O*-benzyl-2-deoxy-α-D-glucopyranosyl-(1⟶4)- (methyl 2-*O*-benzoyl-3-*O*-benzyl-α-L-idopyranosyl uronate)-(1⟶4)-2-azido-3,6-di-*O*-benzyl-2-deoxy-α-D-glucopyranosyl-(1⟶4)-(methyl 2-*O*-benzoyl-3-*O*-benzyl-α-L-idopyranosyl uronate)-(1⟶4)-2-azido-3,6-di-*O*-benzyl-2-deoxy-α-D-glucopyranosyl-(1⟶4)-(methyl 2-*O*-benzoyl-3-*O*-benzyl-α-L-idopyranosyl uronate)-(1⟶4)-2-azido-3,6-di-*O*-benzyl-2-deoxy-α-D-glucopyranosyl-(1⟶4)-

(methyl 2-*O*-benzoyl-3-*O*-benzyl-α-L-idopyranosyl uronate)-(1→4)-2-azido-3,6-di-*O*-benzyl-2-deoxy-α-D-glucopyranosyl-(1→4)-(methyl 2-*O*-benzoyl-3-*O*-benzyl-α-L-idopyranosyl uronate)-(1→4)-2-azido-3,6-di-*O*-benzyl-2-deoxy-α-D-glucopyranosyl-(1→4)-(methyl 2-*O*-benzoyl-3-*O*-benzyl-α-L-idopyranosyl uronate)-(1→4)-2-azido-3,6-di-*O*-benzyl-2-deoxy-α-D-glucopyranosyl-(1→4)-(methyl 2-*O*-benzoyl-3-*O*-benzyl-α-L-idopyranosyl uronate)-(1→4)-2-azido-3,6-di-*O*-benzyl-2-deoxy-α-D-glucopyranosyl-(1→4)-(methyl 2-*O*-benzoyl-3-*O*-benzyl-α-L-idopyranosyl uronate)-(1→4)-2-azido-3,6-di-*O*-benzyl-2-deoxy-α-D-glucopyranosyl-(1→4)-(methyl 2-*O*-benzoyl-3-*O*-benzyl-α-L-idopyranosyl uronate)-(1→4)-2-azido-3,6-di-*O*-benzyl-2-deoxy-α-D-glucopyranosyl-(1→4)-(methyl 2-*O*-benzoyl-3-*O*-benzyl-α-L-idopyranosyl uronate)-(1→4)-2-azido-3,6-di-*O*-benzyl-2-deoxy-α-D-glucopyranosyl-(1→4)-(methyl 2-*O*-benzoyl-3-*O*-benzyl-α-L-idopyranoside) uronate, 10

Acceptor tetracosasaccharide **9** (161 mg, 0.018 mmol) and thioglycoside tetrasaccharide donor **1** (41 mg, 0.023 mmol) was dissolved in dry DCM (1.5 mL) under N_2. Freshly activated 4Å powdered molecular sieves (174 mg) was added and the solution cooled to 0 °C in an icebath. After 10 min. NIS (15 mg, 0.067 mmol) was added, and after another 10 min. AgOTf (catalytic amount) was added. The suspension changed colour from pale yellow to deep red, was stirred for 30 min. and the reaction was quenched into a separating funnel containing a mixture of DCM (20 mL), saturated aqueous $NaHCO_3$ (10 mL) and $Na_2S_2O_3$ (1 mL, 10% aqueous). After shaking until the iodine colour was removed the suspension was filtered through a short pad of Celite® washing with water and DCM. The layers were separated and the aqueous extracted with DCM (10 mL). The organic layers were combined, dried ($MgSO_4$) and solvent removed *in vacuo*. The crude was purified by silica gel flash column chromatography (toluene/acetone 20:1 to 15:1 to 10:1) followed by precipitation (dissolved in warm EtOAc (5 mL) and hexane (5 mL) added) and filtration to give **10 (**143 mg, 75%) as a white solid. Also acceptor starting material (35 mg, 21%) was recovered. R_f 0.29 (toluene/acetone 10:1). $[\alpha]_D^{20}$ = +40.7 (*c* = 0.36, CH_2Cl_2). ^1H NMR (400 MHz; $CDCl_3$) δ 8.13-8.11 (m, 2H, Bz), 8.05-8.03 (m, 2H, Bz), 7.98-7.93 (m, 24H, Bz), 7.57-7.05 (m, 252H, Ph), 5.59-5.55 (m, 13H, $H^{(BCDEFGHIJKLMN)ido}$-1), 5.34 (t, *J* = 9.6 Hz, 1H, H^{Nglcn}-4), 5.26-5.22 (m, 13H, $H^{(BCDEFGHIJKLMN)ido}$-2), 5.10-5.09 (m, 1H, H^{Aido}-2), 5.07-5.06 (m, 1H, H^{Aido}-1), 4.99-4.88 (m, 14H, $H^{(ABCDEFGHIJKLMN)glcn}$-1), 4.86-4.33 (m, 98H, $H^{(ABCDEFGHIJKLMN)ido}$-5, 42xCH_2Ph), 4.27-4.18 (m, 13H, $H^{(BCDEFGHIJKLMN)ido}$-3), 4.12-3.20 (m, 140H, H^{Aido}-3, $H^{(ABCDEFGHIJKLMN)ido}$ -4, $H^{(ABCDEFGHIJKLMN)glcn}$-2, $H^{(ABCDEFGHIJKLMN)glcn}$-3, $H^{(ABCDEFGHIJKLM)glcn}$ -4, $H^{(ABCDEFGHIJKLMN)glcn}$-5, $H^{(ABCDEFGHIJKLMN)glcn}$-6_{ab}, 14xCOOCH_3), 3.25 (s, 3H, OCH_3). MALDI TOF MS: m/z: calcd for $C_{577}H_{577}Cl_3N_{42}NaO_{156}$ [*M* +Na]$^+$: 10725; found: 10726.

Methyl (2-azido-3,6-di-*O*-benzyl-2-deoxy-α-D-glucopyranosyl)-(1→4)-(methyl 2-*O*-benzoyl-3-*O*-benzyl-α-L-idopyranosyl uronate)-(1→4)-2-azido-3,6-di-*O*-benzyl-2-deoxy-α-D-glucopyranosyl-(1→4)-(methyl 2-*O*-benzoyl-3-*O*-benzyl-α-L-idopyranosyl uronate)-(1→4)-2-azido-3,6-di-*O*-benzyl-2-deoxy-α-D-glucopyranosyl-(1→4)-(methyl 2-*O*-benzoyl-3-*O*-benzyl-α-L-idopyranosyl uronate)-(1→4)-2-azido-3,6-di-*O*-benzyl-2-deoxy-α-D-glucopyranosyl-(1→4)- (methyl 2-*O*-benzoyl-3-*O*-benzyl-α-L-idopyranosyl uronate)-(1→4)-2-azido-3,6-di-*O*-benzyl-2-deoxy-α-D-glucopyranosyl-(1→4)-(methyl 2-*O*-benzoyl-3-*O*-benzyl-α-L-idopyranosyl uronate)-(1→4)-2-azido-3,6-di-*O*-benzyl-2-deoxy-α-D-glucopyranosyl-(1→4)-(methyl 2-*O*-benzoyl-3-*O*-benzyl-α-L-idopyranosyl uronate)-(1→4)-2-azido-3,6-di-*O*-benzyl-2-deoxy-α-D-glucopyranosyl-(1→4)-(methyl 2-*O*-benzoyl-3-*O*-benzyl-α-L-idopyranosyl uronate)-(1→4)-2-azido-3,6-di-*O*-benzyl-2-deoxy-α-D-glucopyranosyl-(1→4)-

(methyl 2-*O*-benzoyl-3-*O*-benzyl-α-L-idopyranosyl uronate)-(1→4)-2-azido-3,6-di-*O*-benzyl-2-deoxy-α-D-glucopyranosyl-(1→4)-(methyl 2-*O*-benzoyl-3-*O*-benzyl-α-L-idopyranosyl uronate)-(1→4)-2-azido-3,6-di-*O*-benzyl-2-deoxy-α-D-glucopyranosyl-(1→4)-(methyl 2-*O*-benzoyl-3-*O*-benzyl-α-L-idopyranosyl uronate)-(1→4)-2-azido-3,6-di-*O*-benzyl-2-deoxy-α-D-glucopyranosyl-(1→4)-(methyl 2-*O*-benzoyl-3-*O*-benzyl-α-L-idopyranosyl uronate)-(1→4)-2-azido-3,6-di-*O*-benzyl-2-deoxy-α-D-glucopyranosyl-(1→4)-(methyl 2-*O*-benzoyl-3-*O*-benzyl-α-L-idopyranosyl uronate)-(1→4)-2-azido-3,6-di-*O*-benzyl-2-deoxy-α-D-glucopyranosyl-(1→4)-(methyl 2-*O*-benzoyl-3-*O*-benzyl-α-L-idopyranosyl uronate)-(1→4)-2-azido-3,6-di-*O*-benzyl-2-deoxy-α-D-glucopyranosyl-(1→4)-(methyl 2-*O*-benzoyl-3-*O*-benzyl-α-L-idopyranoside) uronate, 11

The octacosasaccharide **10** (129 mg, 0.012 mmol) was dissolved in a mixture of MeOH/pyridine (2 mL/2.5 mL) and heated to 60 °C for 4 h. The solvents were evaporated and co-evaporated with toluene (2x5 mL). The crude product was purified using flash column chromatography (toluene/ acetone 15:1 to 10:1) followed by precipitation (dissolved in warm EtOAc (5 mL) and hexane (5 mL) added) and filtration to yield **11** (118 mg, 93%) as a white solid. R_f 0.18 (toluene/acetone 10:1). $[\alpha]_D^{20}$ = +38.7 (*c* = 0.34, CH_2Cl_2). ^{1}H NMR (400 MHz; $CDCl_3$) δ 8.12-8.10 (m, 2H, Bz), 8.00-7.98 (m, 2H, Bz), 7.96-7.92 (m, 24H, Bz), 7.57-7.05 (m, 252H, Ph), 5.60-5.53 (m, 13H, $H^{(BCDEFGHIJKLMN)ido}$-1), 5.23-5.17 (m, 13H, $H^{(BCDEFGHIJKLMN)ido}$-2), 5.09-5.08 (m, 1H, H^{Aido}-2), 5.06-5.05 (m, 1H, H^{Aido}-1), 5.01-4.88 (m, 14H, $H^{(ABCDEFGHIJKLMN)glcn}$-1), 4.85-4.32 (m, 98H, $H^{(ABCDEFGHIJKLMN)ido}$-5, 42xCH_2Ph), 4.27-4.16 (m, 13H, $H^{(BCDEFGHIJKLMN)ido}$-3), 4.12-3.20 (m, 141H, H^{Aido}-3, $H^{(ABCDEFGHIJKLMN)ido}$ -4, $H^{(ABCDEFGHIJKLMN)glcn}$-2, $H^{(ABCDEFGHIJKLMN)glcn}$-3, $H^{(ABCDEFGHIJKLMN)glcn}$ -4, $H^{(ABCDEFGHIJKLMN)glcn}$-5, $H^{(ABCDEFGHIJKLMN)glcn}$-6_{ab}, 14xCOOCH_3), 3.22 (s, 3H, OCH_3), 2.60 (d, *J* = 2.4 Hz, 1H, O*H*). MALDI TOF MS: m/z: calcd for $C_{575}H_{578}N_{42}NaO_{155}$ $[M+Na]^+$: 10579; found: 10579.

Methyl (2-azido-3,6-di-*O*-benzyl-2-deoxy-4-*O*-trichloroacetyl-α-D-glucopyranosyl)-(1→4)-(methyl 2-*O*-benzoyl-3-*O*-benzyl-α-L-idopyranosyl uronate)-(1→4)-2-azido-3,6-di-*O*-benzyl-2-deoxy-α-D-glucopyranosyl-(1→4)-(methyl 2-*O*-benzoyl-3-*O*-benzyl-α-L-idopyranosyl uronate)-(1→4)-2-azido-3,6-di-*O*-benzyl-2-deoxy-α-D-glucopyranosyl-(1→4)-(methyl 2-*O*-benzoyl-3-*O*-benzyl-α-L-idopyranosyl uronate)-(1→4)-2-azido-3,6-di-*O*-benzyl-2-deoxy-α-D-glucopyranosyl-(1→4)- (methyl 2-*O*-benzoyl-3-*O*-benzyl-α-L-idopyranosyl uronate)-(1→4)-2-azido-3,6-di-*O*-benzyl-2-deoxy-α-D-glucopyranosyl-(1→4)-(methyl 2-*O*-benzoyl-3-*O*-benzyl-α-L-idopyranosyl uronate)-(1→4)-2-azido-3,6-di-*O*-benzyl-2-deoxy-α-D-glucopyranosyl-(1→4)-(methyl 2-*O*-benzoyl-3-*O*-benzyl-α-L-idopyranosyl uronate)-(1→4)-2-azido-3,6-di-*O*-benzyl-2-deoxy-α-D-glucopyranosyl-(1→4)-(methyl 2-*O*-benzoyl-3-*O*-benzyl-α-L-idopyranosyl uronate)-(1→4)-2-azido-3,6-di-*O*-benzyl-2-deoxy-α-D-glucopyranosyl-(1→4)-(methyl 2-*O*-benzoyl-3-*O*-benzyl-α-L-idopyranosyl uronate)-(1→4)-2-azido-3,6-di-*O*-benzyl-2-deoxy-α-D-glucopyranosyl-(1→4)-(methyl 2-*O*-benzoyl-3-*O*-benzyl-α-L-idopyranosyl uronate)-(1→4)-2-azido-3,6-di-*O*-benzyl-2-deoxy-α-D-glucopyranosyl-(1→4)-(methyl 2-*O*-benzoyl-3-*O*-benzyl-α-L-idopyranosyl uronate)-(1→4)-2-azido-3,6-di-*O*-benzyl-2-deoxy-α-D-glucopyranosyl-(1→4)-(methyl 2-*O*-benzoyl-3-*O*-benzyl-α-L-

idopyranosyl uronate)-(1→4)-2-azido-3,6-di-*O*-benzyl-2-deoxy-α-D-glucopyranosyl-(1→4)-(methyl 2-*O*-benzoyl-3-*O*-benzyl-α-L-idopyranosyl uronate)-(1→4)-2-azido-3,6-di-*O*-benzyl-2-deoxy-α-D-glucopyranosyl-(1→4)-(methyl 2-*O*-benzoyl-3-*O*-benzyl-α-L-idopyranoside) uronate, 12

Acceptor octacosasaccharide **11** (147 mg, 0.014 mmol) and thioglycoside tetrasaccharide donor **1** (32 mg, 0.018 mmol) was dissolved in dry DCM (1.5 mL) under N_2. Freshly activated 4Å powdered molecular sieves (125 mg) was added and the solution cooled to 0 °C in an icebath. After 10 min. NIS (10 mg, 0.044 mmol) was added, and after another 10 min. AgOTf (catalytic amount) was added. The suspension changed colour from pale yellow to deep red, was stirred for 30 min. and the reaction was quenched into a separating funnel containing a mixture of DCM (20 mL), saturated aqueous $NaHCO_3$ (10 mL) and $Na_2S_2O_3$ (1 mL, 10% aqueous). After shaking until the iodine colour was removed the suspension was filtered through a short pad of Celite® washing with water and DCM. The layers were separated and the aqueous extracted with DCM (5 mL). The organic layers were combined, dried ($MgSO_4$) and solvent removed *in vacuo*. The crude was purified by silica gel flash column chromatography (toluene/acetone 20:1 to 15:1 to 10:1) followed by precipitation (dissolved in warm EtOAc (4 mL) and hexane (4 mL) added) and filtration to give **12** (132 mg, 78%) as a white solid. Also acceptor starting material (28 mg, 19%) was recovered. R_f 0.28 (toluene/acetone 10:1). $[\alpha]_D^{20}$ = +41.9 (*c* = 0.33, CH_2Cl_2). ^{1}H NMR (400 MHz; $CDCl_3$) δ 8.18-8.16 (m, 2H, Bz), 8.10-8.08 (m, 2H, Bz), 8.03-7.97 (m, 28H, Bz), 7.63-7.10 (m, 288H, Ph), 5.65-5.60 (m, 15H, $H^{(BCDEFGHIJKLMNOP)ido}$-1), 5.39 (t, *J* = 9.6 Hz, 1H, H^{Pglcn}-4), 5.27-5.23 (m, 15H, $H^{(BCDEFGHIJKLMNOP)ido}$-2), 5.15-5.14 (m, 1H, H^{Aido}-2), 5.12-5.11 (m, 1H, H^{Aido}-1), 5.02-4.94 (m, 16H, $H^{(ABCDEFGHIJKLMNOP)glcn}$-1), 4.92-4.38 (m, 112H, $H^{(ABCDEFGHIJKLMNOP)ido}$-5, 48x$CH_2$Ph), 4.32-4.22 (m, 15H, $H^{(BCDEFGHIJKLMNOP)ido}$-3), 4.14-3.22 (m, 160H, H^{Aido}-3, $H^{(ABCDEFGHIJKLMNOP)ido}$ -4, $H^{(ABCDEFGHIJKLMNOP)glcn}$-2, $H^{(ABCDEFGHIJKLMNOP)glcn}$-3, $H^{(ABCDEFGHIJKLMO)glcn}$ -4, $H^{(ABCDEFGHIJKLMNOP)glcn}$-5, $H^{(ABCDEFGHIJKLMNOP)glcn}$-$6_{ab}$, 16xCOO$CH_3$), 3.28 (s, 3H, O$CH_3$). MALDI TOF MS: m/z: calcd for $C_{659}H_{659}Cl_3N_{48}NaO_{178}$ $[M+Na]^+$: 12229; found: 12230.

Methyl (2-azido-3,6-di-*O*-benzyl-2-deoxy-α-D-glucopyranosyl)-(1→4)-(methyl 2-*O*-benzoyl-3-*O*-benzyl-α-L-idopyranosyl uronate)-(1→4)-2-azido-3,6-di-*O*-benzyl-2-deoxy-α-D-glucopyranosyl-(1→4)-(methyl 2-*O*-benzoyl-3-*O*-benzyl-α-L-idopyranosyl uronate)-(1→4)-2-azido-3,6-di-*O*-benzyl-2-deoxy-α-D-glucopyranosyl-(1→4)-(methyl 2-*O*-benzoyl-3-*O*-benzyl-α-L-idopyranosyl uronate)-(1→4)-2-azido-3,6-di-*O*-benzyl-2-deoxy-α-D-glucopyranosyl-(1→4)- (methyl 2-*O*-benzoyl-3-*O*-benzyl-α-L-idopyranosyl uronate)-(1→4)-2-azido-3,6-di-*O*-benzyl-2-deoxy-α-D-glucopyranosyl-(1→4)-(methyl 2-*O*-benzoyl-3-*O*-benzyl-α-L-idopyranosyl uronate)-(1→4)-2-azido-3,6-di-*O*-benzyl-2-deoxy-α-D-glucopyranosyl-(1→4)-(methyl 2-*O*-benzoyl-3-*O*-benzyl-α-L-idopyranosyl uronate)-(1→4)-2-azido-3,6-di-*O*-benzyl-2-deoxy-α-D-glucopyranosyl-(1→4)-(methyl 2-*O*-benzoyl-3-*O*-benzyl-α-L-idopyranosyl uronate)-(1→4)-2-azido-3,6-di-*O*-benzyl-2-deoxy-α-D-glucopyranosyl-(1→4)-(methyl 2-*O*-benzoyl-3-*O*-benzyl-α-L-idopyranosyl uronate)-(1→4)-2-azido-3,6-di-*O*-benzyl-2-deoxy-α-D-glucopyranosyl-(1→4)-(methyl 2-*O*-benzoyl-3-*O*-benzyl-α-L-idopyranosyl uronate)-(1→4)-2-azido-3,6-di-*O*-benzyl-2-deoxy-α-D-glucopyranosyl-(1→4)-(methyl 2-*O*-benzoyl-3-*O*-benzyl-α-L-idopyranosyl uronate)-(1→4)-2-azido-3,6-di-*O*-benzyl-2-deoxy-α-D-glucopyranosyl-(1→4)-(methyl 2-*O*-benzoyl-3-*O*-benzyl-α-L-

idopyranosyl uronate)-(1→4)-2-azido-3,6-di-*O*-benzyl-2-deoxy-α-D-glucopyranosyl-(1→4)-(methyl 2-*O*-benzoyl-3-*O*-benzyl-α-L-idopyranosyl uronate)-(1→4)-2-azido-3,6-di-*O*-benzyl-2-deoxy-α-D-glucopyranosyl-(1→4)-(methyl 2-*O*-benzoyl-3-*O*-benzyl-α-L-idopyranosyl uronate)-(1→4)-2-azido-3,6-di-*O*-benzyl-2-deoxy-α-D-glucopyranosyl-(1→4)-(methyl 2-*O*-benzoyl-3-*O*-benzyl-α-L-idopyranoside) uronate, 13

The dotriacontasaccharide **12** (117 mg, 0.010 mmol) was dissolved in a mixture of MeOH/pyridine (2 mL/3 mL) and heated to 50 °C for 5 h. The solvents were evaporated and co-evaporated with toluene (2x5 mL). The crude product was purified using flash column chromatography (toluene/ acetone 15:1) followed by precipitation (dissolved in warm EtOAc (4 mL) and hexane (4 mL) added) and filtration to yield **13** (109 mg, 95%) as a white solid. R_f 0.18 (toluene/acetone 10:1). $[\alpha]_D^{20}$ = +38.3 (*c* = 0.16, CH_2Cl_2). ^{1}H NMR (400 MHz; $CDCl_3$) δ 8.18-8.16 (m, 2H, Bz), 8.06-8.04 (m, 2H, Bz), 8.03-7.97 (m, 28H, Bz), 7.62-7.10 (m, 288H, Ph), 5.67-5.60 (m, 15H, $H^{(BCDEFGHIJKLMNOP)ido}$-1), 5.27-5.23 (m, 15H, $H^{(BCDEFGHIJKLMNOP)ido}$-2), 5.15-5.14 (m, 1H, H^{Aido}-2), 5.11-5.10 (m, 1H, H^{Aido}-1), 5.06-4.94 (m, 16H, $H^{(ABCDEFGHIJKLMNOP)glcn}$-1), 4.92-4.38 (m, 112H, $H^{(ABCDEFGHIJKLMNOP)ido}$-5, 48xC$H_2$Ph), 4.32-4.22 (m, 15H, $H^{(BCDEFGHIJKLMNOP)ido}$-3), 4.14-3.22 (m, 161H, H^{Aido}-3, $H^{(ABCDEFGHIJKLMNOP)ido}$ -4, $H^{(ABCDEFGHIJKLMNOP)glcn}$-2, $H^{(ABCDEFGHIJKLMNOP)glcn}$-3, $H^{(ABCDEFGHIJKLMOP)glcn}$ -4, $H^{(ABCDEFGHIJKLMNOP)glcn}$-5, $H^{(ABCDEFGHIJKLMNOP)glcn}$-$6_{ab}$, 16xCOOC$H_3$), 3.28 (s, 3H, OC$H_3$), 2.68 (broad s, 1H, O*H*). MALDI TOF MS: m/z: calcd for $C_{657}H_{660}N_{48}NaO_{177}$ $[M+Na]^+$: 12083; found: 12085.

Methyl (2-azido-3,6-di-*O*-benzyl-2-deoxy-4-*O*-trichloroacetyl-α-D-glucopyranosyl)-(1→4)-(methyl 2-*O*-benzoyl-3-*O*-benzyl-α-L-idopyranosyl uronate)-(1→4)-2-azido-3,6-di-*O*-benzyl-2-deoxy-α-D-glucopyranosyl-(1→4)-(methyl 2-*O*-benzoyl-3-*O*-benzyl-α-L-idopyranosyl uronate)-(1→4)-2-azido-3,6-di-*O*-benzyl-2-deoxy-α-D-glucopyranosyl-(1→4)-(methyl 2-*O*-benzoyl-3-*O*-benzyl-α-L-idopyranosyl uronate)-(1→4)-2-azido-3,6-di-*O*-benzyl-2-deoxy-α-D-glucopyranosyl-(1→4)- (methyl 2-*O*-benzoyl-3-*O*-benzyl-α-L-idopyranosyl uronate)-(1→4)-2-azido-3,6-di-*O*-benzyl-2-deoxy-α-D-glucopyranosyl-(1→4)-(methyl 2-*O*-benzoyl-3-*O*-benzyl-α-L-idopyranosyl uronate)-(1→4)-2-azido-3,6-di-*O*-benzyl-2-deoxy-α-D-glucopyranosyl-(1→4)-(methyl 2-*O*-benzoyl-3-*O*-benzyl-α-L-idopyranosyl uronate)-(1→4)-2-azido-3,6-di-*O*-benzyl-2-deoxy-α-D-glucopyranosyl-(1→4)-(methyl 2-*O*-benzoyl-3-*O*-benzyl-α-L-idopyranosyl uronate)-(1→4)-2-azido-3,6-di-*O*-benzyl-2-deoxy-α-D-glucopyranosyl-(1→4)-(methyl 2-*O*-benzoyl-3-*O*-benzyl-α-L-idopyranosyl uronate)-(1→4)-2-azido-3,6-di-*O*-benzyl-2-deoxy-α-D-glucopyranosyl-(1→4)-(methyl 2-*O*-benzoyl-3-*O*-benzyl-α-L-idopyranosyl uronate)-(1→4)-2-azido-3,6-di-*O*-benzyl-2-deoxy-α-D-glucopyranosyl-(1→4)-(methyl 2-*O*-benzoyl-3-*O*-benzyl-α-L-idopyranosyl uronate)-(1→4)-2-azido-3,6-di-*O*-benzyl-2-deoxy-α-D-glucopyranosyl-(1→4)-(methyl 2-*O*-benzoyl-3-*O*-benzyl-α-L-idopyranosyl uronate)-(1→4)-2-azido-3,6-di-*O*-benzyl-2-deoxy-α-D-glucopyranosyl-(1→4)-(methyl 2-*O*-benzoyl-3-*O*-benzyl-α-L-idopyranosyl uronate)-(1→4)-2-azido-3,6-di-*O*-benzyl-2-deoxy-α-D-glucopyranosyl-(1→4)-(methyl 2-*O*-benzoyl-3-*O*-benzyl-α-L-idopyranosyl uronate)-(1→4)-2-azido-3,6-di-*O*-benzyl-2-deoxy-α-D-glucopyranosyl-(1→4)-

(methyl 2-*O*-benzoyl-3-*O*-benzyl-α-L-idopyranosyl uronate)-(1→4)-2-azido-3,6-di-*O*-benzyl-2-deoxy-α-D-glucopyranosyl-(1→4)-(methyl 2-*O*-benzoyl-3-*O*-benzyl-α-L-idopyranoside) uronate, 14

Acceptor dotriacontasaccharide **13** (93 mg, 0.008 mmol) and thioglycoside tetrasaccharide donor **1** (20 mg, 0.011 mmol) was dissolved in dry DCM (1 mL) under N_2. Freshly activated 4Å powdered molecular sieves (74 mg) was added and the solution cooled to 0 °C in an icebath. After 10 min. NIS (5 mg, 0.022 mmol) was added, and after another 10 min. AgOTf (catalytic amount) was added. The suspension changed colour from pale yellow to deep red, was stirred for 30 min. and the reaction was quenched into a separating funnel containing a mixture of DCM (20 mL), saturated aqueous $NaHCO_3$ (10 mL) and $Na_2S_2O_3$ (1 mL, 10% aqueous). After shaking until the iodine colour was removed the suspension was filtered through a short pad of Celite® washing with water and DCM. The layers were separated and the aqueous extracted with DCM (5 mL). The organic layers were combined, dried ($MgSO_4$) and solvent removed *in vacuo*. The crude was purified by preparative TLC (toluene/acetone 12:1) followed by precipitation (dissolved in warm EtOAc (4 mL) and hexane (4 mL) added) and filtration to give **14 (**76 mg, 72%) as a white solid. Also acceptor starting material (22mg, 23%) was recovered. R_f 0.28 (toluene/acetone 10:1). $[\alpha]_D^{20}$ = +38.8 (*c* = 0.45, CH_2Cl_2). ^{1}H NMR (800 MHz; $CDCl_3$) δ 8.09-8.08 (m, 2H, Bz), 8.01-8.00 (m, 2H, Bz), 7.95-7.91 (m, 32H, Bz), 7.54-7.02 (m, 324H, Ph), 5.57-5.52 (m, 17H, $H^{(BCDEFGHIJKLMNOPQR)ido}$-1), 5.30 (t, *J* = 9.6 Hz, 1H, H^{Rglcn}-4), 5.18-5.14 (m, 17H, $H^{(BCDEFGHIJKLMNOPQR)ido}$-2), 5.08-5.07 (m, 1H, H^{Aido}-2), 5.04-5.03 (m, 1H, H^{Aido}-1), 4.94-4.87 (m, 18H, $H^{(ABCDEFGHIJKLMNOPQR)glcn}$-1), 4.81-4.32 (m, 126H, $H^{(ABCDEFGHIJKLMNOPQR)ido}$-5, 54x$CH_2$Ph), 4.26-4.15 (m, 17H, $H^{(BCDEFGHIJKLMNOPQR)ido}$-3), 4.05-3.18 (m, 176H, H^{Aido}-3, $H^{(ABCDEFGHIJKLMNOPQR)ido}$ -4, $H^{(ABCDEFGHIJKLMNOPQR)glcn}$-2, $H^{(ABCDEFGHIJKLMNOPQR)glcn}$-3, $H^{(ABCDEFGHIJKLMOPQ)glcn}$ -4, $H^{(ABCDEFGHIJKLMNOPQR)glcn}$-5, $H^{(ABCDEFGHIJKLMNOPQR)glcn}$-$6_{ab}$, 18xCOO$CH_3$), 3.24 (s, 3H, O$CH_3$). ^{13}C NMR (201 MHz; $CDCl_3$): δ 169.6, 169.4, 165.7, 165.2, 138.0, 137.9, 137.5, 137.3, 136.9, 133.7, 133.5, 130.0, 129.9, 129.4, 129.0, 128.8, 128.5, 128.4, 128.1, 128.0, 127.9, 127.7, 127.6, 127.4, 99.3, 98.4, 98.1, 78.2, 77.0, 77.0, 76.1, 75.9, 75.6, 75.4, 75.1, 74.9, 74.5, 74.3, 73.9, 73.75, 73.5, 72.7, 72.6, 71.4, 70.8, 70.7, 69.8, 69.3, 67.4, 63.5, 63.1, 52.0, 51.7. MALDI TOF MS: m/z: calcd for $C_{741}H_{741}Cl_3N_{54}NaO_{200}$ [*M*+Na]$^+$: 13732; found: 13729.

Methyl (2-azido-3,6-di-*O*-benzyl-2-deoxy-α-D-glucopyranosyl)-(1→4)-(methyl 2-*O*-benzoyl-3-*O*-benzyl-α-L-idopyranosyl uronate)-(1→4)-2-azido-3,6-di-*O*-benzyl-2-deoxy-α-D-glucopyranosyl-(1→4)-(methyl 2-*O*-benzoyl-3-*O*-benzyl-α-L-idopyranosyl uronate)-(1→4)-2-azido-3,6-di-*O*-benzyl-2-deoxy-α-D-glucopyranosyl-(1→4)-(methyl 2-*O*-benzoyl-3-*O*-benzyl-α-L-idopyranosyl uronate)-(1→4)-2-azido-3,6-di-*O*-benzyl-2-deoxy-α-D-glucopyranosyl-(1→4)- (methyl 2-*O*-benzoyl-3-*O*-benzyl-α-L-idopyranosyl uronate)-(1→4)-2-azido-3,6-di-*O*-benzyl-2-deoxy-α-D-glucopyranosyl-(1→4)-(methyl 2-*O*-benzoyl-3-*O*-benzyl-α-L-idopyranosyl uronate)-(1→4)-2-azido-3,6-di-*O*-benzyl-2-deoxy-α-D-glucopyranosyl-(1→4)-(methyl 2-*O*-benzoyl-3-*O*-benzyl-α-L-idopyranosyl uronate)-(1→4)-2-azido-3,6-di-*O*-benzyl-2-deoxy-α-D-glucopyranosyl-(1→4)-(methyl 2-*O*-benzoyl-3-*O*-benzyl-α-L-idopyranosyl uronate)-(1→4)-2-azido-3,6-di-*O*-benzyl-2-deoxy-α-D-glucopyranosyl-(1→4)-(methyl 2-*O*-benzoyl-3-*O*-benzyl-α-L-idopyranosyl uronate)-(1→4)-2-azido-3,6-di-*O*-benzyl-2-deoxy-α-D-glucopyranosyl-(1→4)-(methyl 2-*O*-benzoyl-3-*O*-benzyl-α-L-idopyranosyl uronate)-(1→4)-2-azido-3,6-di-*O*-benzyl-2-deoxy-α-D-glucopyranosyl-(1→4)-(methyl 2-*O*-benzoyl-3-*O*-benzyl-α-L-idopyranosyl uronate)-(1→4)-2-azido-3,6-di-*O*-

benzyl-2-deoxy-α-D-glucopyranosyl-(1→4)-(methyl 2-*O*-benzoyl-3-*O*-benzyl-α-L-idopyranosyl uronate)-(1→4)-2-azido-3,6-di-*O*-benzyl-2-deoxy-α-D-glucopyranosyl-(1→4)-(methyl 2-*O*-benzoyl-3-*O*-benzyl-α-L-idopyranosyl uronate)-(1→4)-2-azido-3,6-di-*O*-benzyl-2-deoxy-α-D-glucopyranosyl-(1→4)-(methyl 2-*O*-benzoyl-3-*O*-benzyl-α-L-idopyranosyl uronate)-(1→4)-2-azido-3,6-di-*O*-benzyl-2-deoxy-α-D-glucopyranosyl-(1→4)-(methyl 2-*O*-benzoyl-3-*O*-benzyl-α-L-idopyranosyl uronate)-(1→4)-2-azido-3,6-di-*O*-benzyl-2-deoxy-α-D-glucopyranosyl-(1→4)-(methyl 2-*O*-benzoyl-3-*O*-benzyl-α-L-idopyranosyl uronate)-(1→4)-2-azido-3,6-di-*O*-benzyl-2-deoxy-α-D-glucopyranosyl-(1→4)-(methyl 2-*O*-benzoyl-3-*O*-benzyl-α-L-idopyranosyl uronate)-(1→4)-2-azido-3,6-di-*O*-benzyl-2-deoxy-α-D-glucopyranosyl-(1→4)-(methyl 2-*O*-benzoyl-3-*O*-benzyl-α-L-idopyranosyl uronate)-(1→4)-2-azido-3,6-di-*O*-benzyl-2-deoxy-α-D-glucopyranosyl-(1→4)-(methyl 2-*O*-benzoyl-3-*O*-benzyl-α-L-idopyranoside) uronate, 15

The hexatriacontasaccharide **14** (50.5 mg, 0.0037 mmol) was dissolved in a mixture of MeOH/ pyridine (2 mL/3 mL) and heated to 50 °C for 6 h. The solvents were evaporated and co-evaporated with toluene (2x5 mL). The crude product was purified using flash column chromatography (toluene/DCM/acetone 16:4:1 to 12:3:1) followed by precipitation (dissolved in warm EtOAc (4 mL) and hexane (4 mL) added) and filtration to yield **15** (43.7 mg, 87%) as a white solid. R_f 0.17 (toluene/acetone 10:1). ^{1}H NMR (400 MHz; $CDCl_3$) δ 8.12-8.10 (m, 2H, Bz), 8.00-7.99 (m, 2H, Bz), 7.98-7.92 (m, 32H, Bz), 7.56-7.02 (m, 324H, Ph), 5.59-5.54 (m, 17H, H$^{(BCDEFGHIJKLMNOPQR)ido}$-1), 5.23-5.17 (m, 17H, H$^{(BCDEFGHIJKLMNOPQR)ido}$-2), 5.09-5.08 (m, 1H, H^{Aido}-2), 5.05-5.04 (m, 1H, H^{Aido}-1), 4.94-4.88 (m, 18H, H$^{(ABCDEFGHIJKLMNOPQR)glcn}$-1), 4.82-4.33 (m, 126H, H$^{(ABCDEFGHIJKLMNOPQR)ido}$-5, 54xC$H_2$Ph), 4.26-4.16 (m, 17H, H$^{(BCDEFGHIJKLMNOPQR)ido}$-3), 4.06-3.22 (m, 177H, H^{Aido}-3, H$^{(ABCDEFGHIJKLMNOPQR)ido}$ -4, H$^{(ABCDEFGHIJKLMNOPQR)glcn}$-2, H$^{(ABCDEFGHIJKLMNOPQR)glcn}$-3, H$^{(ABCDEFGHIJKLMOPQR)glcn}$ -4, H$^{(ABCDEFGHIJKLMNOPQR)glcn}$-5, H$^{(ABCDEFGHIJKLMNOPQR)glcn}$-$6_{ab}$, 18xCOOC$H_3$), 3.24 (s, 3H, OC$H_3$), 2.58 (d, J = 2.4 Hz, 1H, O*H*). MALDI TOF MS: m/z: calcd for $C_{739}H_{742}N_{54}NaO_{199}$ $[M+Na]^+$: 13587; found: 13592.

Methyl (2-azido-3,6-di-*O*-benzyl-2-deoxy-4-*O*-trichloroacetyl-α-D-glucopyranosyl)-(1→4)-(methyl 2-*O*-benzoyl-3-*O*-benzyl-α-L-idopyranosyl uronate)-(1→4)-2-azido-3,6-di-*O*-benzyl-2-deoxy-α-D-glucopyranosyl-(1→4)-(methyl 2-*O*-benzoyl-3-*O*-benzyl-α-L-idopyranosyl uronate)-(1→4)-2-azido-3,6-di-*O*-benzyl-2-deoxy-α-D-glucopyranosyl-(1→4)-(methyl 2-*O*-benzoyl-3-*O*-benzyl-α-L-idopyranosyl uronate)-(1→4)-2-azido-3,6-di-*O*-benzyl-2-deoxy-α-D-glucopyranosyl-(1→4)- (methyl 2-*O*-benzoyl-3-*O*-benzyl-α-L-idopyranosyl uronate)-(1→4)-2-azido-3,6-di-*O*-benzyl-2-deoxy-α-D-glucopyranosyl-(1→4)-(methyl 2-*O*-benzoyl-3-*O*-benzyl-α-L-idopyranosyl uronate)-(1→4)-2-azido-3,6-di-*O*-benzyl-2-deoxy-α-D-glucopyranosyl-(1→4)-(methyl 2-*O*-benzoyl-3-*O*-benzyl-α-L-idopyranosyl uronate)-(1→4)-2-azido-3,6-di-*O*-benzyl-2-deoxy-α-D-glucopyranosyl-(1→4)-(methyl 2-*O*-benzoyl-3-*O*-benzyl-α-L-idopyranosyl uronate)-(1→4)-2-azido-3,6-di-*O*-benzyl-2-deoxy-α-D-glucopyranosyl-(1→4)-(methyl 2-*O*-benzoyl-3-*O*-benzyl-α-L-idopyranosyl uronate)-(1→4)-2-azido-3,6-di-*O*-benzyl-2-deoxy-α-D-glucopyranosyl-(1→4)-(methyl 2-*O*-benzoyl-3-*O*-benzyl-α-L-idopyranosyl uronate)-(1→4)-2-azido-3,6-di-*O*-benzyl-2-deoxy-α-D-glucopyranosyl-(1→4)-(methyl 2-*O*-benzoyl-3-*O*-benzyl-α-L-

idopyranosyl uronate)-(1→4)-2-azido-3,6-di-*O*-benzyl-2-deoxy-α-D-glucopyranosyl-(1→4)-(methyl 2-*O*-benzoyl-3-*O*-benzyl-α-L-idopyranosyl uronate)-(1→4)-2-azido-3,6-di-*O*-benzyl-2-deoxy-α-D-glucopyranosyl-(1→4)-(methyl 2-*O*-benzoyl-3-*O*-benzyl-α-L-idopyranosyl uronate)-(1→4)-2-azido-3,6-di-*O*-benzyl-2-deoxy-α-D-glucopyranosyl-(1→4)-(methyl 2-*O*-benzoyl-3-*O*-benzyl-α-L-idopyranosyl uronate)-(1→4)-2-azido-3,6-di-*O*-benzyl-2-deoxy-α-D-glucopyranosyl-(1→4)-(methyl 2-*O*-benzoyl-3-*O*-benzyl-α-L-idopyranosyl uronate)-(1→4)-2-azido-3,6-di-*O*-benzyl-2-deoxy-α-D-glucopyranosyl-(1→4)-(methyl 2-*O*-benzoyl-3-*O*-benzyl-α-L-idopyranosyl uronate)-(1→4)-2-azido-3,6-di-*O*-benzyl-2-deoxy-α-D-glucopyranosyl-(1→4)-(methyl 2-*O*-benzoyl-3-*O*-benzyl-α-L-idopyranosyl uronate)-(1→4)-2-azido-3,6-di-*O*-benzyl-2-deoxy-α-D-glucopyranosyl-(1→4)-(methyl 2-*O*-benzoyl-3-*O*-benzyl-α-L-idopyranosyl uronate)-(1→4)-2-azido-3,6-di-*O*-benzyl-2-deoxy-α-D-glucopyranosyl-(1→4)-(methyl 2-*O*-benzoyl-3-*O*-benzyl-α-L-idopyranoside) uronate, 16

Acceptor hexatriacontasaccharide **15** (42 mg, 0.0031 mmol) and thioglycoside tetrasaccharide donor **1** (8 mg, 0.0046 mmol) was dissolved in dry DCM (0.5 mL) under N_2. Freshly activated 4Å powdered molecular sieves (66 mg) was added and the solution cooled to 0 °C in an icebath. After 10 min. NIS (5 mg, 0.022 mmol) was added, and after another 10 min. AgOTf (catalytic amount) was added. The suspension changed colour from pale yellow to deep red, was stirred for 30 min. and the reaction was quenched into a separating funnel containing a mixture of DCM (20 mL), saturated aqueous $NaHCO_3$ (10 mL) and $Na_2S_2O_3$ (1 mL, 10% aqueous). After shaking until the iodine colour was removed the suspension was filtered through a short pad of Celite® washing with water and DCM. The layers were separated and the aqueous extracted with DCM (5 mL). The organic layers were combined, dried ($MgSO_4$) and solvent removed *in vacuo*. The crude product was purified using flash column chromatography (toluene/DCM/acetone 24:6:1 to 16:4:1 to 12:3:1) followed by precipitation (dissolved in warm EtOAc (2 mL) and hexane (1.5 mL) added) and filtration to give tetracontasaccharide **16** (30 mg, 64%) as a white solid. Also acceptor starting material (11 mg, 26%) was recovered. R_f 0.26 (toluene/acetone 10:1). ^{1}H NMR (800 MHz; $CDCl_3$) δ 8.10-8.09 (m, 2H, Bz), 8.02-8.01 (m, 2H, Bz), 7.95-7.91 (m, 36H, Bz), 7.55-7.02 (m, 360H, Ph), 5.58-5.53 (m, 19H, $H^{(BCDEFGHIJKLMNOPQRST)ido}$-1), 5.32 (t, J = 9.6 Hz, 1H, H^{Tglcn}-4), 5.19-5.15 (m, 19H, $H^{(BCDEFGHIJKLMNOPQRST)ido}$-2), 5.08-5.07 (m, 1H, H^{Aido}-2), 5.05-5.04 (m, 1H, H^{Aido}-1), 4.95-4.89 (m, 20H, $H^{(ABCDEFGHIJKLMNOPQRST)glcn}$-1), 4.81-4.33 (m, 140H, $H^{(ABCDEFGHIJKLMNOPQRST)ido}$-5, 60x$CH_2$Ph), 4.25-4.15 (m, 19H, $H^{(BCDEFGHIJKLMNOPQRST)ido}$-3), 4.06-3.16 (m, 200H, H^{Aido}-3, $H^{(ABCDEFGHIJKLMNOPQRST)ido}$ -4, $H^{(ABCDEFGHIJKLMNOPQRST)glcn}$-2, $H^{(ABCDEFGHIJKLMNOPQRST)glcn}$-3, $H^{(ABCDEFGHIJKLMOPQRS)glcn}$ -4, $H^{(ABCDEFGHIJKLMNOPQRST)glcn}$-5, $H^{(ABCDEFGHIJKLMNOPQRST)glcn}$-$6_{ab}$, 20xCOO$CH_3$), 3.23 (s, 3H, O$CH_3$). ^{13}C NMR (201 MHz; $CDCl_3$): δ 169.4, 165.2, 138.0, 137.9, 137.5, 133.7, 129.9, 129.4, 128.8, 128.5, 128.1, 128.0, 127.9, 127.7, 127.6, 127.4, 99.3, 98.1, 78.1, 76.1, 75.6, 75.4, 74.5, 74.3, 73.8, 72.7, 72.6, 71.5, 70.8, 70.7, 67.7, 67.4, 63.5, 63.3, 63.1, 52.1, 52.0, 51.8. MALDI TOF MS: m/z: calcd for $C_{823}H_{823}Cl_3N_{60}NaO_{222}$ $[M+Na]^+$: 15236; found: 15248.

Synthesis of icosasaccharide 18

a) The icosasaccharide **6** (40 mg, 0.0052 mmol) was dissolved in THF (2 mL) and MeOH (0.5 mL) and then cooled to 0 ºC in an icebath. Then $LiOH{\cdot}H_2O$ (14 mg, 0.33 mmol) dissolved in 0.5 mL water was added in portions over 20 min. The solution was stirred 12 h slowly warming to room temperature. The solution was then extracted with EtOAc (30 mL) and HCl (0.1 M, 20 mL). The organic phase was washed with water (2x10 mL), dried ($MgSO_4$), filtered and evaporated. The crude was purified using flash column chromatography (DCM/MeOH gradient 40:1 to 30:1). This

yielded the intermediate carboxylic acid icosasaccharide (16.0 mg) as solid. R_f 0.21 (DCM/MeOH 15:1).
b) The icosasaccharide acid (7.0 mg, 1.1 µmol) was dissolved in dry DMF (0.5 mL) under N_2 in a microwave compatible tube. $SO_3 \cdot NMe_3$ (8.5 mg, 60.5 µmol) was added and the solution heated in a microwave reactor for 1 h at 100 ºC. Tlc analysis (DCM:MeOH, 5:1) showed one product spot, a few drops of Et_3N were added (to neutralize any H_2SO_4 formed) and the reaction loaded onto a column of Sephadex LH20. The column was eluted with DCM/MeOH (9:1) and the fractions containing the oligosaccharide pooled and then passed though an additional column containing Amberlite IRC 86 Na^+ resin to convert the triethylammonium salts to sodium salt. This yielded 2-*O*-sulfated icosasaccharide **17** (7.5 mg) as a glassy solid. R_f= 0.21 (DCM:MeOH 5:1).
c) The icosasaccharide **17** (8 mg, 0.0010 mmol) was dissolved in a mixture of EtOH/H_2O (1 mL / 1 mL). Then a three way tap was attached to the flask and fitted with a nitrogen balloon and the other tap was attached to a water aspirator vacuum. Switching between vacuum and nitrogen balloon 5 times ensured removal of all oxygen from flask and solvent. Then was added $Pd(OH)_2/C$ (32 mg, 10-20%) and again flushed with nitrogen. The nitrogen balloon was replaced with a hydrogen balloon and the flask again subjected to vacuum and hydrogen 5 times to ensure all the nitrogen was replaced with hydrogen. The reaction was heated to 50 ºC for 36 h with vigorous stirring. The product mixture was filtered through Celite and washed with EtOH/water 1:2 (3x2 mL). The combined filtrate was then evaporated and purified by a short Sephadex G-25 column to give icosasaccharide amine **18 (**4.1 mg, 38% 3 steps) as a glassy solid. $[\alpha]_D^{20}$ = +57.3 (*c* = 0.18, H_2O). NMR data ring assignments coded from reducing terminal L-ido as ring A. ^{1}H NMR (400 MHz; D_2O) δ 5.35-5.29 (m, 9H, $H^{CEGIKMOQS}$-1), 5.22-5.16 (m, 10H, $H^{BDFHJLNPRT}$-1), 5.01-4.99 (m, 1H, H^{A}-1), 4.89-4.85 (m, 9H, $H^{CEGIKMOQS}$-5), 4.50-4.49 (m, 1H, H^{A}-5), 4.35-4.17 (m, 21H, $H^{ACEGIKMOQS}$-2, $H^{ACEGIKMOQS}$-3, H^{T}-4), 4.13-4.07 (m, 10H, $H^{ACEGIKMOQS}$-4), 3.90-3.66 (m, 49H, $H^{BDFHJLNPRT}$-3, $H^{BDFHJLNPR}$-4, $H^{BDFHJLNPRT}$-5, $H^{BDFHJLNPRT}$-6$_{ab}$), 3.39 (s, 3H, OMe), 3.26-3.18 (m, 10H, $H^{BDFHJLNPRT}$-2). ^{13}C NMR (201 MHz; D_2O): δ 175.3, 99.6, 99.0, 98.8, 91.7, 76.2, 76.0, 72.5, 71.4, 70.3, 70.1, 68.6, 66.9, 66.8, 66.2, 62.8, 62.6, 59.7, 59.3, 54.6, 54.1. NSI MS: m/z: calcd for $C_{121}H_{185}N_{10}O_{134}S_{11}$ $[M\text{-}9H]^{9-}$: 474.8327; found: 474.8324 (Na^+ ion exchanged with NH_4^+ before submitting for MS).[See **Figure 8**]

Synthesis of icosasaccharide 19

The icosasaccharide **18** (2.3 mg, 0.51 µmol) was dissolved in water (0.5 mL), $NaHCO_3$ (2.8 mg, 0.0335 mmol) and pyridine sulfur trioxide complex (2.4 mg, 0.0152 mmol) was added with stirring. This procedure was repeated after 1 h, 2 h, 4 h, 5 h, 7 h, 10 h, 12 h and 16 h ($NaHCO_3$: 6.7 mg, 9 mg, 8.5 mg, 10 mg, 7.3 mg, 13 mg, 9 mg. Py.SO_3: 6.5 mg, 7 mg, 5.7 mg, 6 mg, 5 mg, 10 mg, 5 mg.). After 20 h the mixture was evaporated. The crude containing Na_2SO_4 salts was redissolved in minimum amount of water and purified by passage through a Sephadex G-25 column (18x2.5 cm) by eluting with water. The fractions containing oligosaccharide were pooled and evaporated to yield **19 (**2.7 mg, 93%) as a glassy solid. $[\alpha]_D^{20}$ = +64.8 (*c* = 0.13, H_2O). ^{1}H NMR (800 MHz; D_2O) δ 5.36-5.25 (m, 19H, $H^{BCDEFGHIJKLMNOPQRST}$-1), 5.04-5.03 (m, 1H, H^{A}-1), 4.89-4.85 (m, 9H, $H^{CEGIKMOQS}$-5), 4.45 (d, *J* = 2.4 Hz, 1H, H^{A}-5), 4.37-4.29 (m, 9H, $H^{CEGIKMOQS}$-2), 4.27-4.21 (m, 11H, H^{A}-2, $H^{ACEGIKMOQS}$-3, H^{T}-4), 4.06-4.02 (m, 10H, $H^{ACEGIKMOQS}$-4), 3.93-3.81 (m, 30H, $H^{BDFHJLNPRT}$-5, $H^{BDFHJLNPRT}$-6$_{ab}$), 3.75-3.68 (m, 20H, $H^{BDFHJLNPRT}$-3, $H^{BDFHJLNPRT}$-4), 3.43 (s, 3H, OMe), 3.33-3.21 (m, 10H, $H^{BDFHJLNPRT}$-2). ^{13}C NMR (200 MHz; D_2O): δ 175.7, 99.8, 99.2, 97.3, 96.9, 77.3, 77.1, 75.8, 75.6, 75.0, 74.5, 71.8, 71.5, 71.3, 71.1, 69.5, 68.3, 68.2, 67.6, 59.7, 58.4, 58.3, 57.9, 55.3.

2. Spectral Data

Supplementary Figure 1: NUS Pure Shift HSQC (800 MHz, $CDCl_3$) for **2** (2centres region)

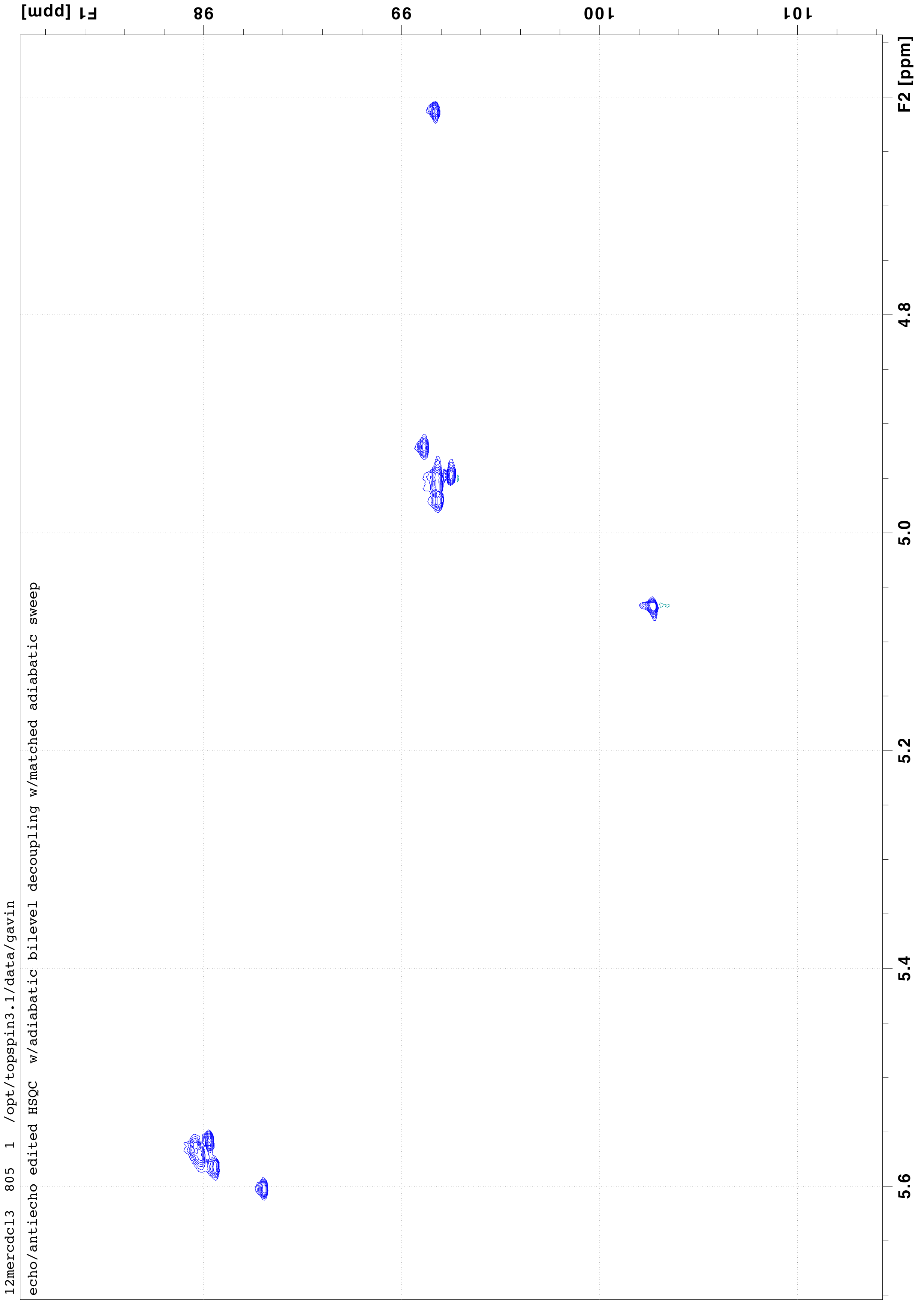

Supplementary Figure 2: NUS Pure Shift HSQC (800 MHz, $CDCl_3$) for **2** (anomeric centres region, comparison to standard HSQC (red)

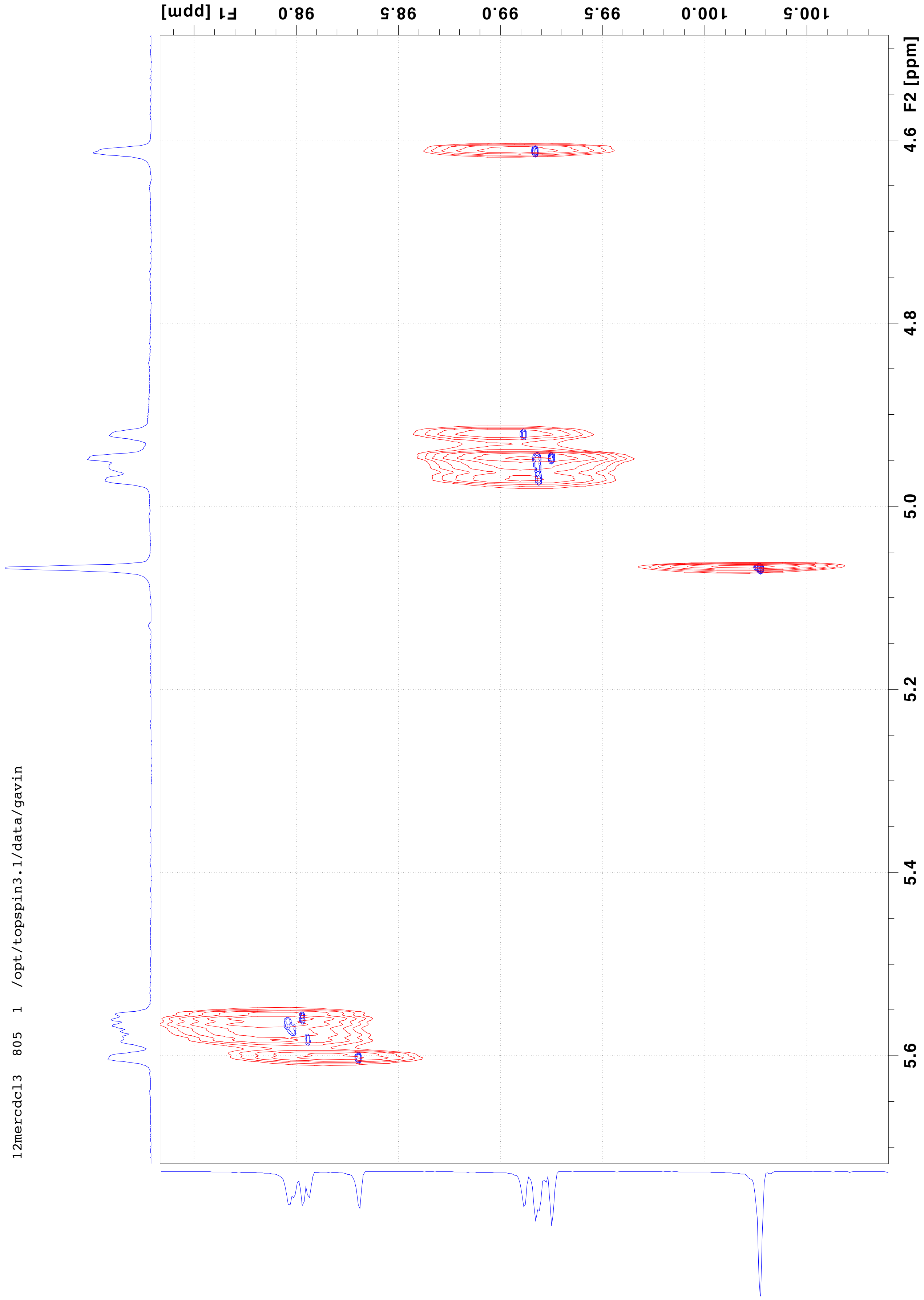

Supplementary Figure 3: ^{1}H NMR (400 MHz; $CDCl_3$) spectrum for **3**

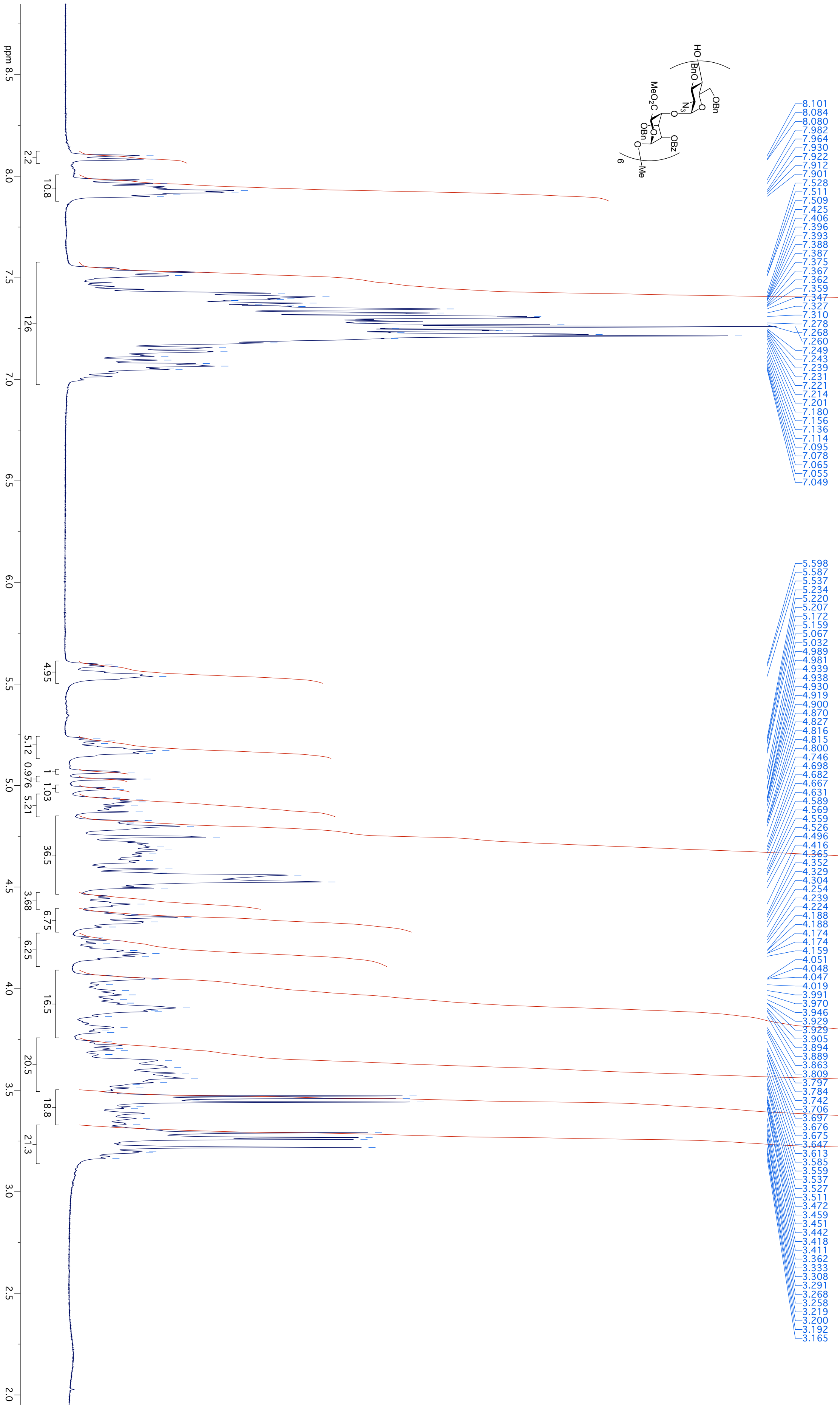

Supplementary Figure 4: COSY NMR (400 MHz; $CDCl_3$) spectrum for **3** **S1512**

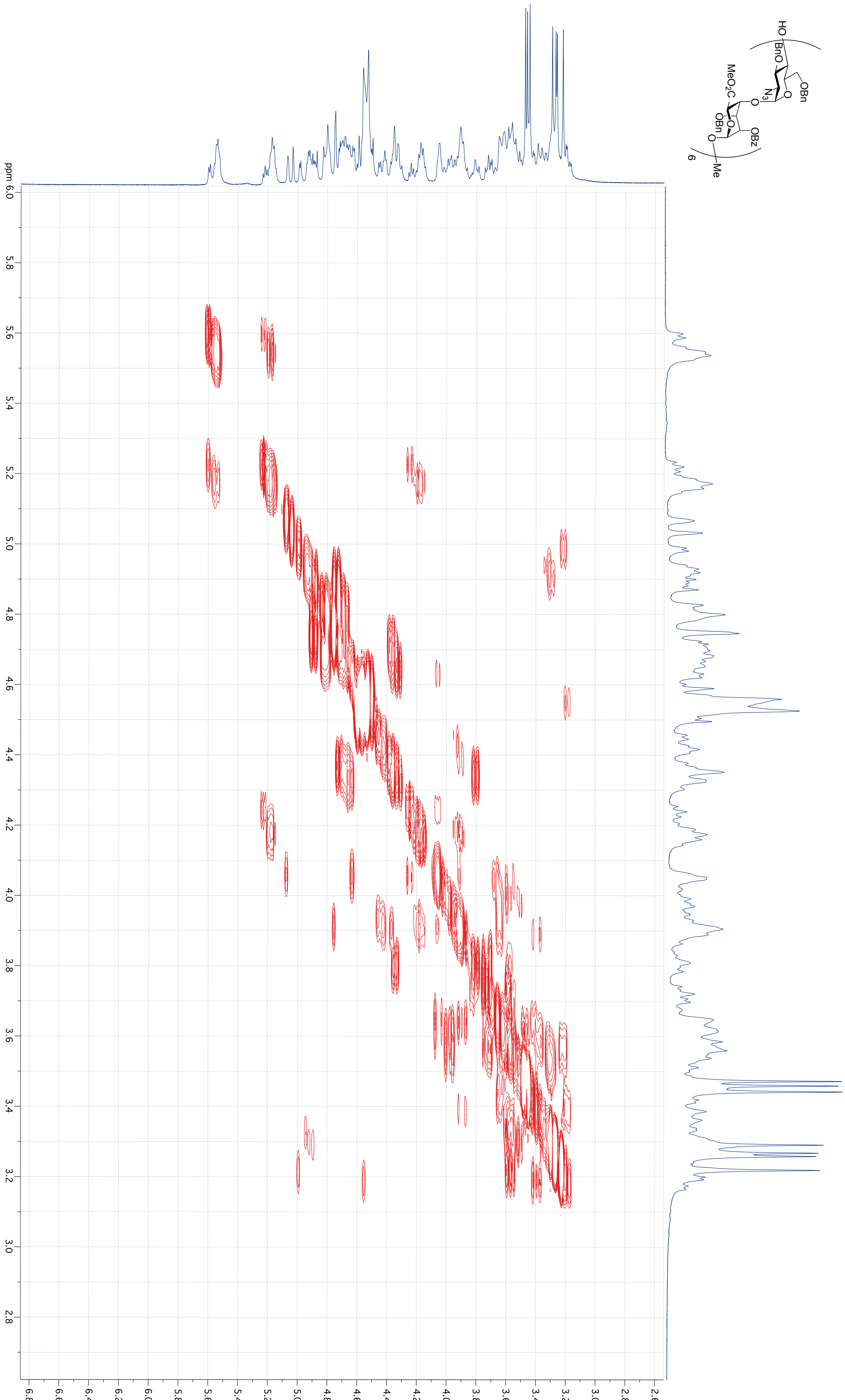

Supplementary Figure 5: HMQC NMR (400 MHz; $CDCl_3$) spectrum for **3**

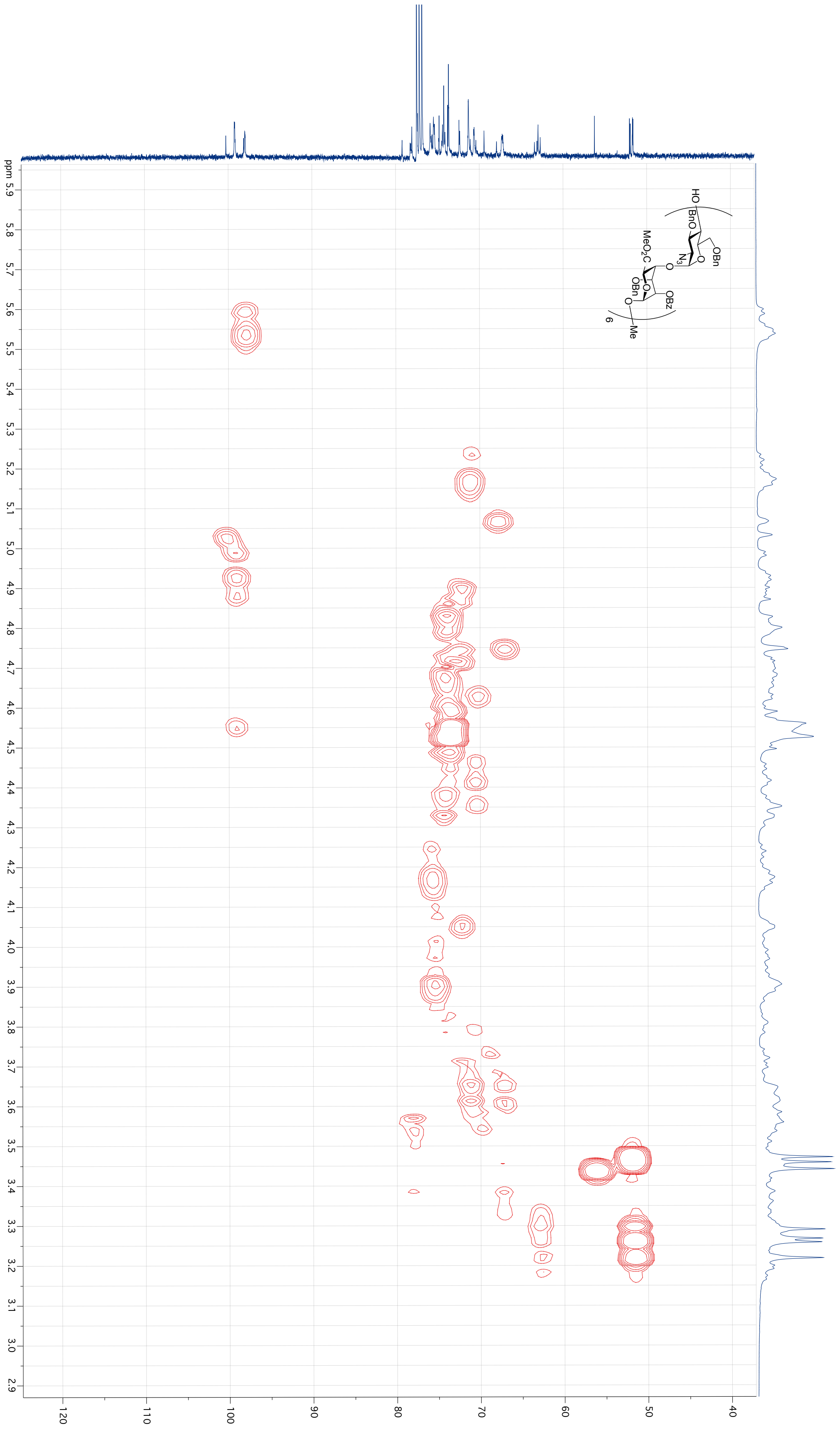

Supplementary Figure 6: ^{13}C NMR (100 MHz; $CDCl_3$) spectrum for **3**

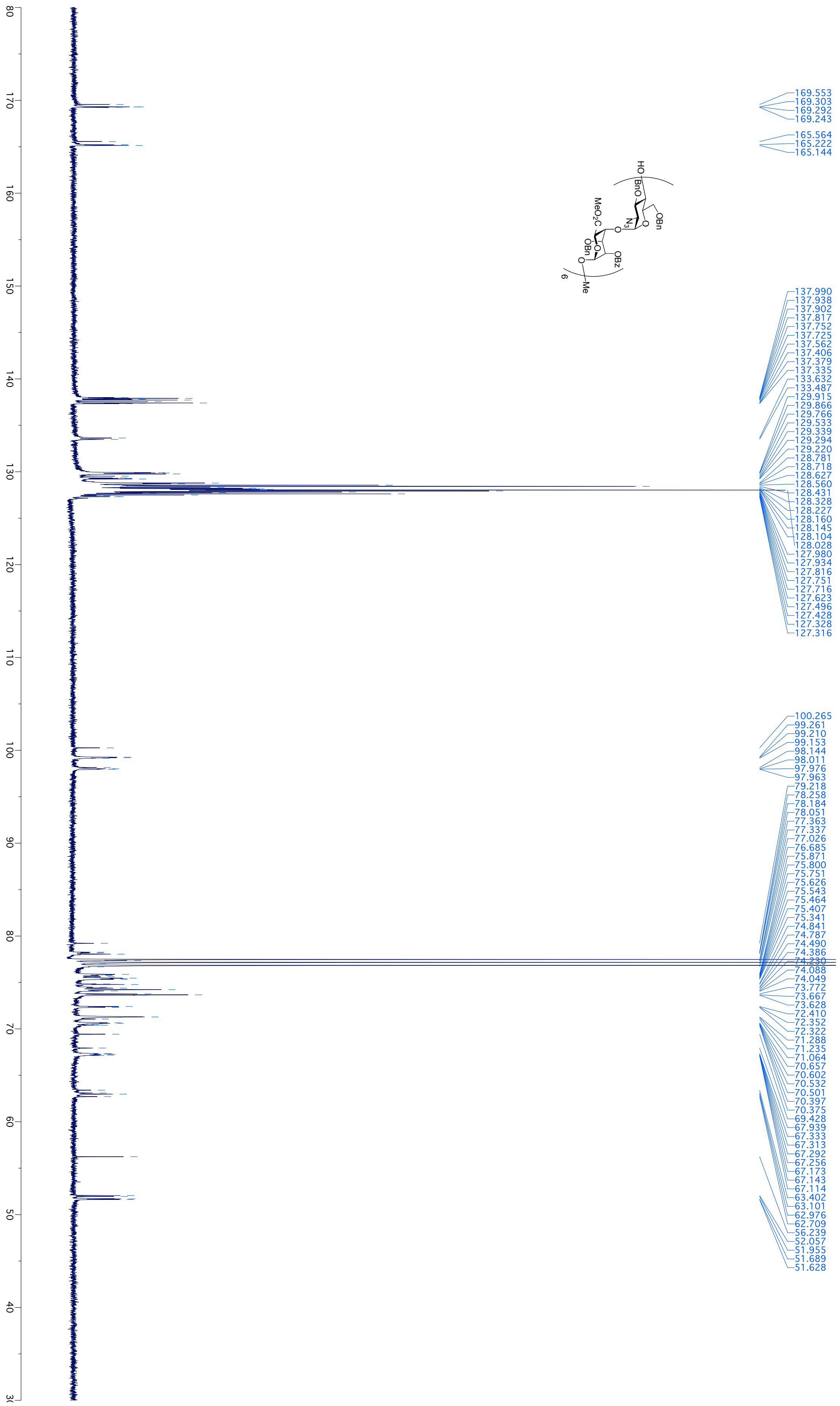

Supplementary Figure 7: MALDI MS and isotope pattern for **3**

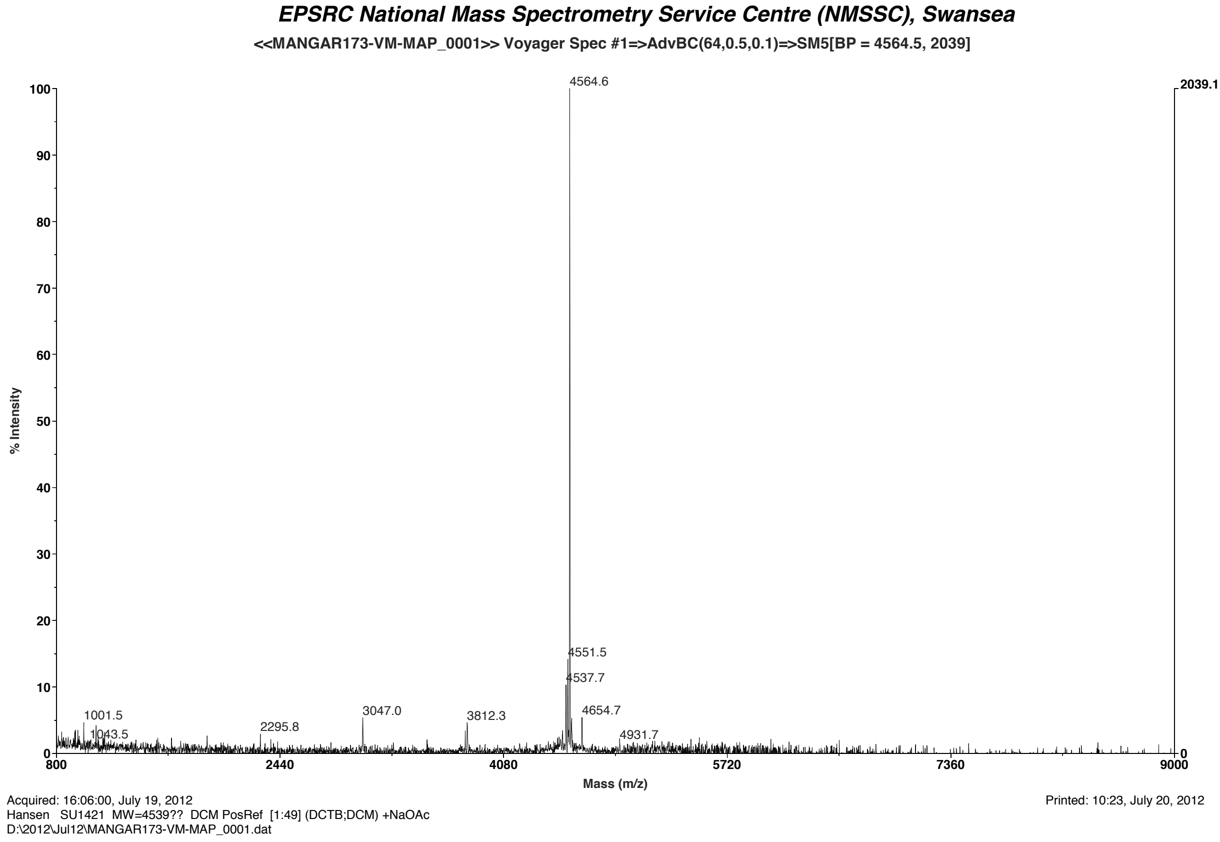

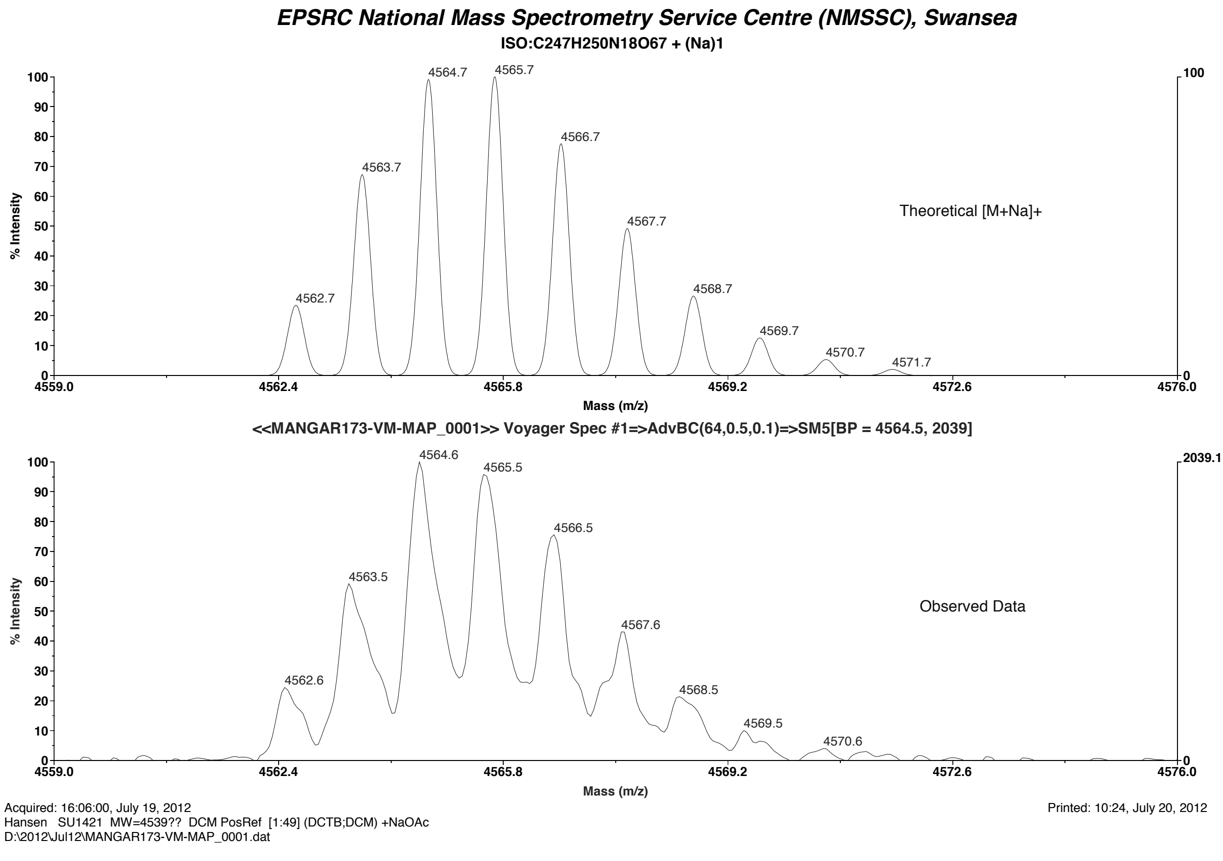

Supplementary Figure 8: LCMS for **3**

```
Data File C:\HPCHEM\1\DATA\G MILLER 27JUNE13\GM 1512B 27JUNE13.D
 Sample Name: GM 1512B 27June13
=====================================================================
Acq. Operator   : Rehana                          Seq. Line :   1
Acq. Instrument : Instrument 1                     Location : Vial 72
Injection Date  : 28/06/2013 12:57:52                   Inj :   1
                                                 Inj Volume : 20 µl
Different Inj Volume from Sequence !      Actual Inj Volume : 5 µl
Acq. Method     : C:\HPCHEM\1\METHODS\GAVIN MILLER 3CM COL 100ACN.M
Last changed    : 28/06/2013 12:56:57  by Rehana
Analysis Method : C:\HPCHEM\1\METHODS\REHANA 60MEOH WITH COL.m
Last changed    : 16/07/2013 12:16:45  by Rehana
Sample Info     : Gavin Miller    GM 1512B       27th June 2013
                  Supelco Ascentis C8 5u   4.6x30mm
                  ESI source  +ve ES 230nm 0.5ml/min
                  100ACN only  5ul inj

=====================================================================
```

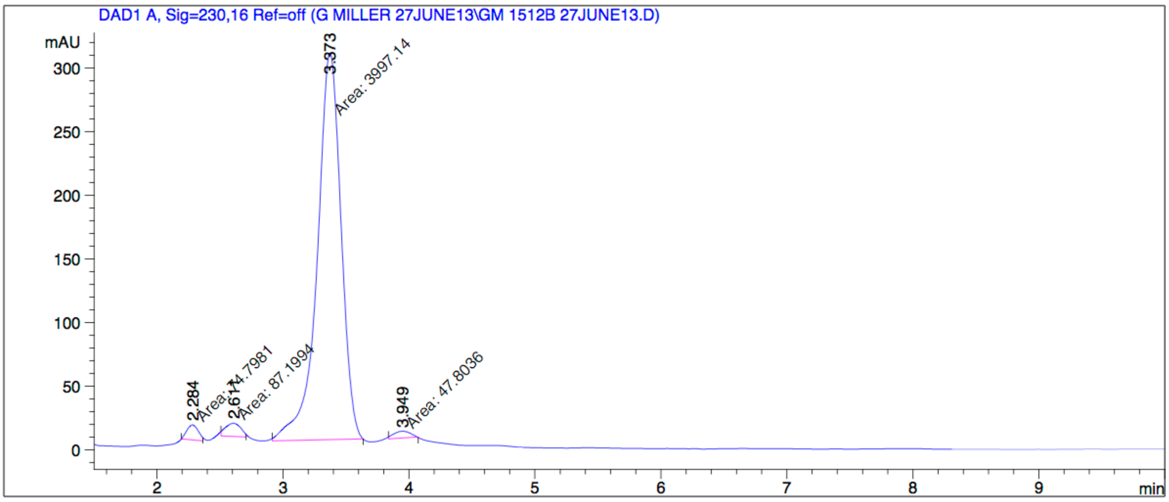


```
=====================================================================
                         Area Percent Report
=====================================================================

Sorted By             :      Signal
Multiplier            :      1.0000
Dilution              :      1.0000
Use Multiplier & Dilution Factor with ISTDs

Signal 1: DAD1 A, Sig=230,16 Ref=off
```

Peak #	RetTime [min]	Type	Width [min]	Area [mAU*s]	Height [mAU]	Area %
1	2.284	MM	0.1062	74.79813	11.73921	1.7780
2	2.611	MM	0.1417	87.19936	10.25990	2.0728
3	3.373	MM	0.2189	3997.13647	304.35858	95.0130
4	3.949	MM	0.1502	47.80359	5.30468	1.1363
Totals :				4206.93755	331.66238	

```
=====================================================================
                        *** End of Report ***
```

Instrument 1 16/07/2013 12:17:23 Rehana   Page   1 of 1

Supplementary Figure 9: LCMS for **3**

Display Report

Analysis Info

Analysis Name	GM 1512B 27JUNE13.D	Acquisition Date	28/06/2013 12:58:05
Method	GAVIN MILLER 3CM COL 100ACN.M	Operator	mib
Sample Name	GM 1512B 27June13	Instrument	LC-MSD-Trap-SL
Comment	Gavin Miller GM 1512B 27th June 2013<br>Supelco Ascentis C8 5u 4.6x30mm<br>ESI source +ve ES 230nm 0.5ml/min<br>100ACN only 5ul inj		

Acquisition Parameter

Ion Source Type	ESI	Ion Polarity	Positive	Alternating Ion Polarity	off
Mass Range Mode	Extended	Scan Begin	1000 m/z	Scan End	4000 m/z
Capillary Exit	280.0 Volt	Skim 1	40.0 Volt	Trap Drive	218.9
Accumulation Time	198516 µs	Averages	7 Spectra	Auto MS/MS	off

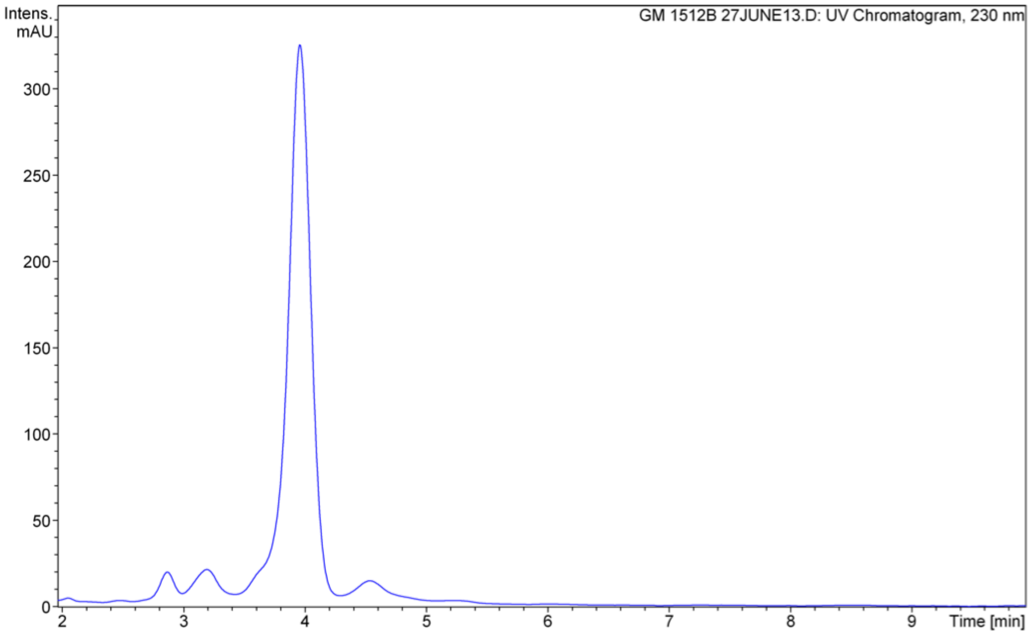

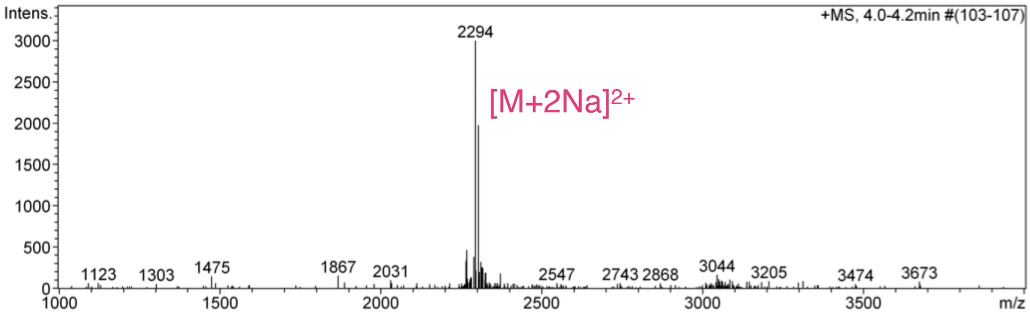

Bruker Daltonics DataAnalysis 3.1 printed: 03/07/2013 09:16:11 Page 1 of 1

Supplementary Figure 10: ^{1}H NMR (400 MHz; $CDCl_3$) spectrum for **4**

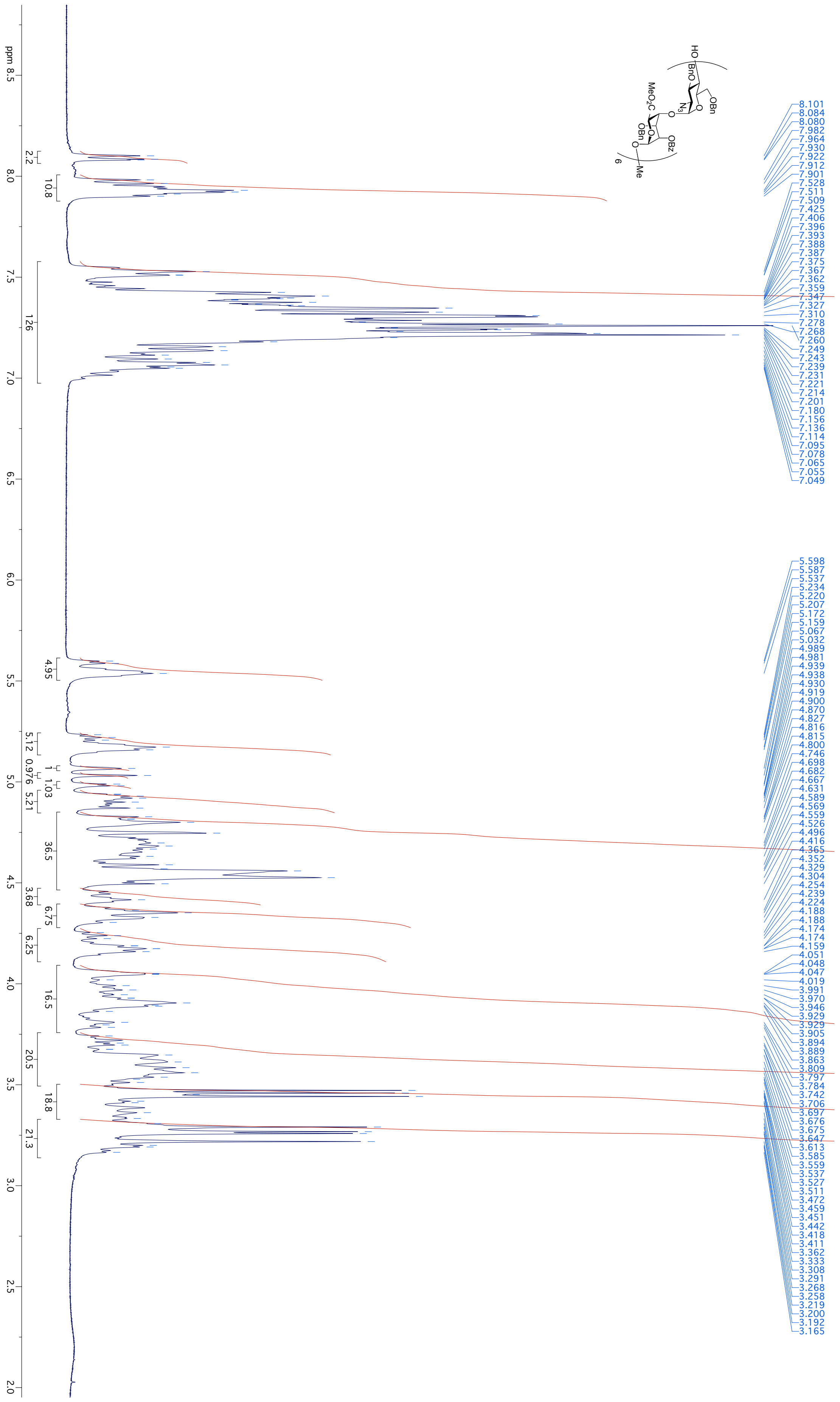

Supplementary Figure 11: COSY NMR (400 MHz; $CDCl_3$) spectrum for **4**

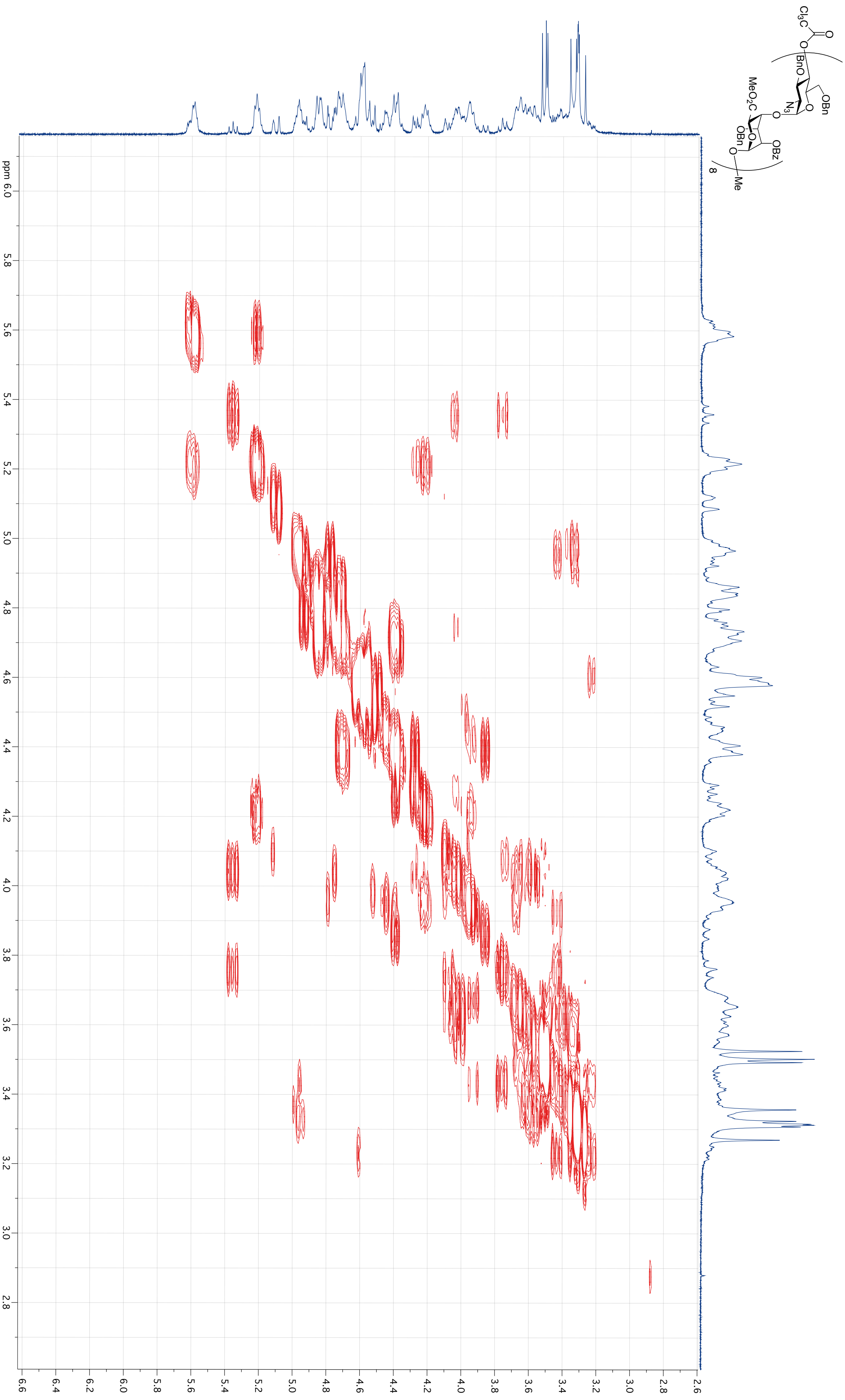

Supplementary Figure 12: HMQC NMR (400 MHz; $CDCl_3$) spectrum for **4**

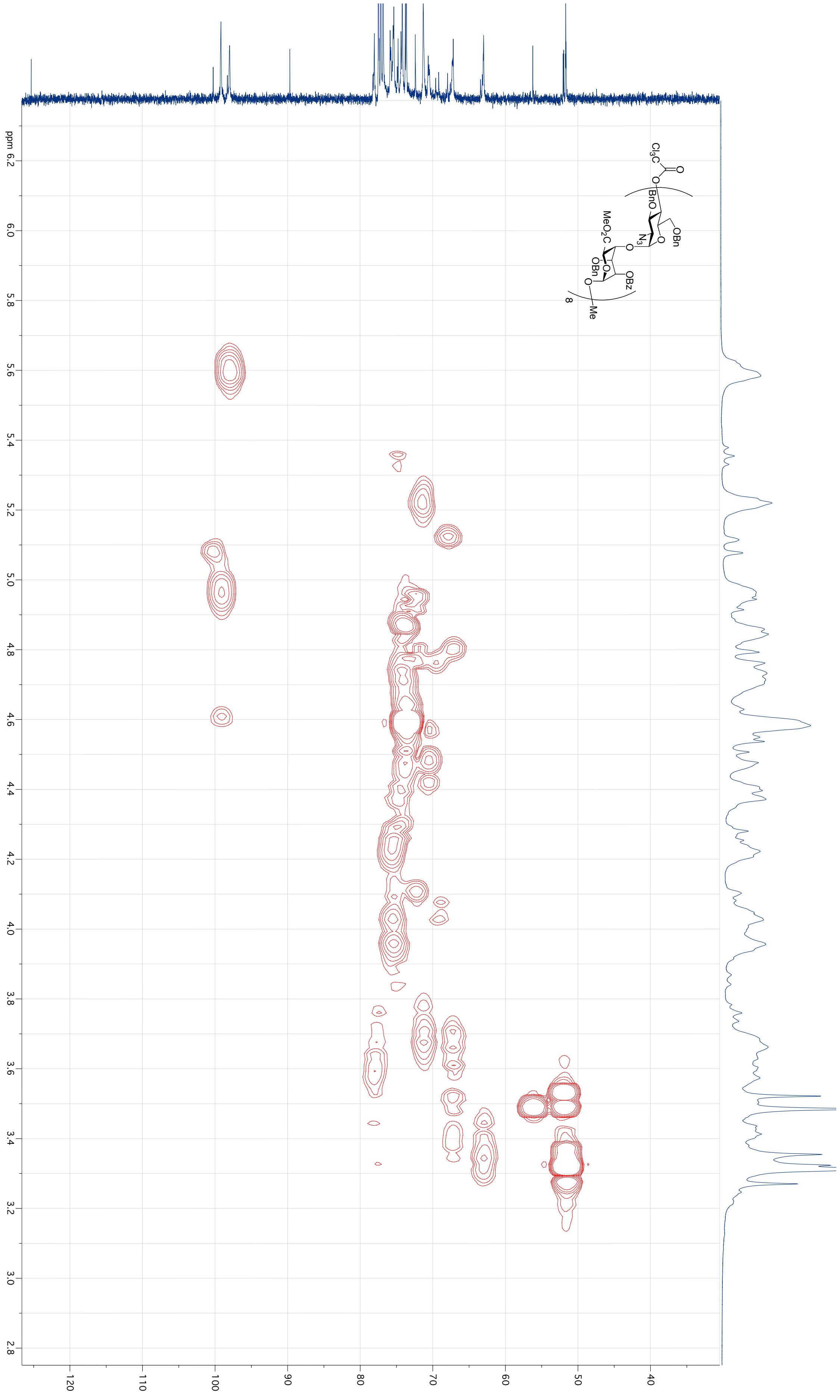

Supplementary Figure 13: NUS Pure Shift HSQC NMR (800 MHz; $CDCl_3$) spectrum for **4**

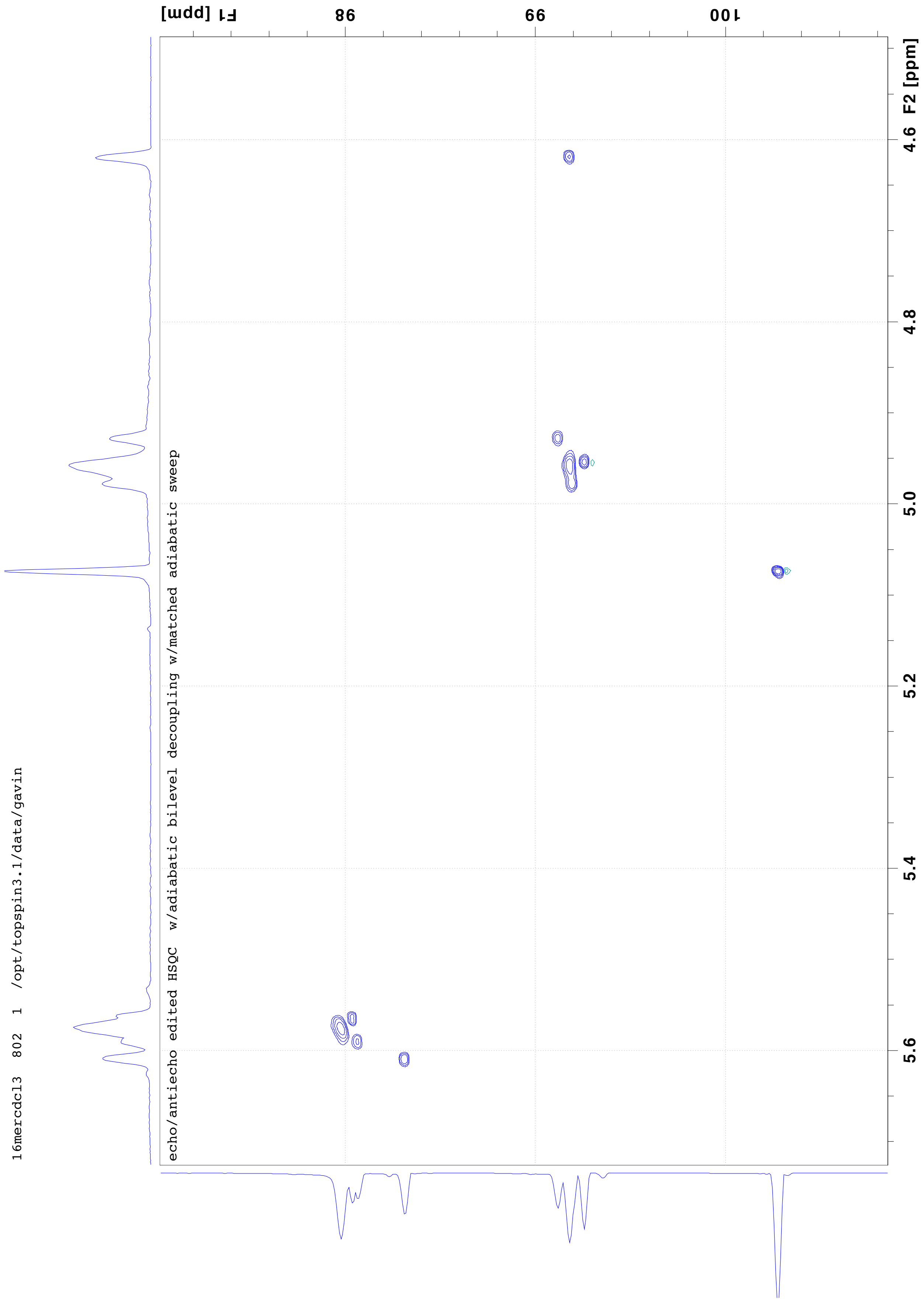

Supplementary Figure 14: NUS Pure Shift HSQC NMR (800 MHz; $CDCl_3$) spectrum for **4 - comparison to standard 800MHZ HSQC NMR**

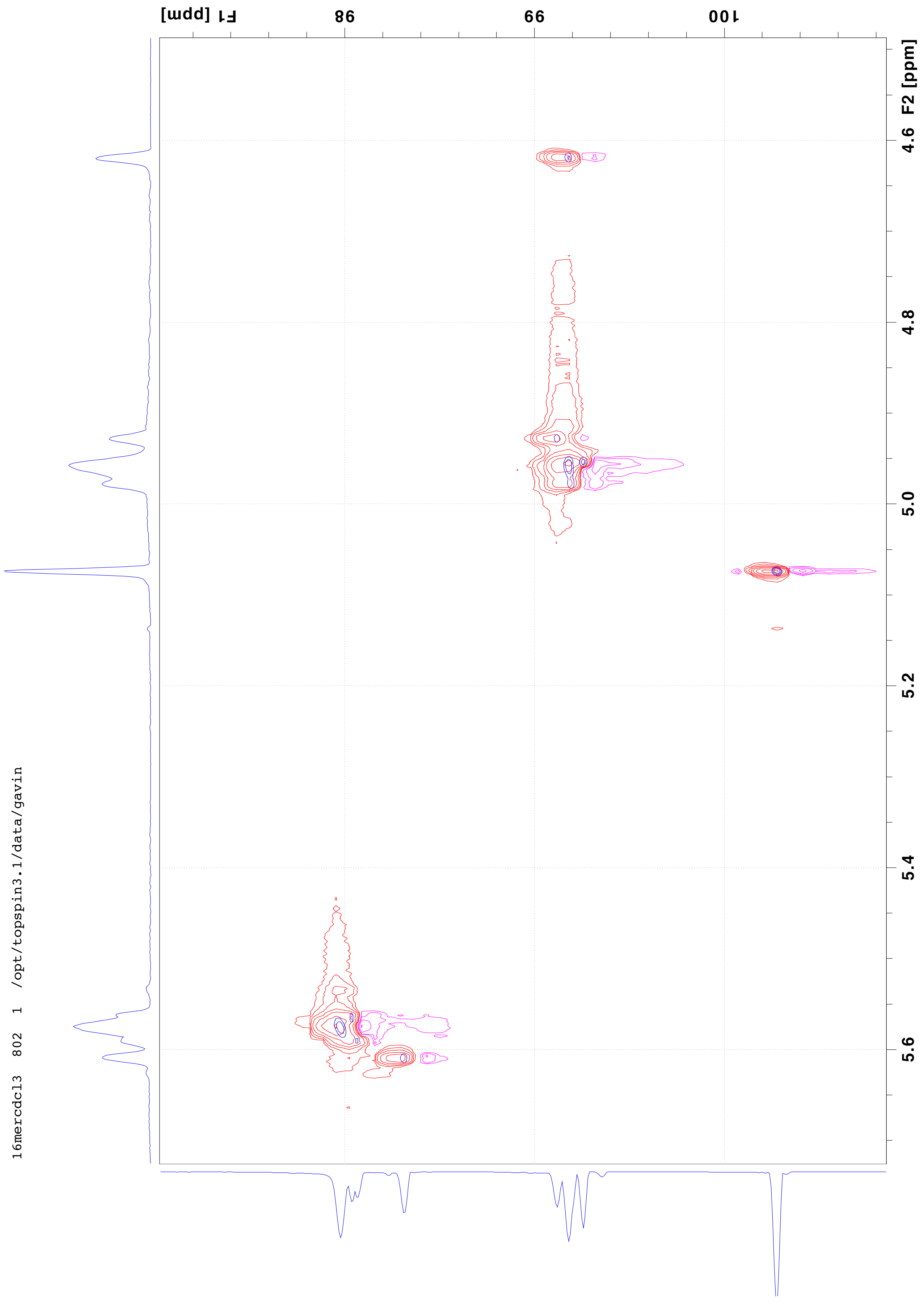

Supplementary Figure 15: NUS Pure Shift HSQC NMR (800 MHz; $CDCl_3$) spectrum for **4 (red) overlayed with 2 (12mer) (blue) - anomeric centres region**

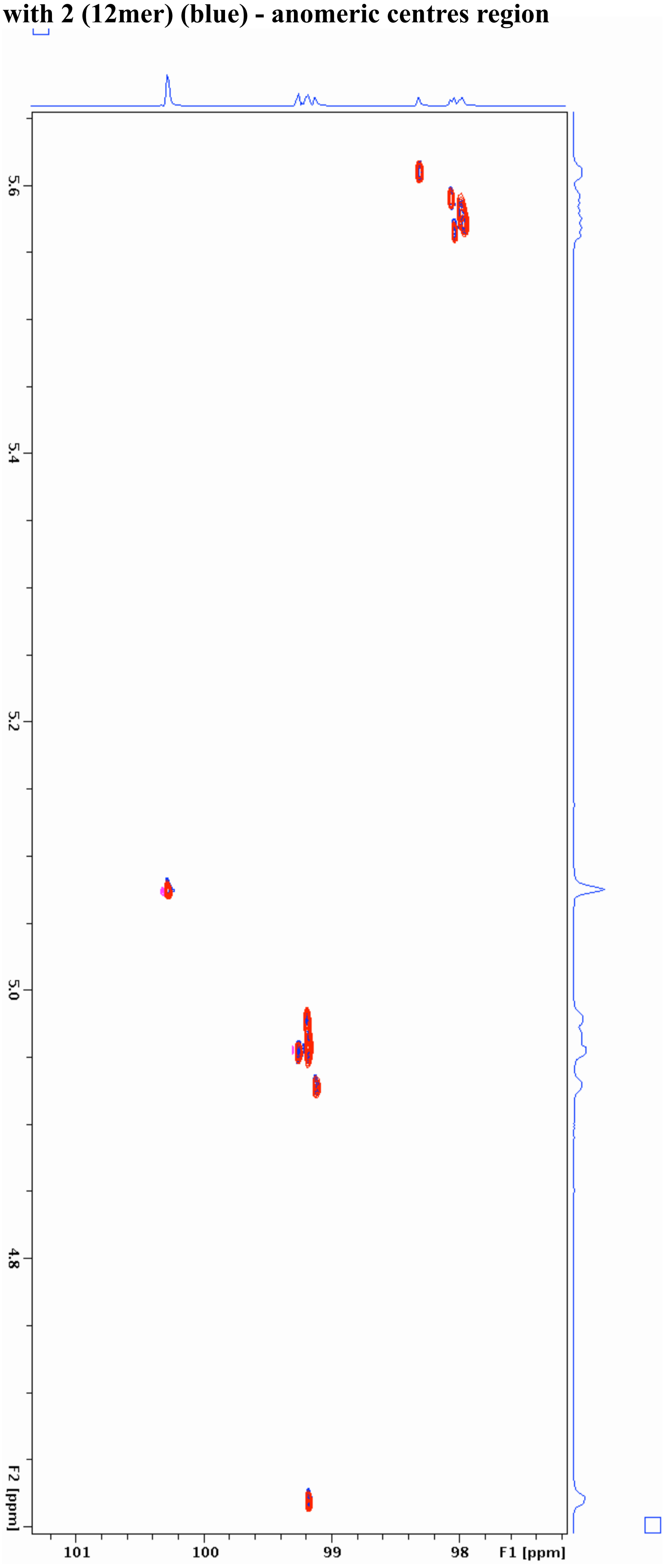

Supplementary Figure 16: ^{13}C NMR (100 MHz; $CDCl_3$) spectrum for **4**

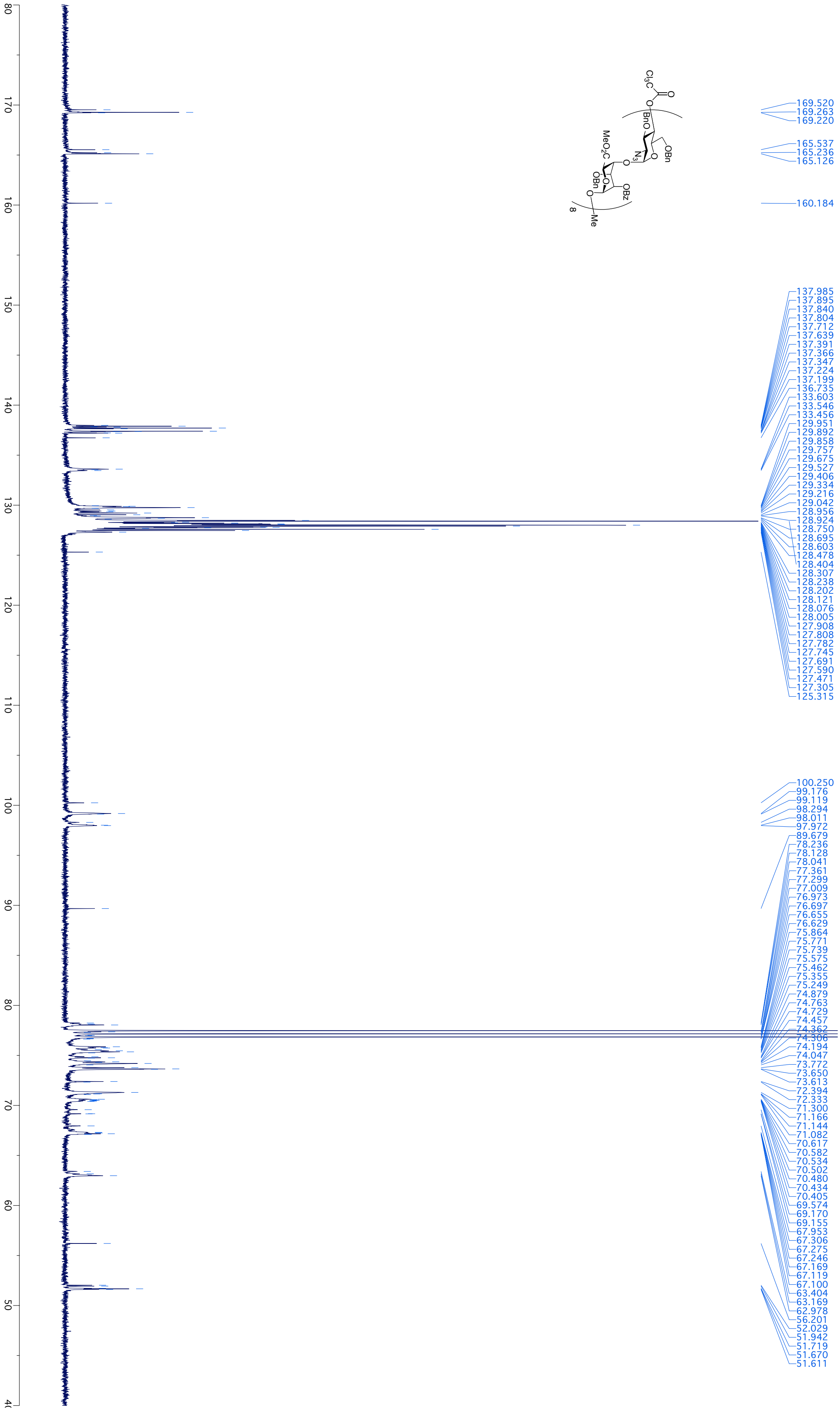

Supplementary Figure 17: MALDI MS for **4**

EPSRC National Mass Spectrometry Service Centre (NMSSC), Swansea

<<MANGAR186-VM-MAP_0001>> Voyager Spec #1=>AdvBC(64,0.5,0.1)=>SM5[BP = 518.9, 3580]

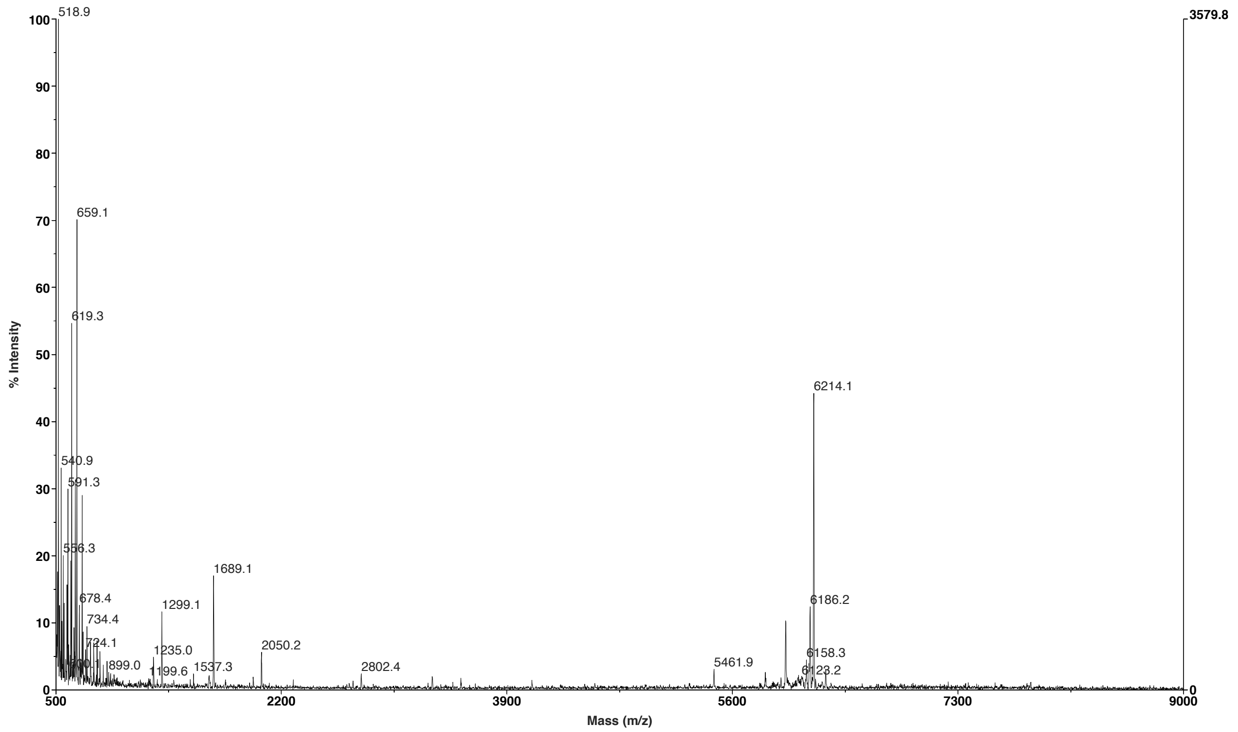

Acquired: 12:18:00, August 20, 2012
Hansen SU1514 MW=6186?? DCM PosRef [1:49] (Dith;DCM) +NaOAc
D:\2012\Aug12\MANGAR186-VM-MAP_0001.dat

Printed: 15:54, August 20, 2012

EPSRC National Mass Spectrometry Service Centre (NMSSC), Swansea

ISO:C331H331Cl3N24O90 + (Na)1

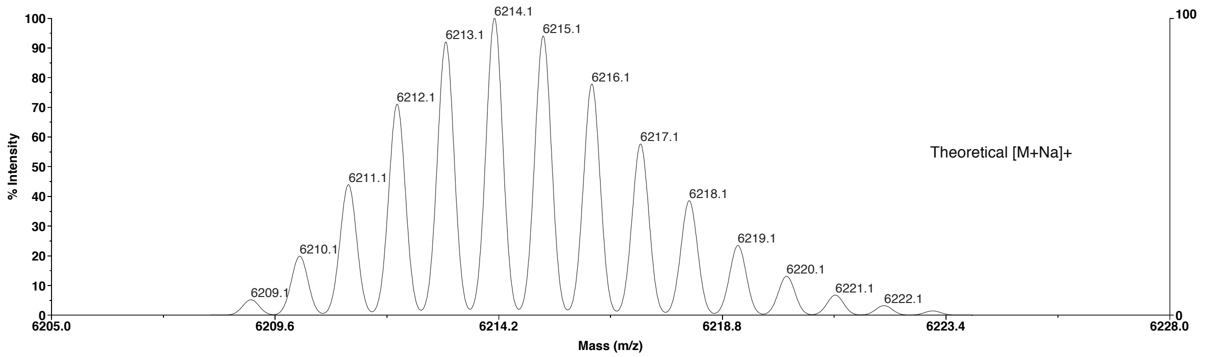

<<MANGAR186-VM-MAP_0001>> Voyager Spec #1=>AdvBC(64,0.5,0.1)=>SM5[BP = 518.9, 3580]

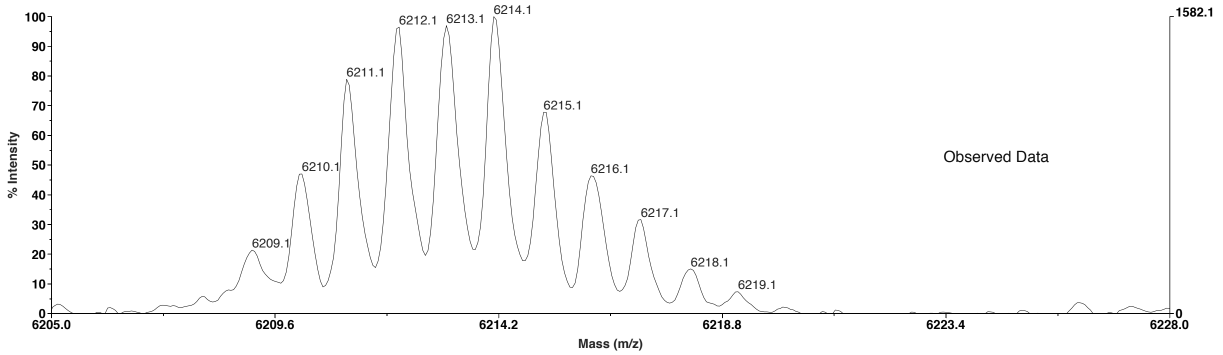

Acquired: 12:18:00, August 20, 2012
Hansen SU1514 MW=6186?? DCM PosRef [1:49] (Dith;DCM) +NaOAc
D:\2012\Aug12\MANGAR186-VM-MAP_0001.dat

Printed: 15:59, August 20, 2012

Supplementary Figure 18: LCMS for **4**

```
Data File C:\HPCHEM\1\DATA\G MILLER 27JUNE13\GM 1514 27JUNE13.D
 Sample Name: GM 1514 27June13
    =====================================================================
    Acq. Operator   : Rehana                              Seq. Line :   1
    Acq. Instrument : Instrument 1                         Location : Vial 73
    Injection Date  : 28/06/2013 13:20:10                       Inj :   1
                                                         Inj Volume : 20 µl
    Different Inj Volume from Sequence !      Actual Inj Volume : 5 µl
    Acq. Method     : C:\HPCHEM\1\METHODS\GAVIN MILLER 3CM COL 100ACN.m
    Last changed    : 28/06/2013 13:19:15  by Rehana
    Analysis Method : C:\HPCHEM\1\METHODS\REHANA 60MEOH WITH COL.m
    Last changed    : 16/07/2013 12:20:54  by Rehana
    Sample Info     : Gavin Miller    GM 1514        27th June 2013
                      Supelco Ascentis C8 5u  4.6x30mm
                      ESI source  +ve ES 230nm 0.5ml/min
                      100ACN only  5ul inj

    =====================================================================
```

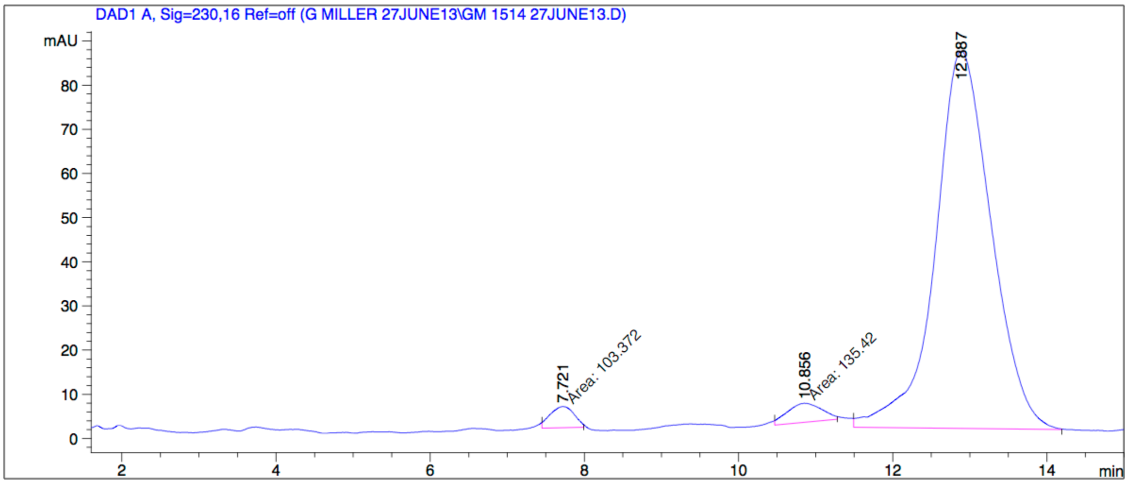


```
    =====================================================================
                             Area Percent Report
    =====================================================================

    Sorted By             :      Signal
    Multiplier            :      1.0000
    Dilution              :      1.0000
    Use Multiplier & Dilution Factor with ISTDs


    Signal 1: DAD1 A, Sig=230,16 Ref=off
```

Peak #	RetTime [min]	Type	Width [min]	Area [mAU*s]	Height [mAU]	Area %
1	7.721	MM	0.3598	103.37182	4.78776	2.2691
2	10.856	MM	0.5198	135.41997	4.34194	2.9726
3	12.887	VB	0.7506	4316.81592	85.56736	94.7583
Totals :				4555.60770	94.69706	

```
    =====================================================================
                              *** End of Report ***
```

Supplementary Figure 19: LCMS for **4**

Display Report

Analysis Info

Analysis Name	GM 1514 27JUNE13.D	Acquisition Date	28/06/2013 13:20:24
Method	GAVIN MILLER 3CM COL 100ACN.M	Operator	mib
Sample Name	GM 1514 27June13	Instrument	LC-MSD-Trap-SL
Comment	Gavin Miller GM 1514 27th June 2013<br>Supelco Ascentis C8 5u 4.6x30mm<br>ESI source +ve ES 230nm 0.5ml/min<br>100ACN only 5ul inj		

Acquisition Parameter

Ion Source Type	ESI	Ion Polarity	Positive	Alternating Ion Polarity	off
Mass Range Mode	Extended	Scan Begin	1000 m/z	Scan End	4000 m/z
Capillary Exit	280.0 Volt	Skim 1	40.0 Volt	Trap Drive	218.9
Accumulation Time	199856 µs	Averages	7 Spectra	Auto MS/MS	off

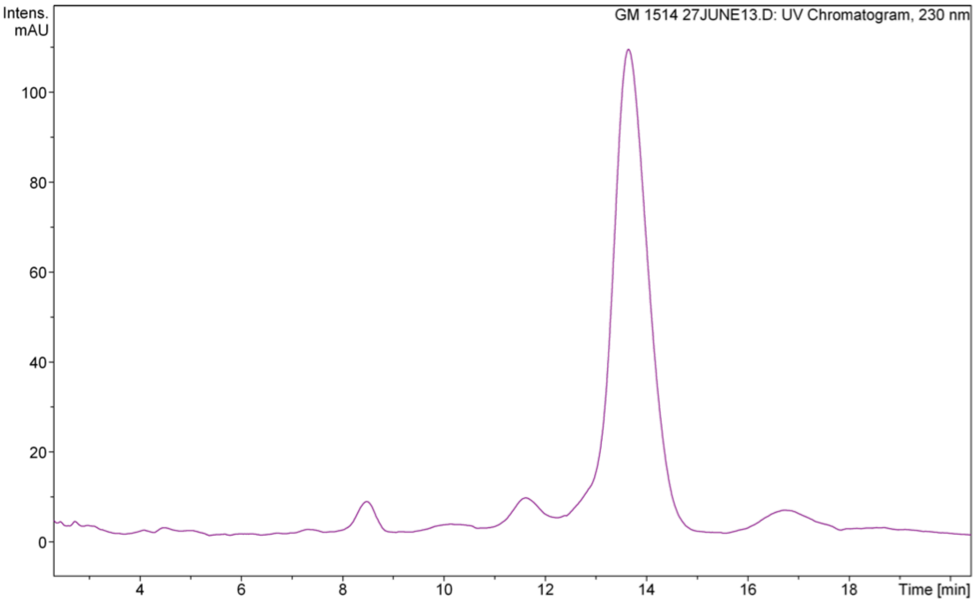

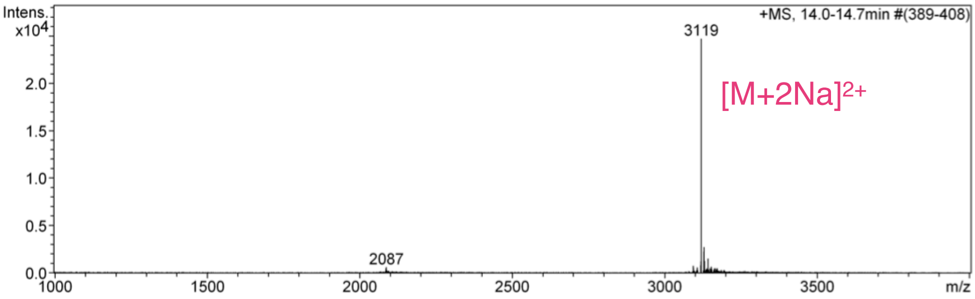

Bruker Daltonics DataAnalysis 3.1 printed: 03/07/2013 09:19:32 Page 1 of 1

Supplementary Figure 20: ^{1}H NMR (400 MHz; CDCl$_3$) spectrum for **5**

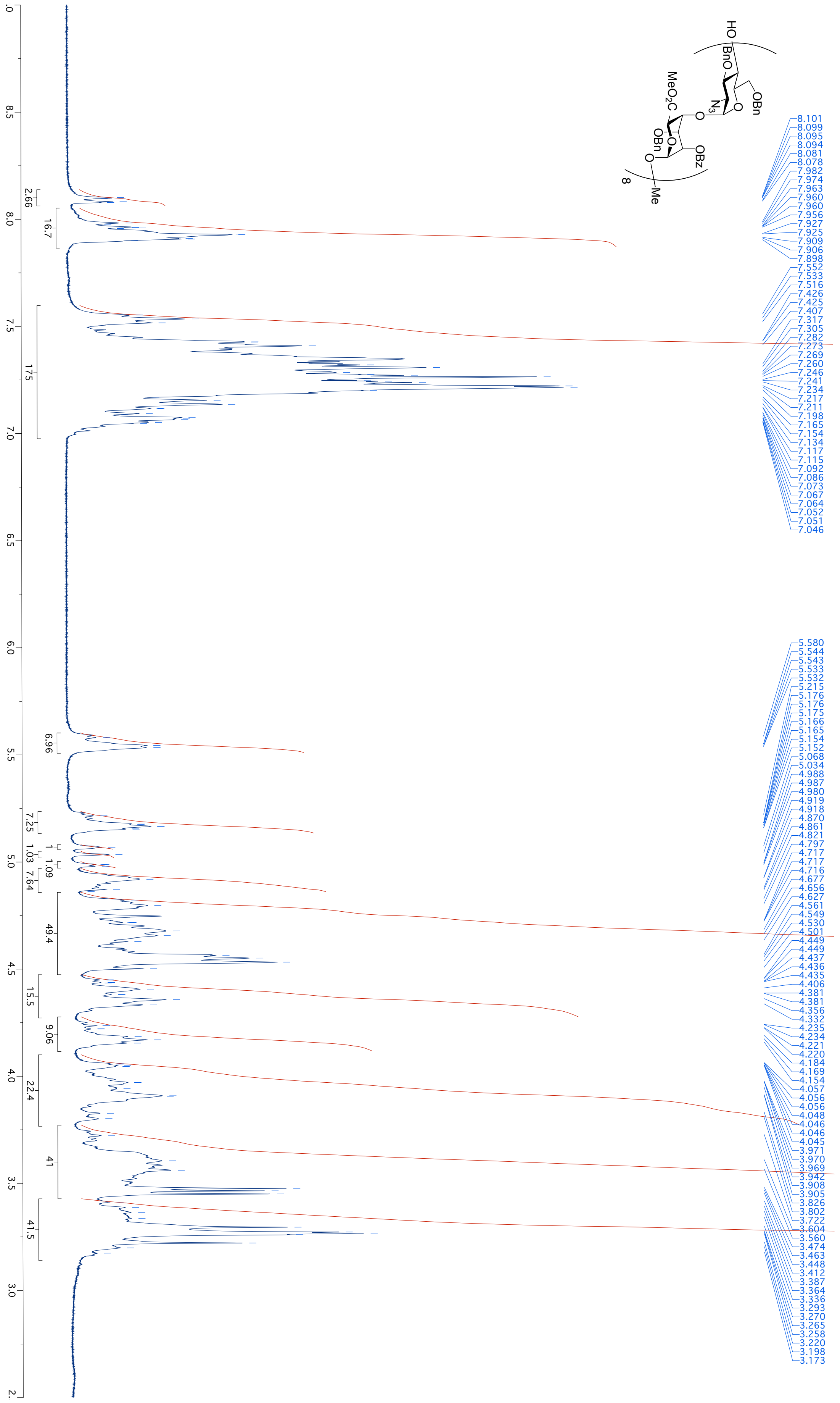

Supplementary Figure 21: COSY NMR (400 MHz; $CDCl_3$) spectrum for **5**

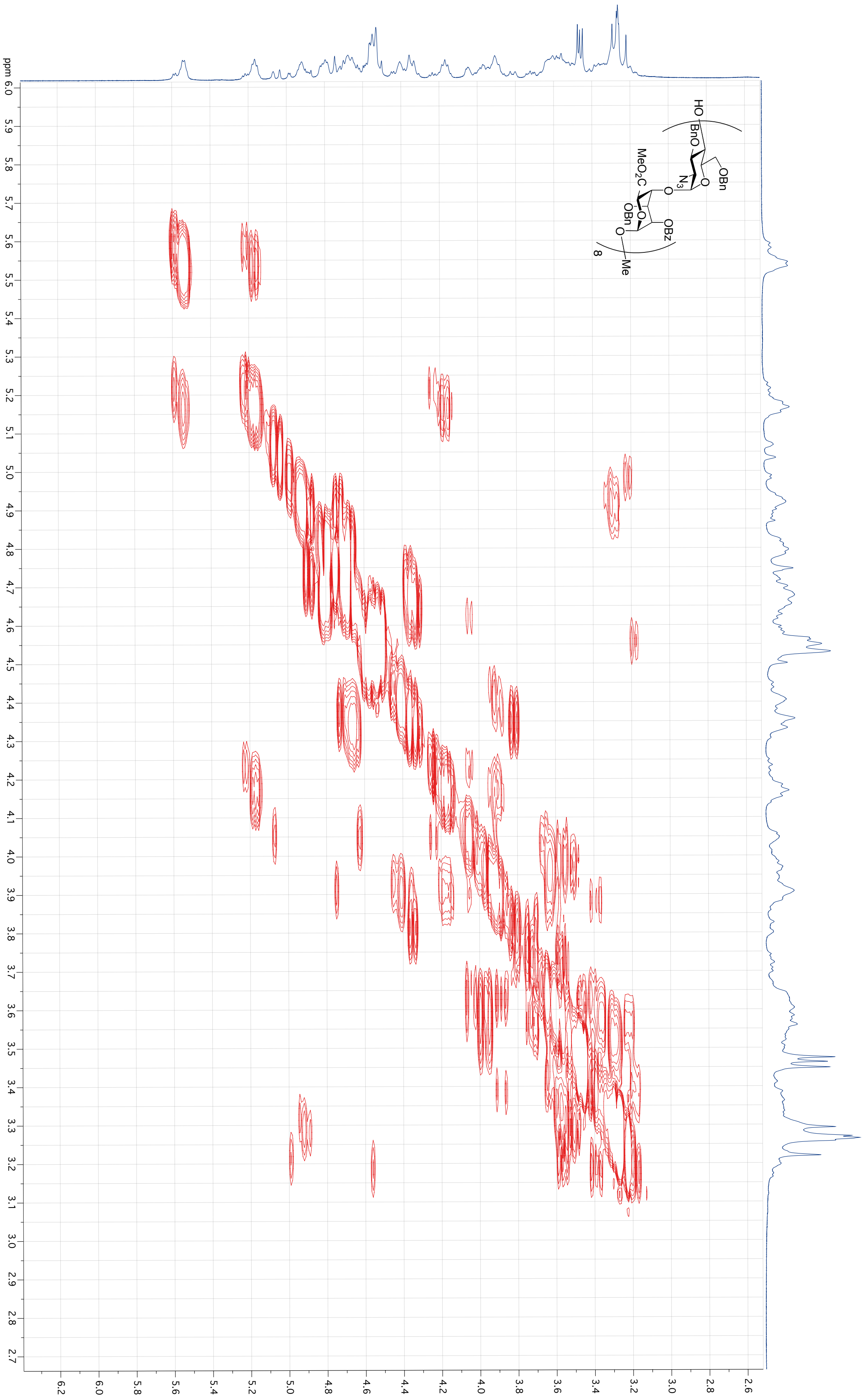

Supplementary Figure 22: HMQC NMR (400 MHz; $CDCl_3$) spectrum for **5**

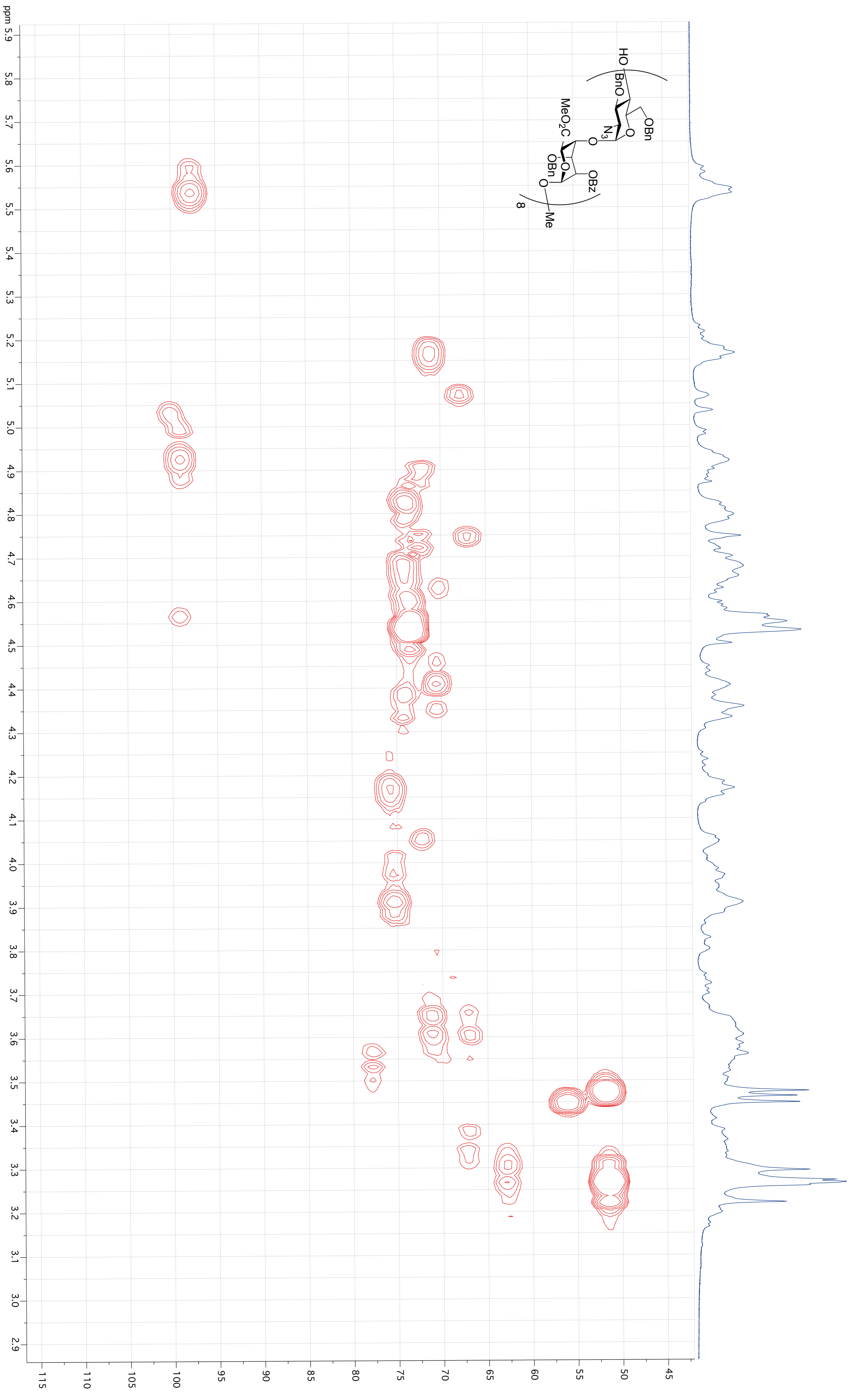

Supplementary Figure 23: MALDI MS and isotope pattern for **5**

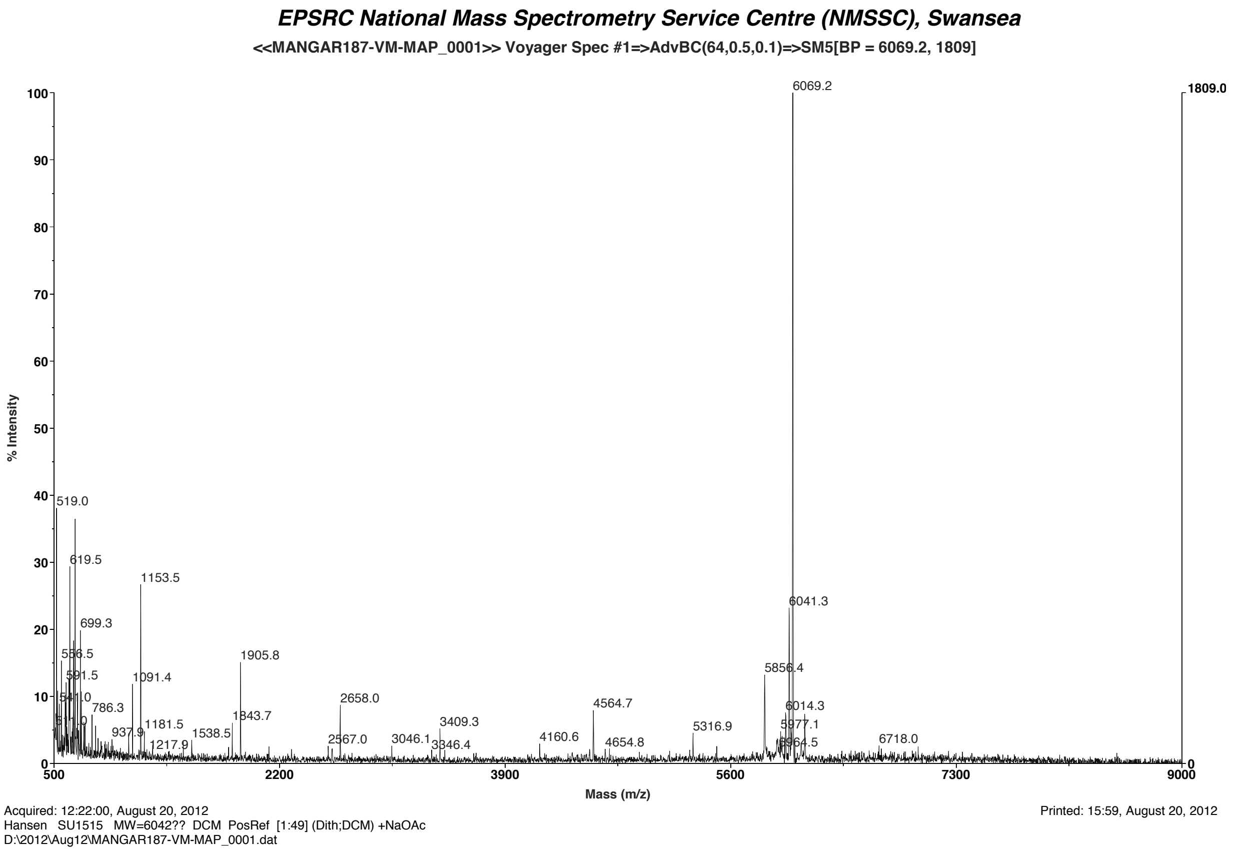

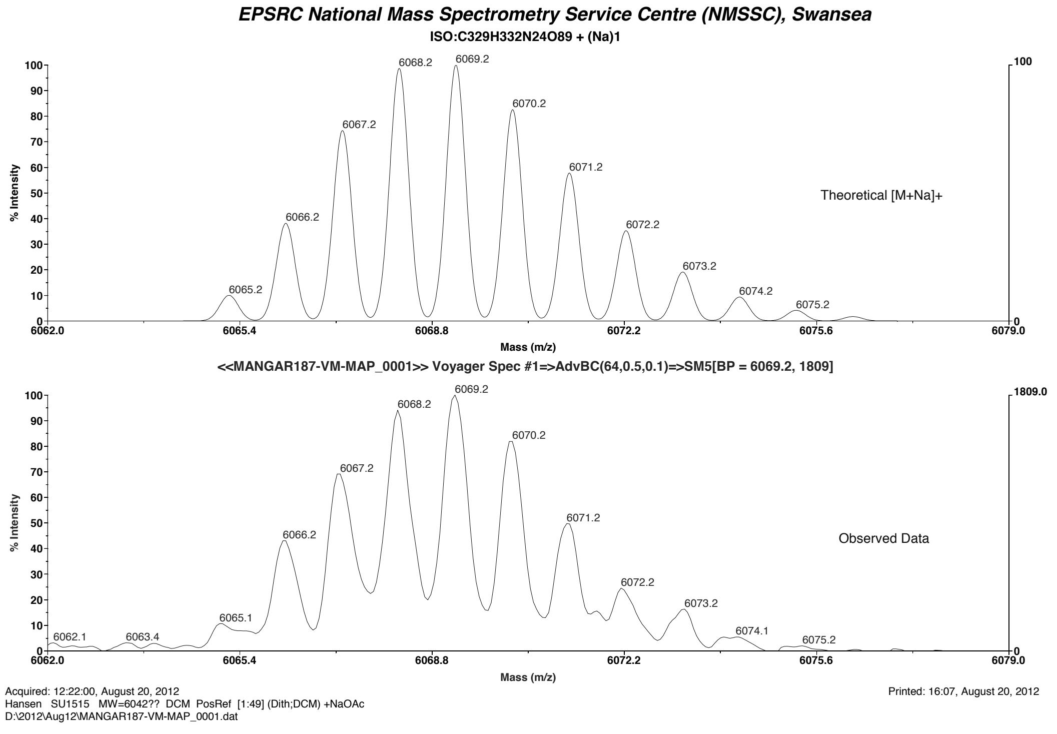

Supplementary Figure 24: LCMS for **5**

```
Data File C:\HPCHEM\1\DATA\G MILLER 27JUNE13\GM 1515 27JUNE13.D
 Sample Name: GM 1515 27June13
    =====================================================================
    Acq. Operator   : Rehana                           Seq. Line :    1
    Acq. Instrument : Instrument 1                      Location : Vial 74
    Injection Date  : 28/06/2013 14:56:16                    Inj :    1
                                                      Inj Volume : 20 µl
    Different Inj Volume from Sequence !      Actual Inj Volume : 5 µl
    Acq. Method     : C:\HPCHEM\1\METHODS\GAVIN MILLER 3CM COL 100ACN.M
    Last changed    : 28/06/2013 14:55:15  by Rehana
    Analysis Method : C:\HPCHEM\1\METHODS\REHANA 60MEOH WITH COL.m
    Last changed    : 16/07/2013 12:19:21  by Rehana
    Sample Info     : Gavin Miller     GM 1515         27th June 2013
                      Supelco Ascentis C8 5u  4.6x30mm
                      ESI source  +ve ES 230nm 0.5ml/min
                      100ACN only  5ul inj

    =====================================================================
```

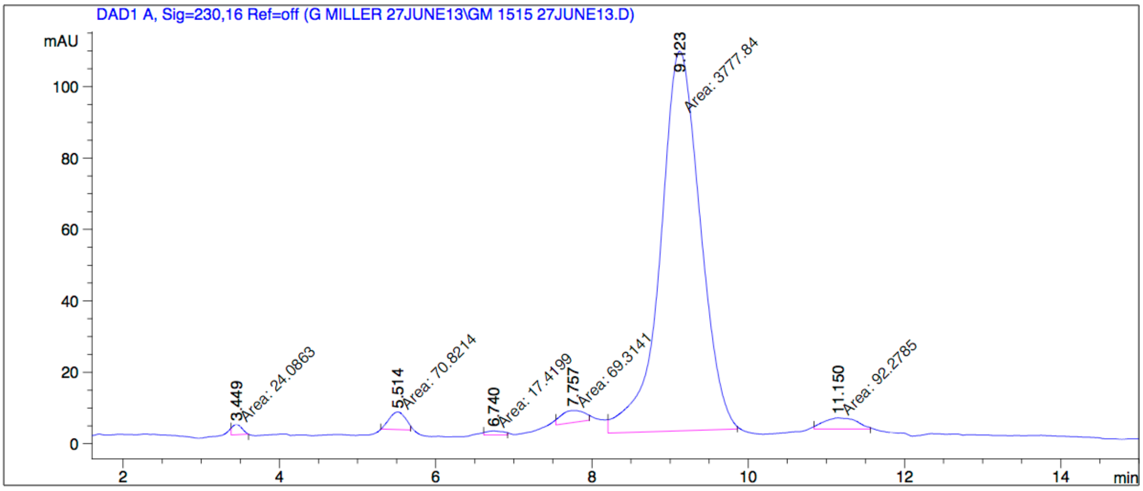


```
    =====================================================================
                         Area Percent Report
    =====================================================================

    Sorted By             :      Signal
    Multiplier            :      1.0000
    Dilution              :      1.0000
    Use Multiplier & Dilution Factor with ISTDs


    Signal 1: DAD1 A, Sig=230,16 Ref=off
```

Peak #	RetTime [min]	Type	Width [min]	Area [mAU*s]	Height [mAU]	Area %
1	3.449	MM	0.1394	24.08633	2.87877	0.5945
2	5.514	MM	0.2394	70.82142	4.93068	1.7479
3	6.740	MM	0.2571	17.41991	1.12947	0.4299
4	7.757	MM	0.3419	69.31406	3.37922	1.7107
5	9.123	MM	0.5920	3777.84351	106.36275	93.2395
6	11.150	MM	0.4915	92.27850	3.12892	2.2775
Totals :				4051.76371	121.80981	

```
    =====================================================================
                          *** End of Report ***
```

Supplementary Figure 25: LCMS for **5**

Display Report

Analysis Info

Analysis Name	GM 1515 27JUNE13.D	Acquisition Date	28/06/2013 14:56:30
Method	GAVIN MILLER 3CM COL 100ACN.M	Operator	mib
Sample Name	GM 1515 27June13	Instrument	LC-MSD-Trap-SL
Comment	Gavin Miller GM 1515 27th June 2013<br>Supelco Ascentis C8 5u 4.6x30mm<br>ESI source +ve ES 230nm 0.5ml/min<br>100ACN only 5ul inj		

Acquisition Parameter

Ion Source Type	ESI	Ion Polarity	Positive	Alternating Ion Polarity	off
Mass Range Mode	Extended	Scan Begin	1000 m/z	Scan End	4000 m/z
Capillary Exit	280.0 Volt	Skim 1	40.0 Volt	Trap Drive	218.9
Accumulation Time	200000 µs	Averages	7 Spectra	Auto MS/MS	off

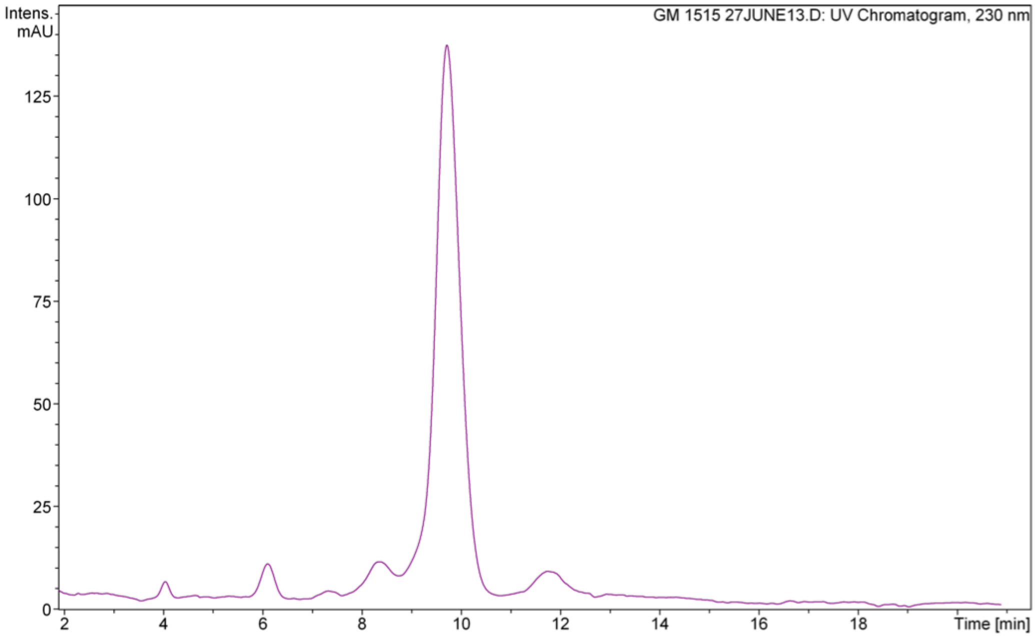

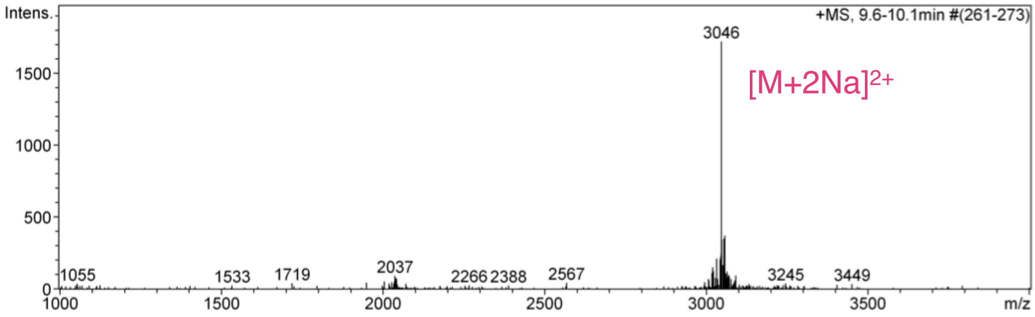

Bruker Daltonics DataAnalysis 3.1 printed: 03/07/2013 09:21:30 Page 1 of 1

Supplementary Figure 26: ^{1}H NMR (800 MHz; $CDCl_3$) spectrum form **6**

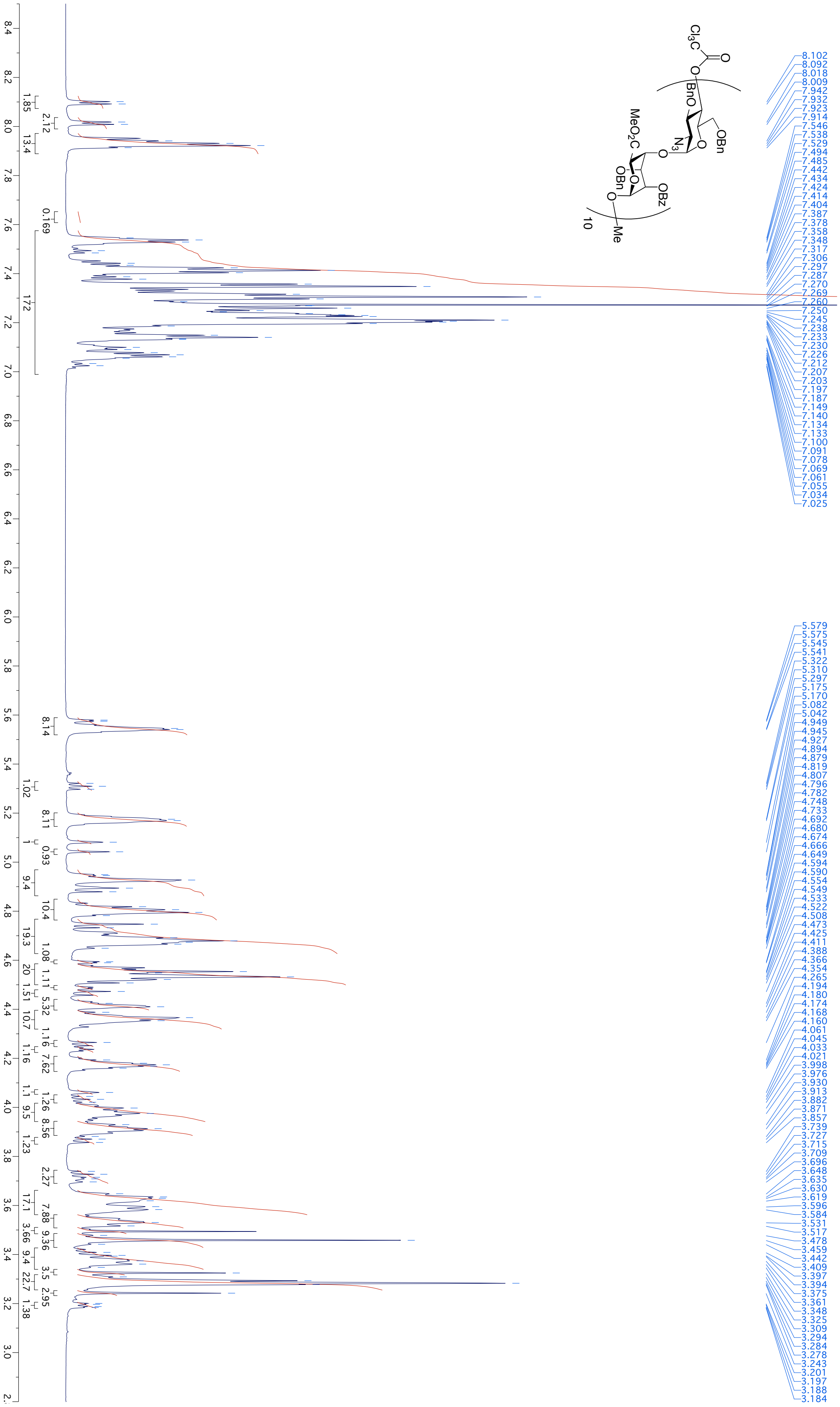

Supplementary Figure 27: ^{1}H NMR (800 MHz; $CDCl_3$) expansion for **6**

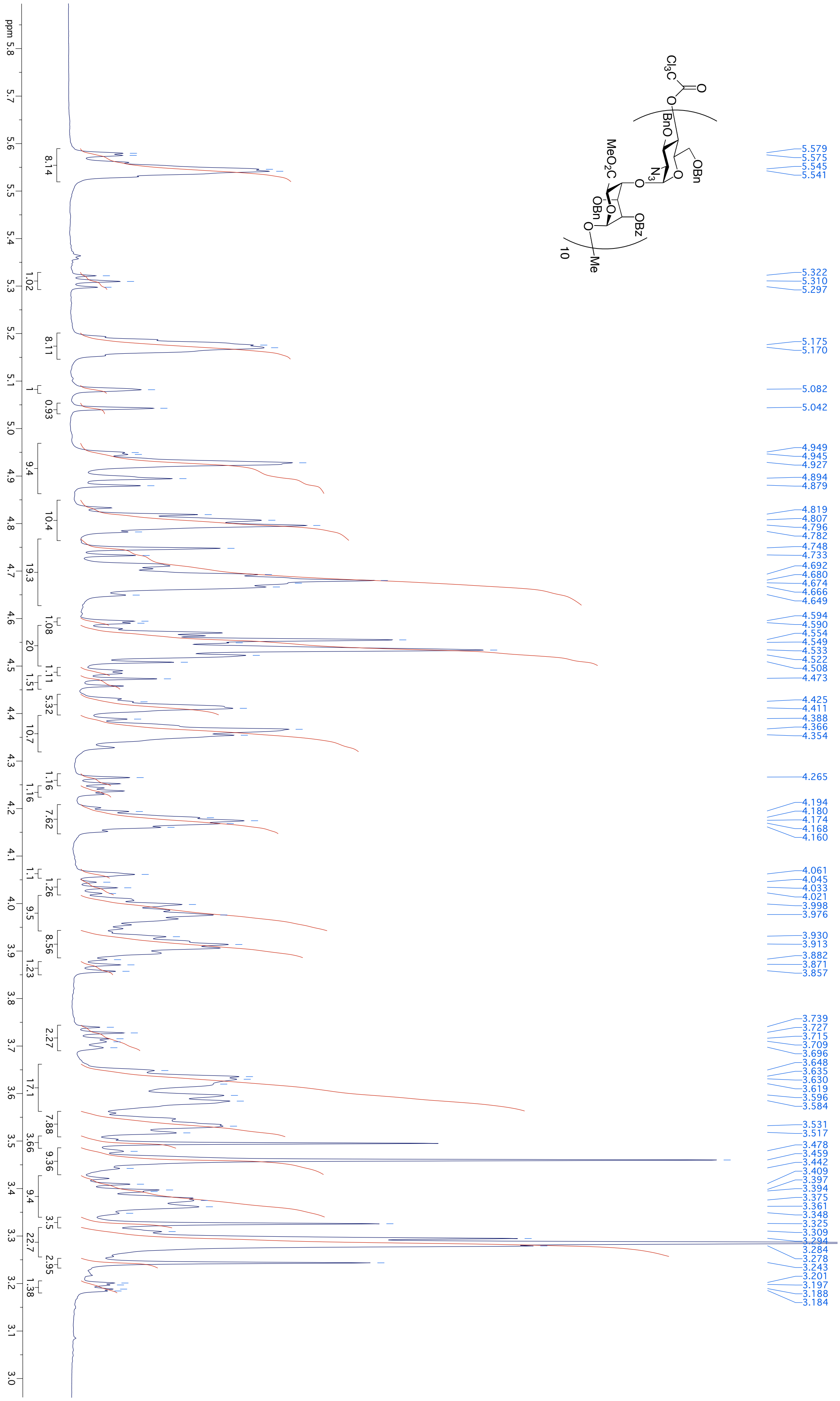

Supplementary Figure 28: HSQC NMR (800 MHz; $CDCl_3$) spectrum for **6**

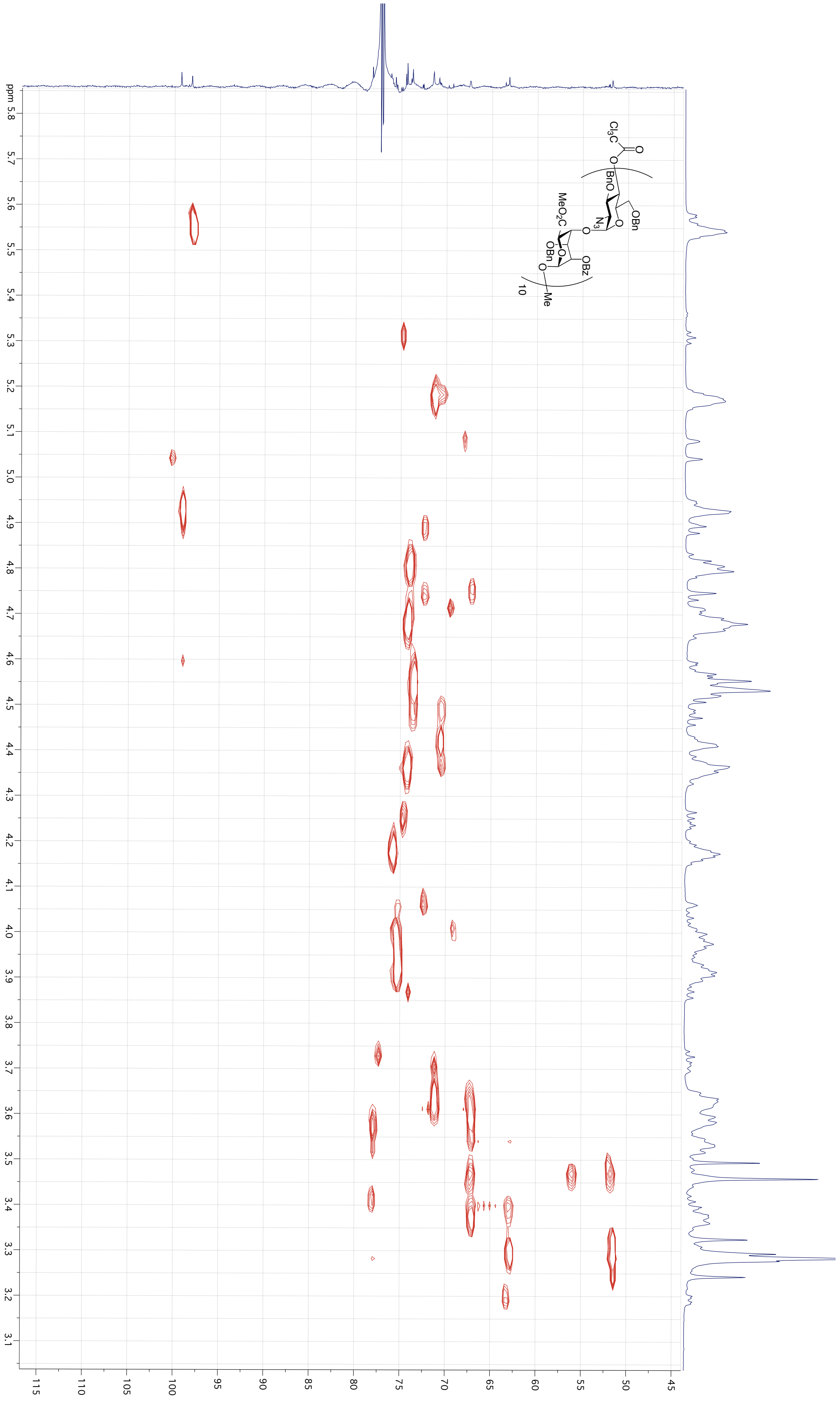

Supplementary Figure 29: MALDI MS and isotope pattern for **6**

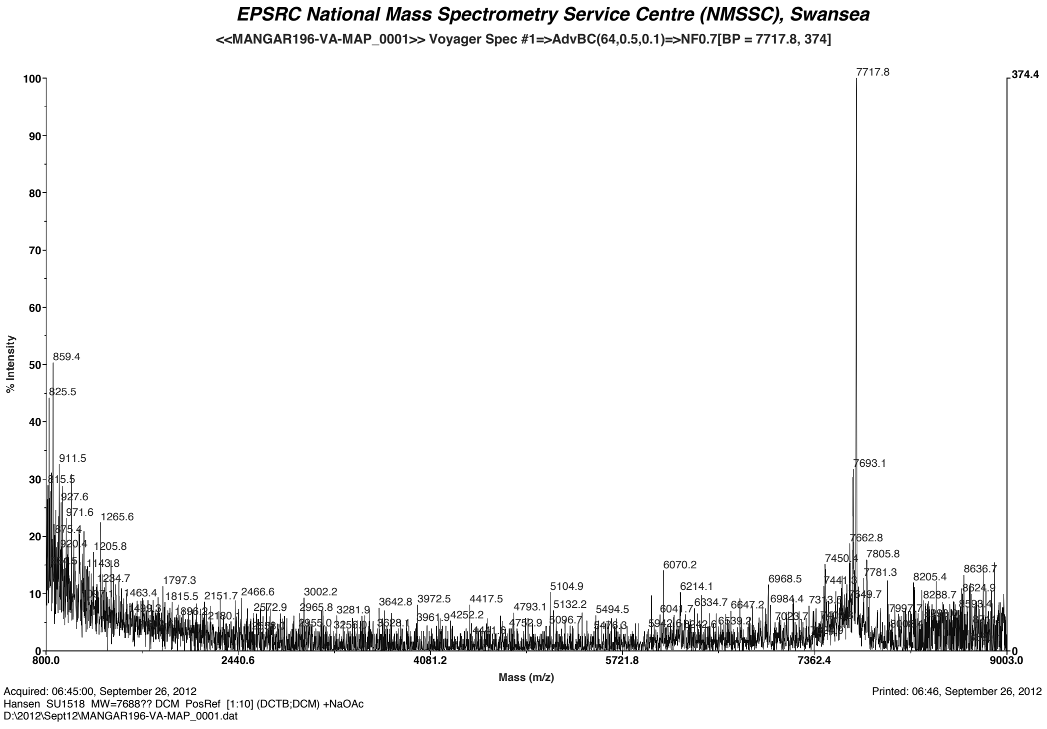

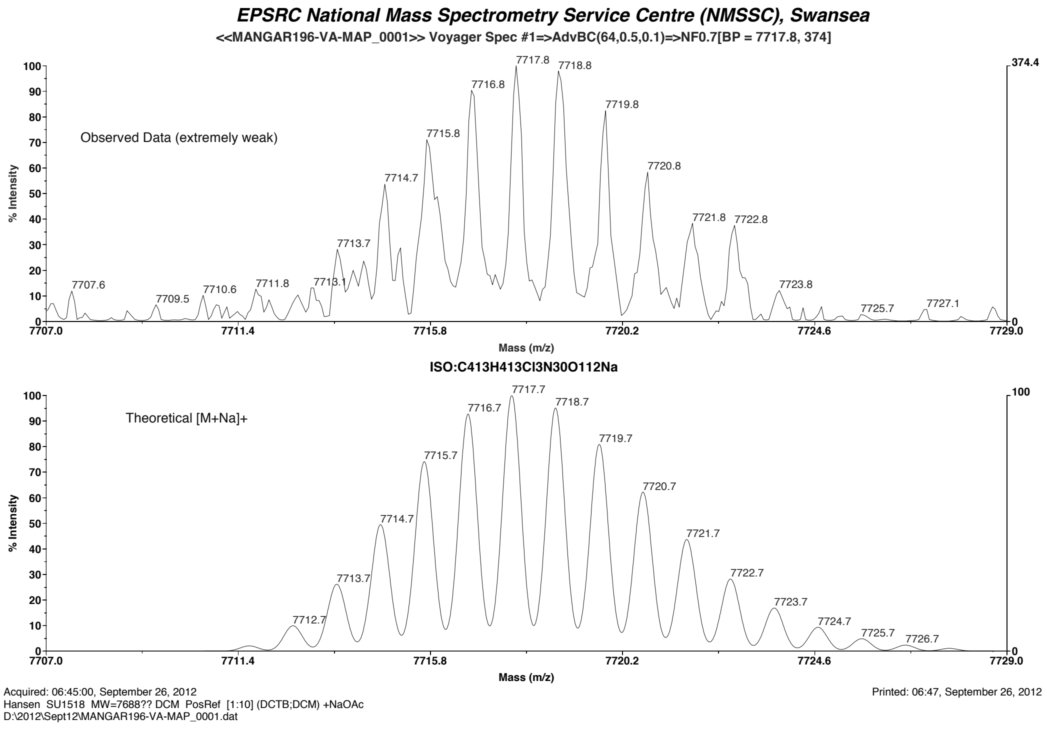

Supplementary Figure 30: ^{1}H NMR (400 MHz; $CDCl_3$) spectrum for **7**

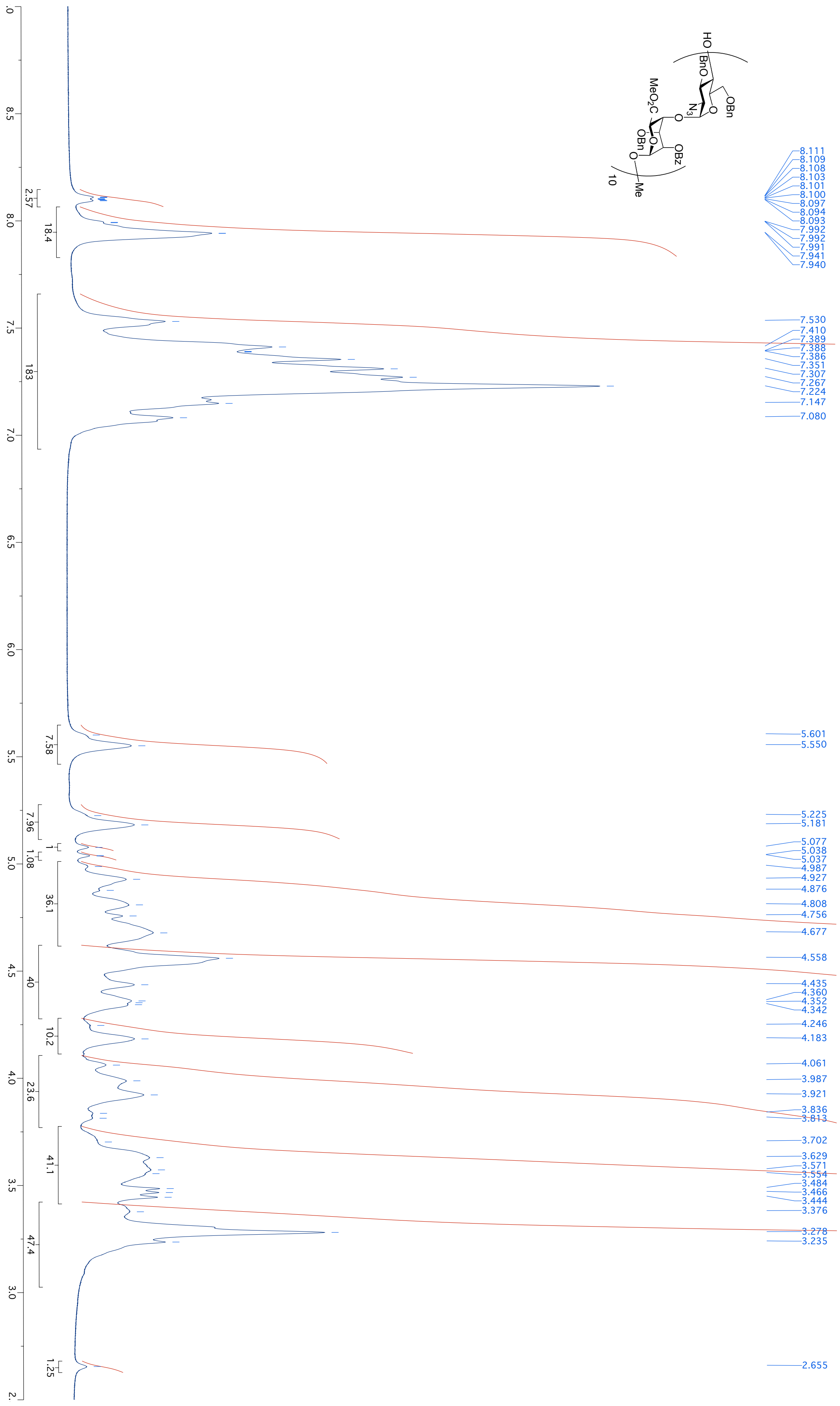

Supplementary Figure 31: COSY NMR (400 MHz; $CDCl_3$) spectrum for **7**

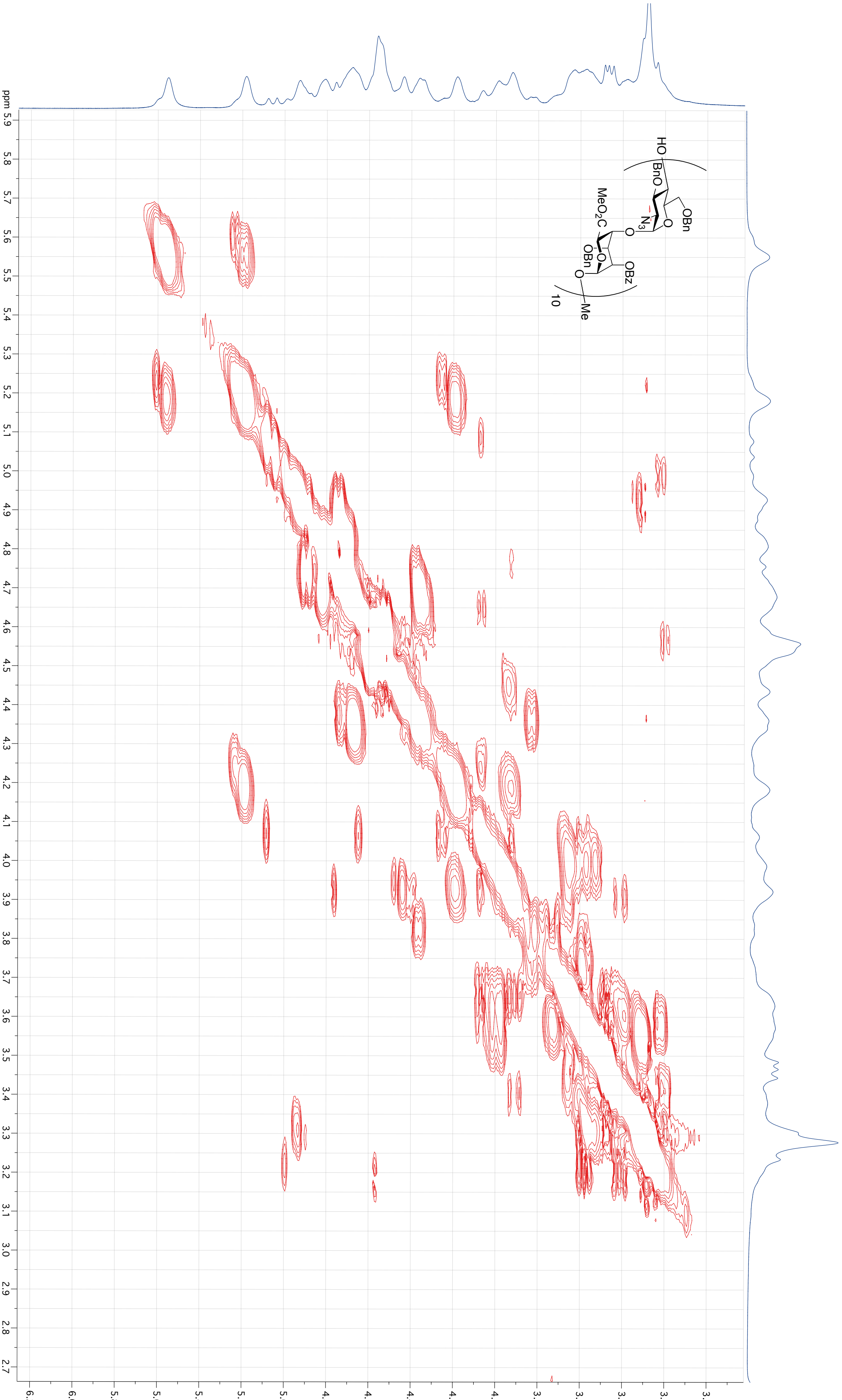

Supplementary Figure 31: HSQC NMR (400 MHz; $CDCl_3$) spectrum for **7**

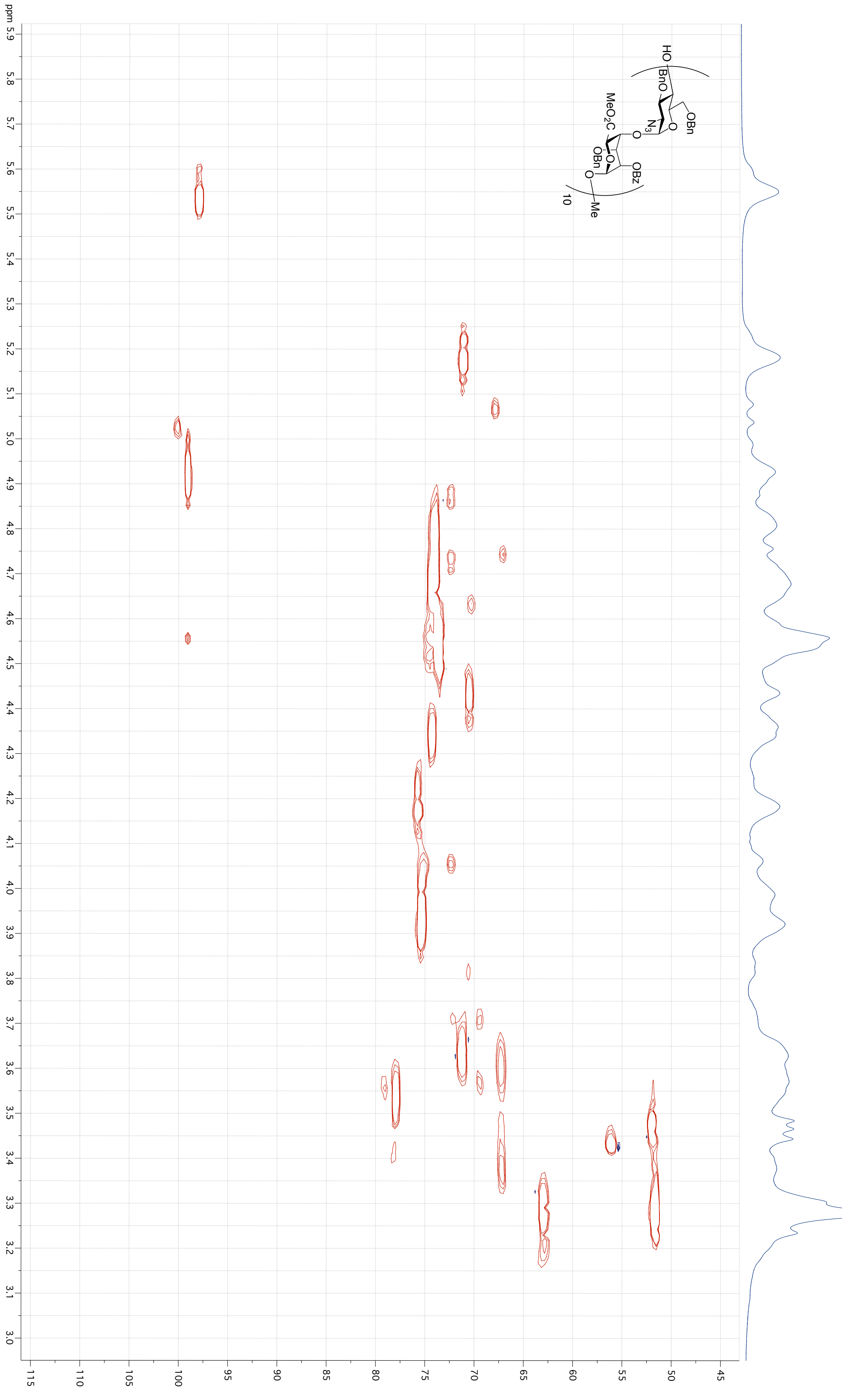

Supplementary Figure 32: MALDI MS and isotope pattern for **7**

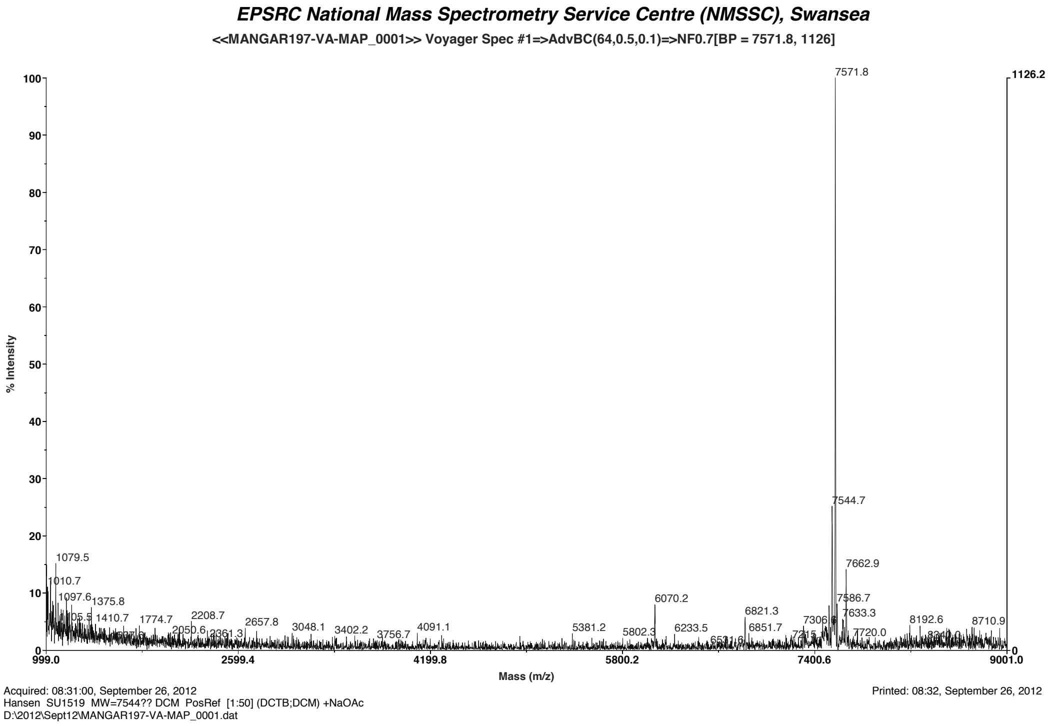

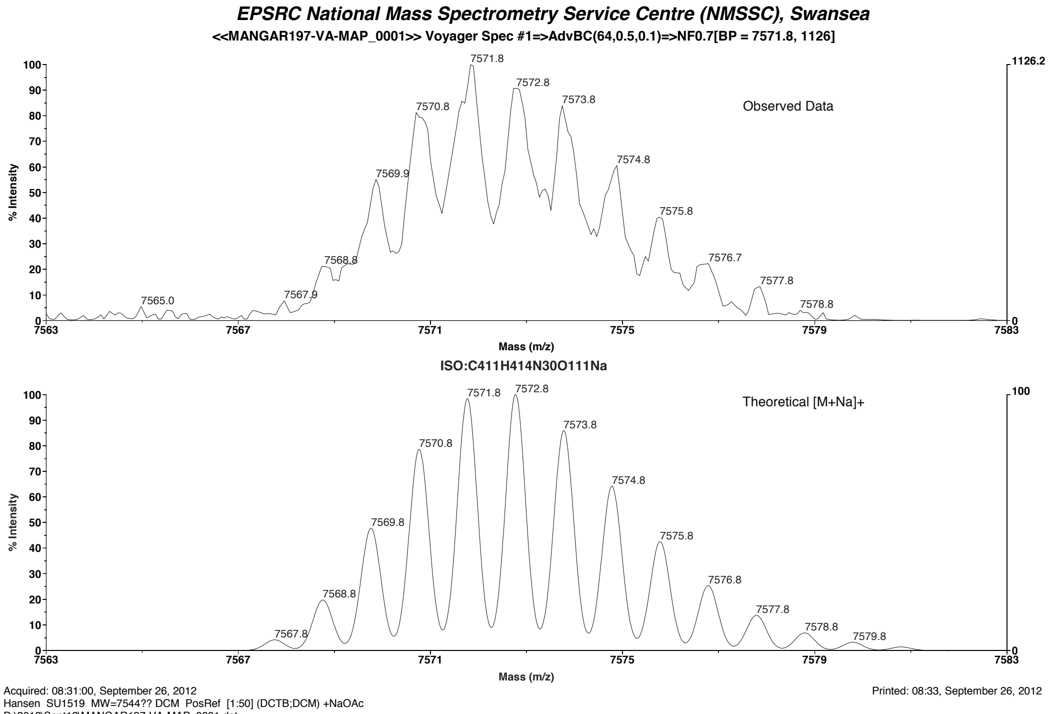

Supplementary Figure 33: ^{1}H NMR (400 MHz; $CDCl_3$) spectrum for **8**

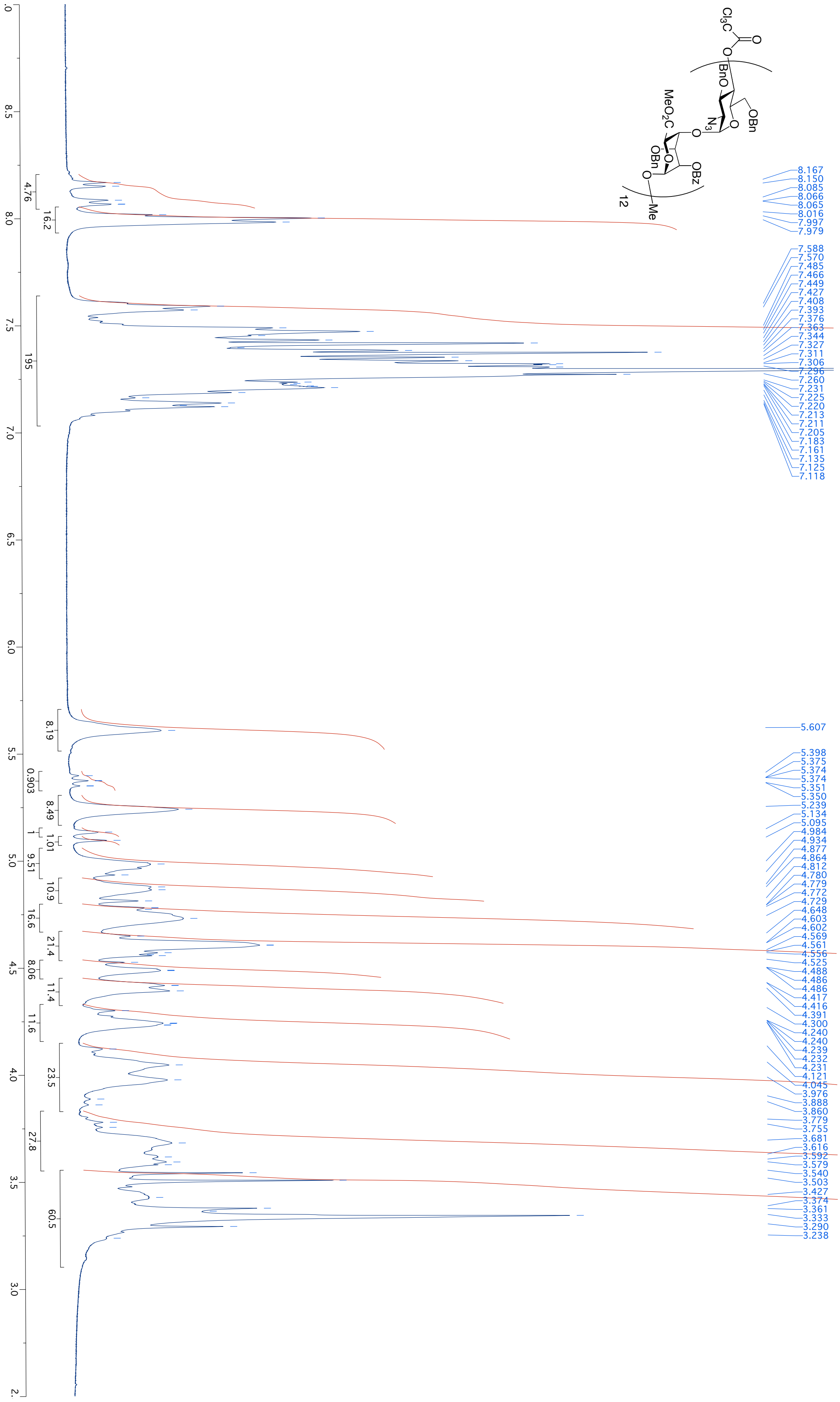

Supplementary Figure 34: COSY NMR (400 MHz; $CDCl_3$) spectrum for **8**

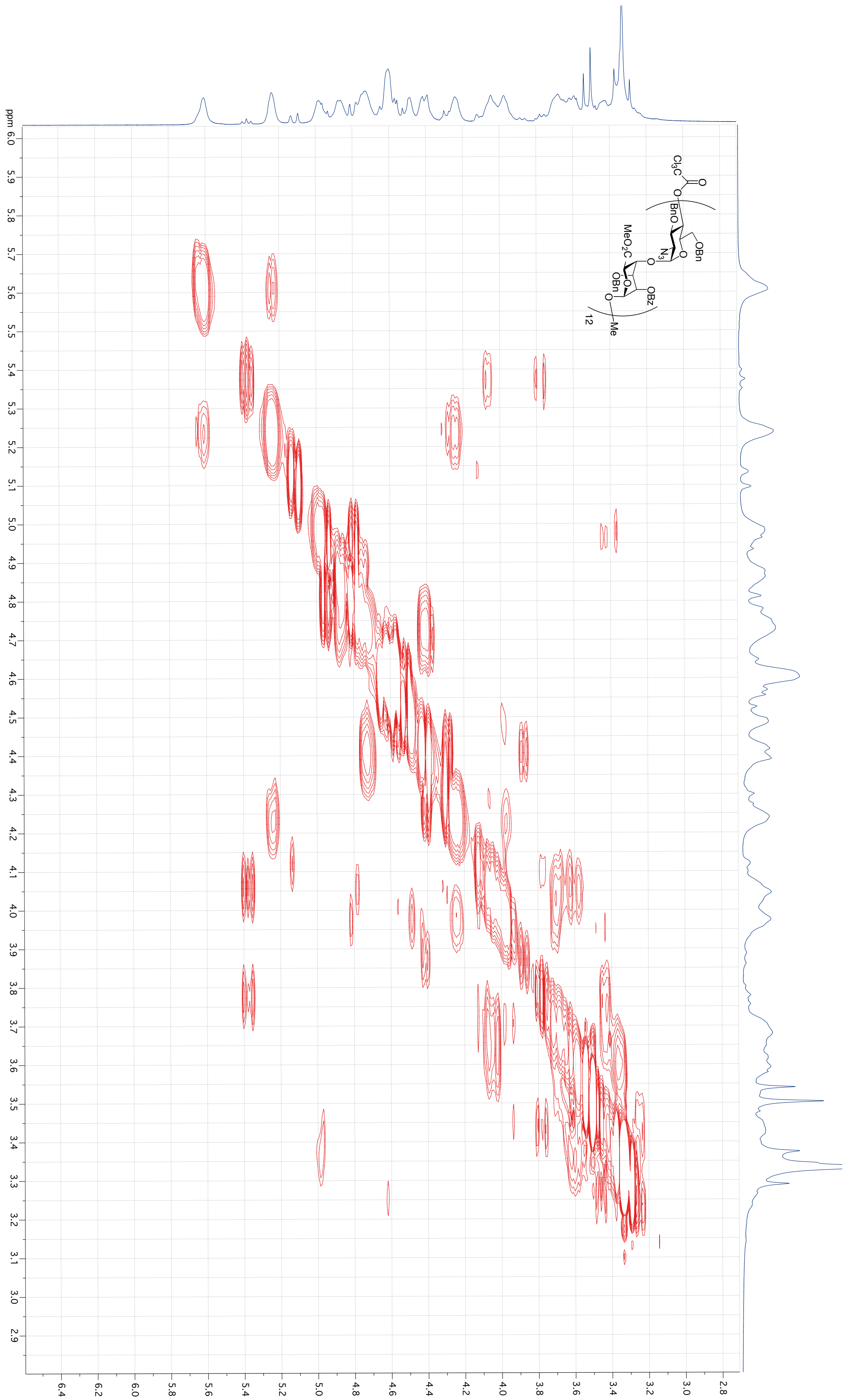

Supplementary Figure 35: HSQC NMR (400 MHz; $CDCl_3$) spectrum for **8**

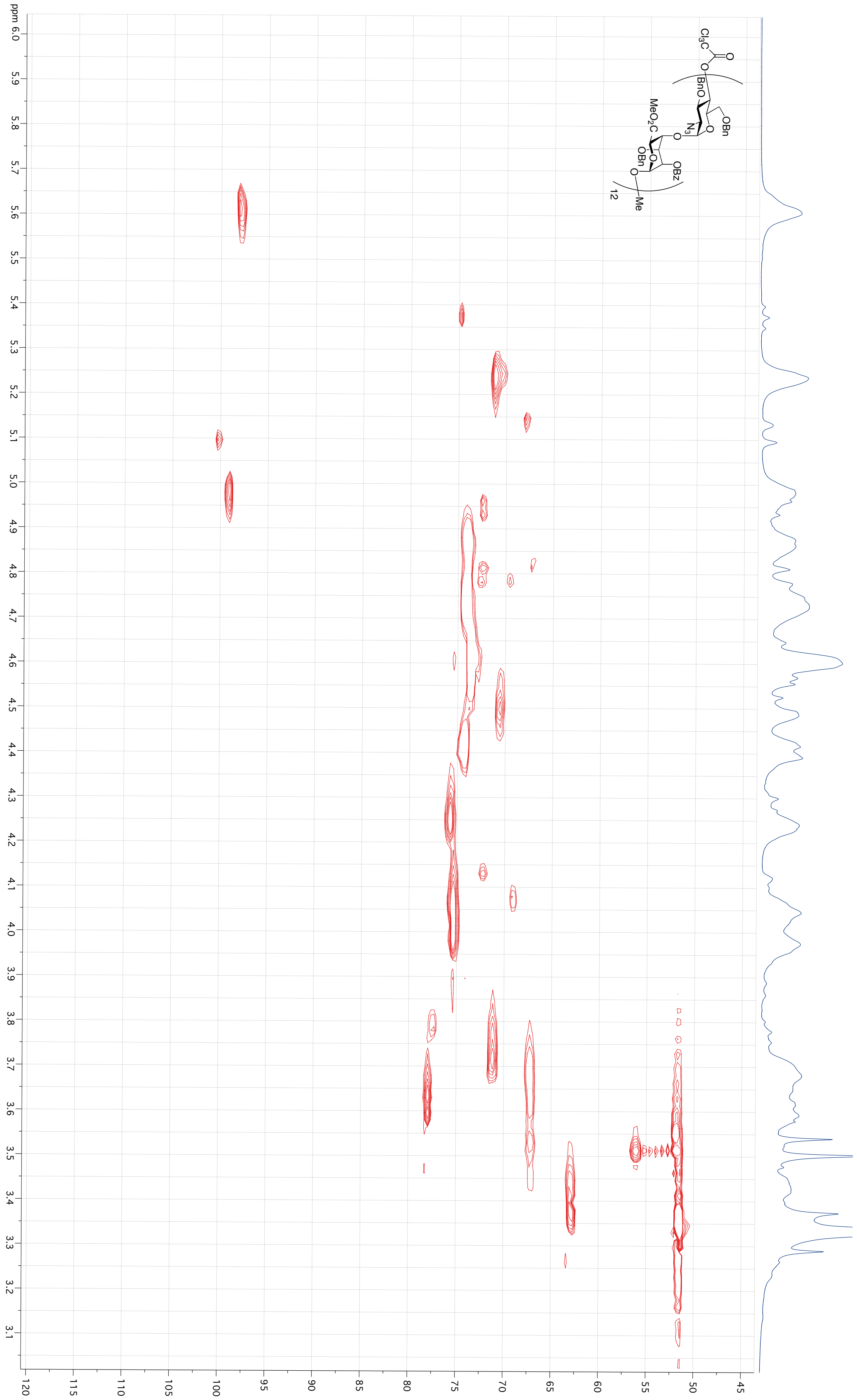

Supplementary Figure 36: MALDI MS spectrum for **8**

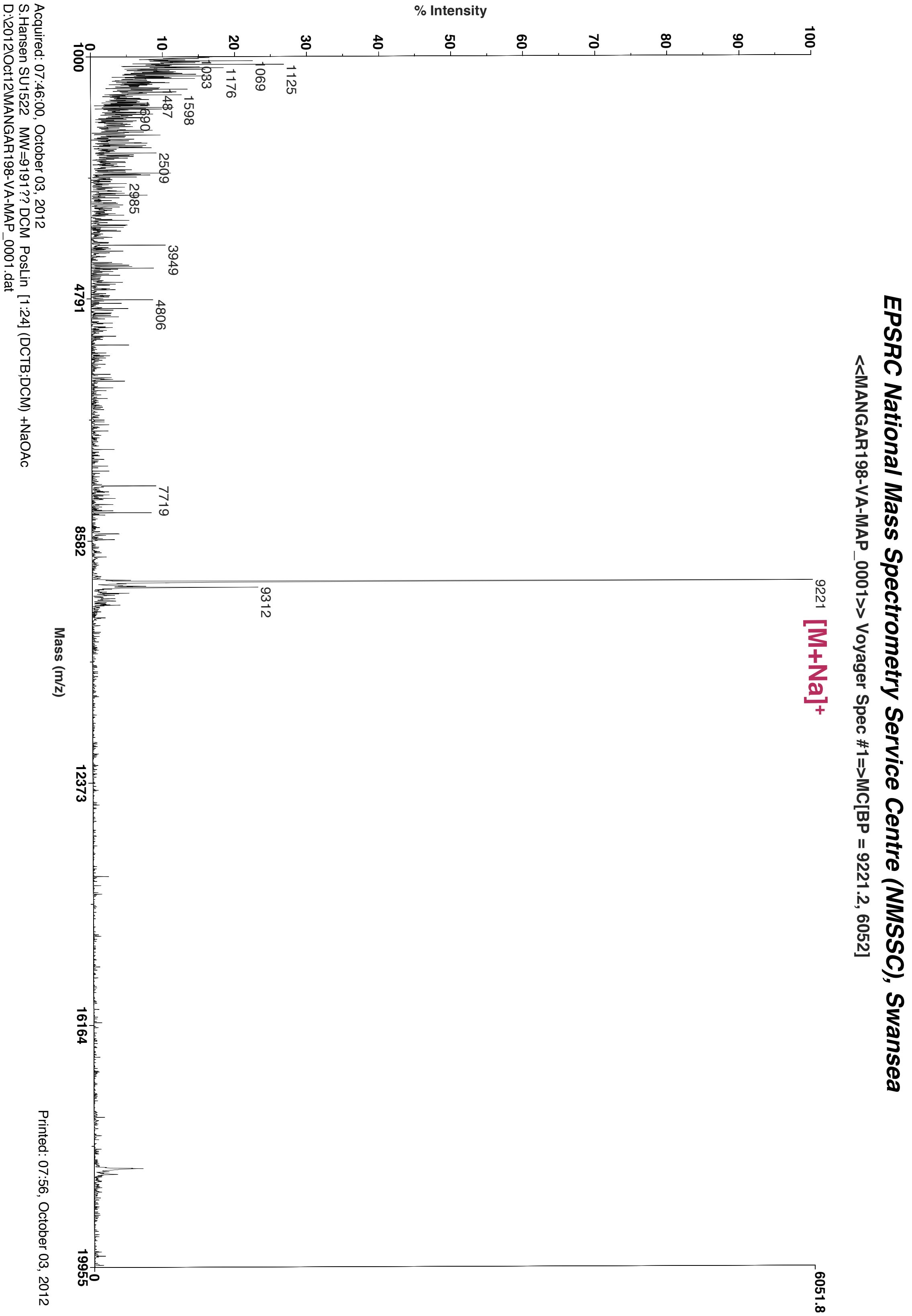

Supplementary Figure 37: ^{1}H NMR (400 MHz; $CDCl_3$) spectrum for **9**

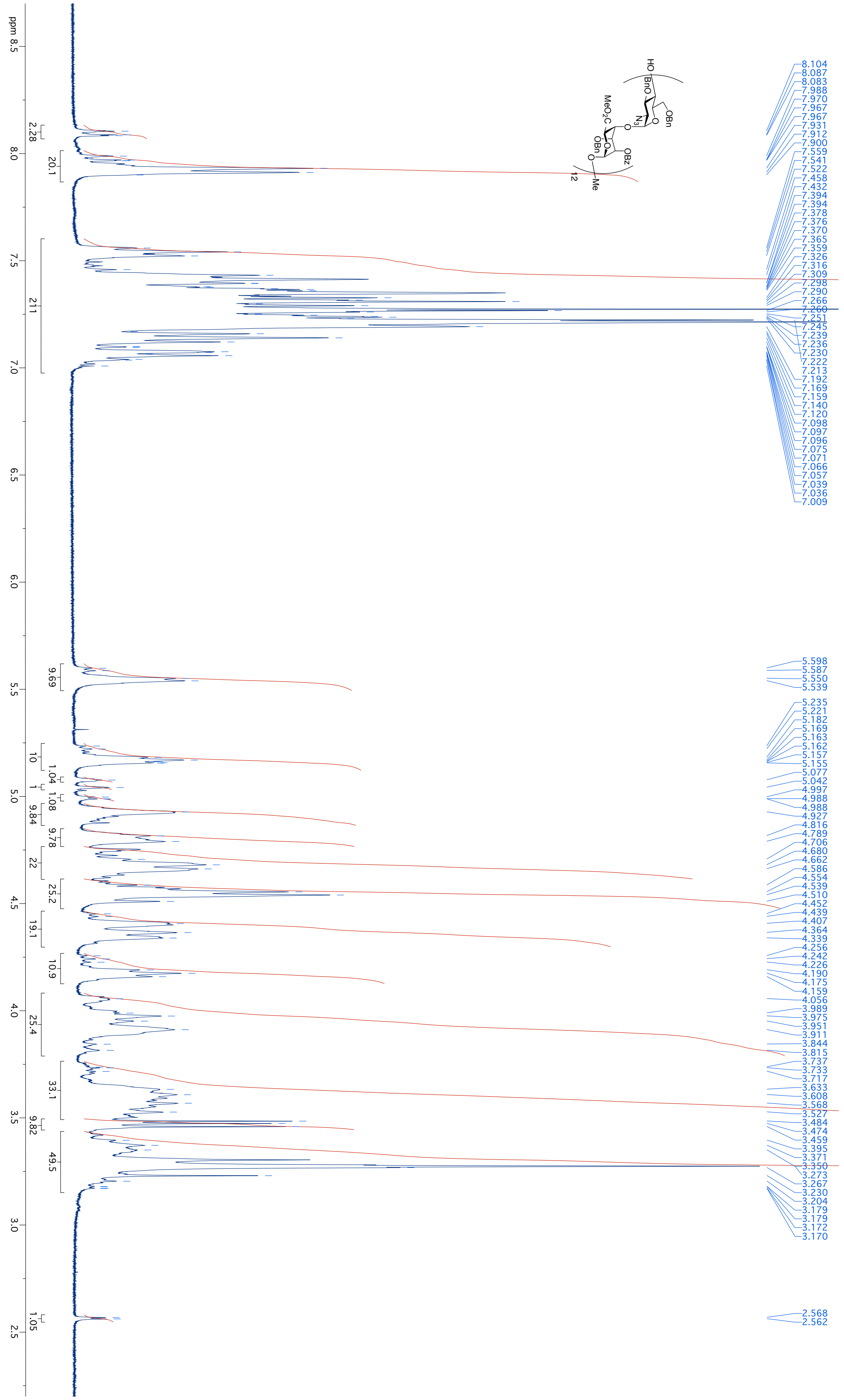

Supplementary Figure 38: COSY NMR (400 MHz; $CDCl_3$) spectrum for **9**

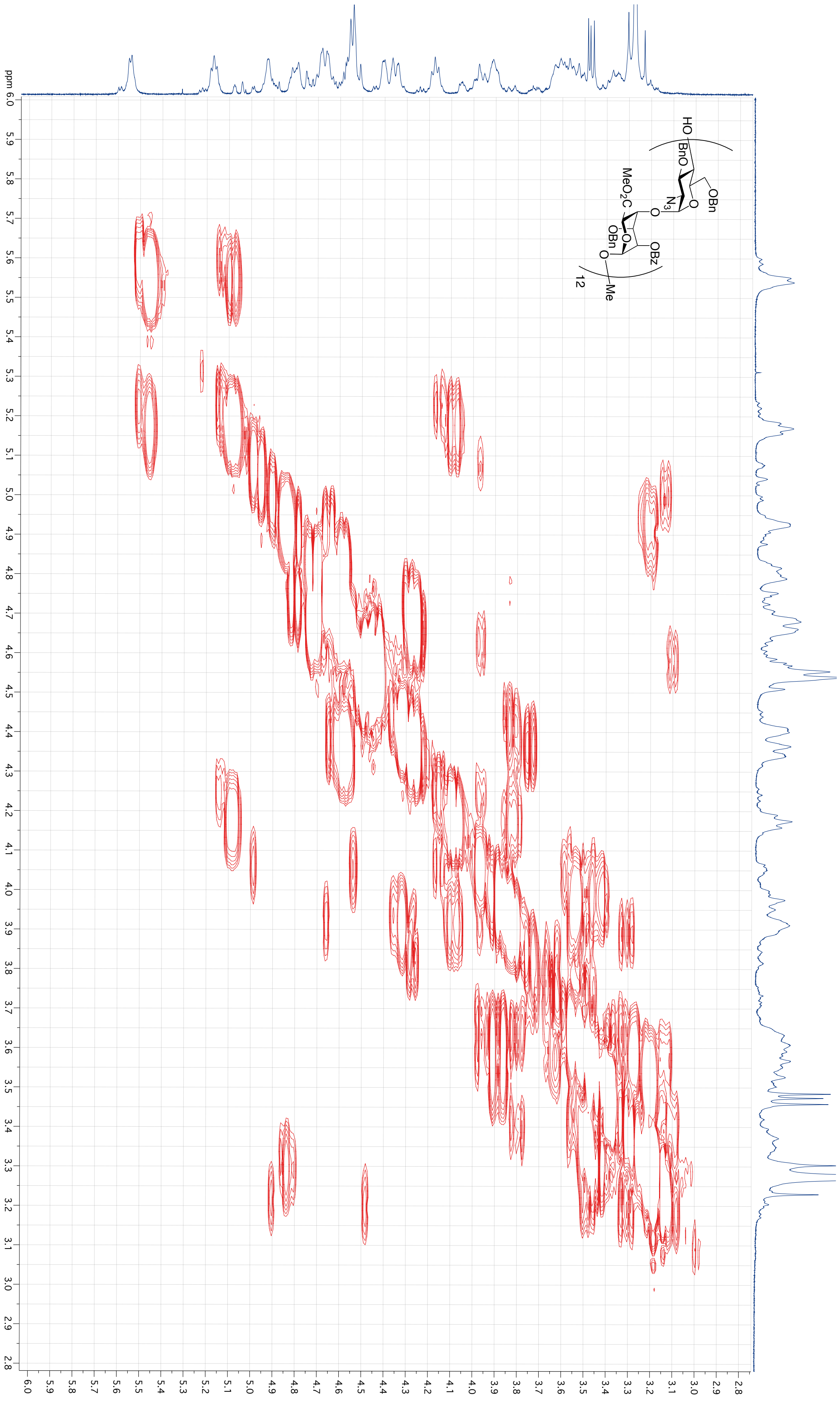

Supplementary Figure 39: MALDI MS spectrum for **9**

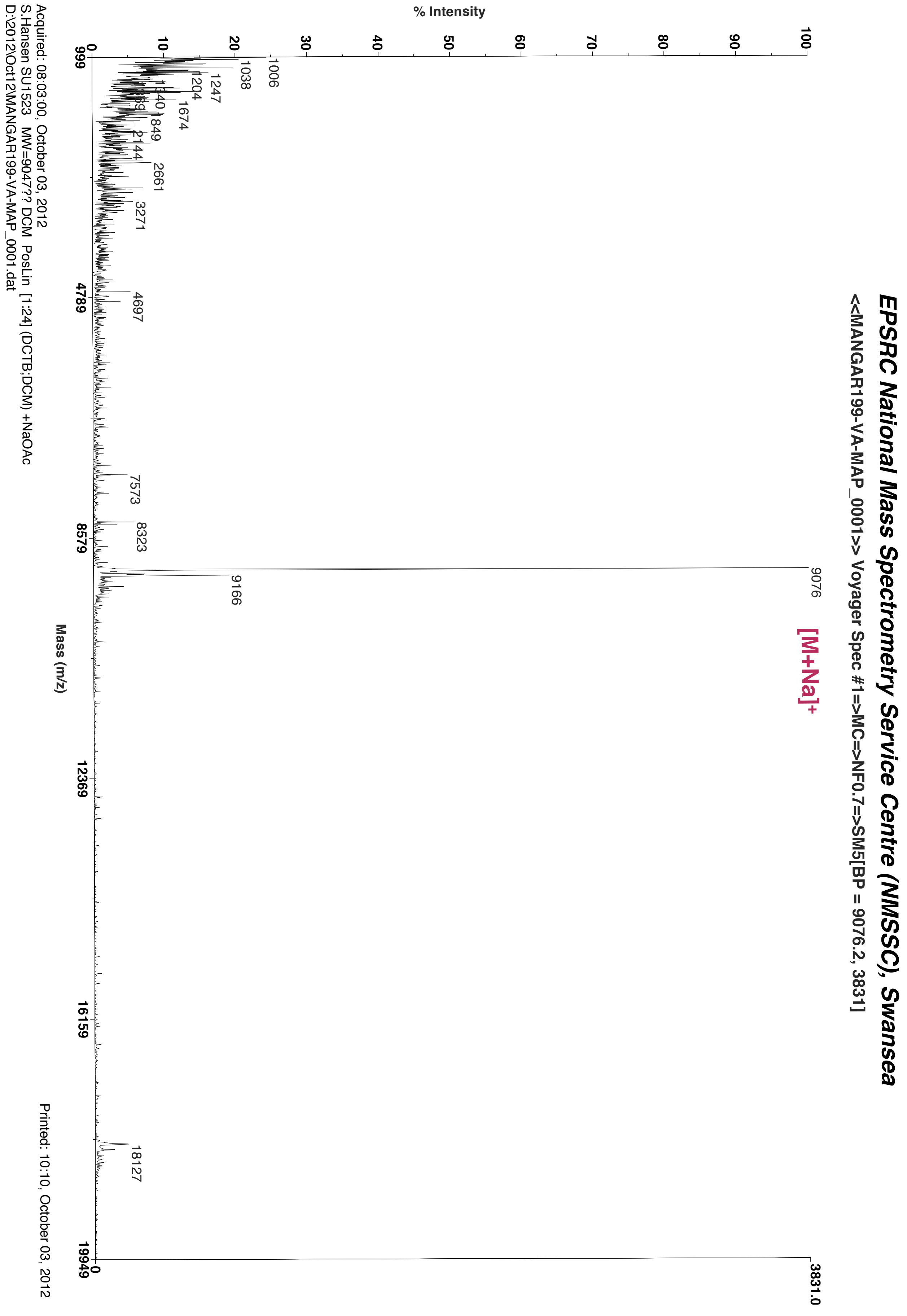

Supplementary Figure 40: ^{1}H NMR (400 MHz; $CDCl_3$) spectrum for **10**

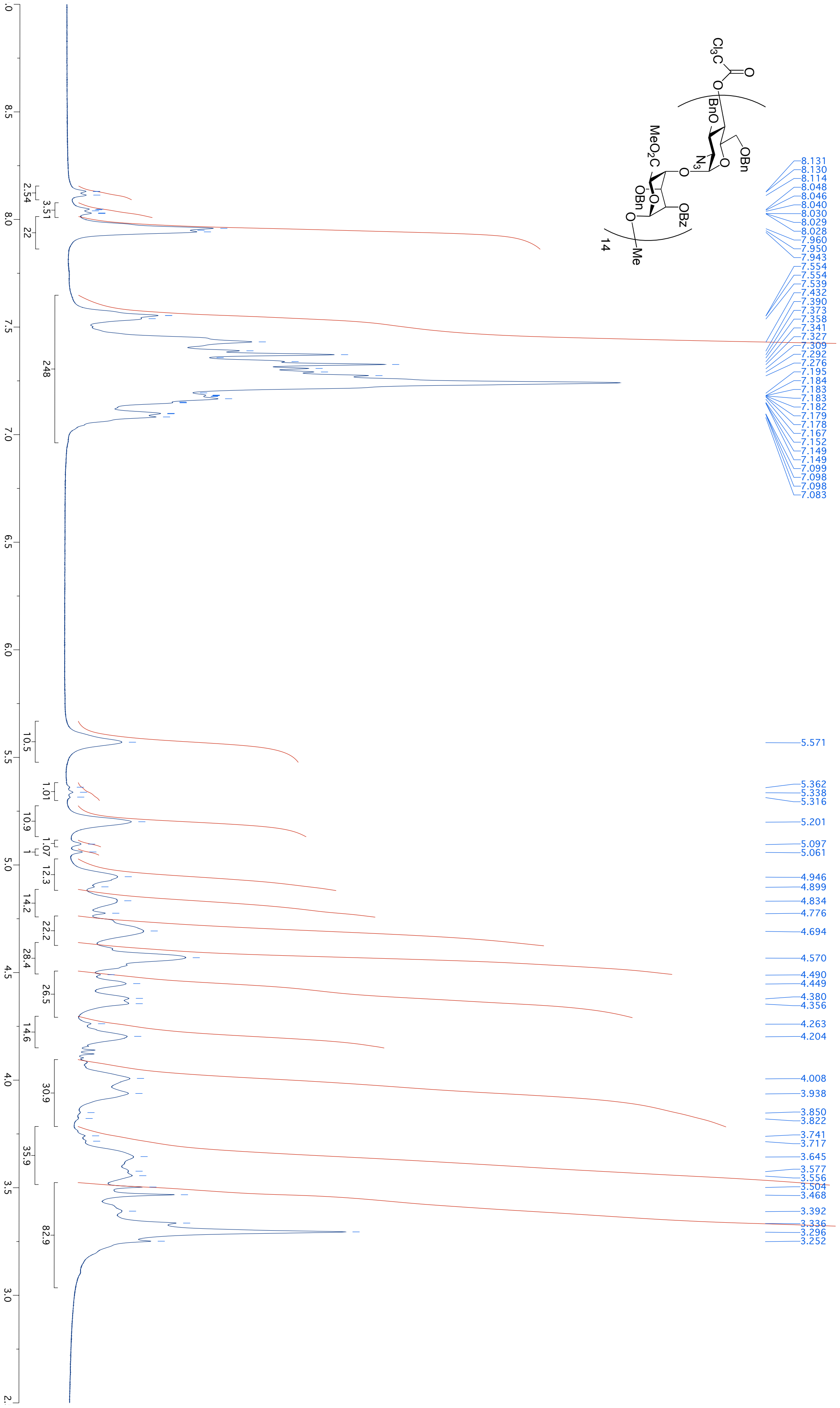

Supplementary Figure 41: COSY NMR (400 MHz; $CDCl_3$) spectrum for **9**

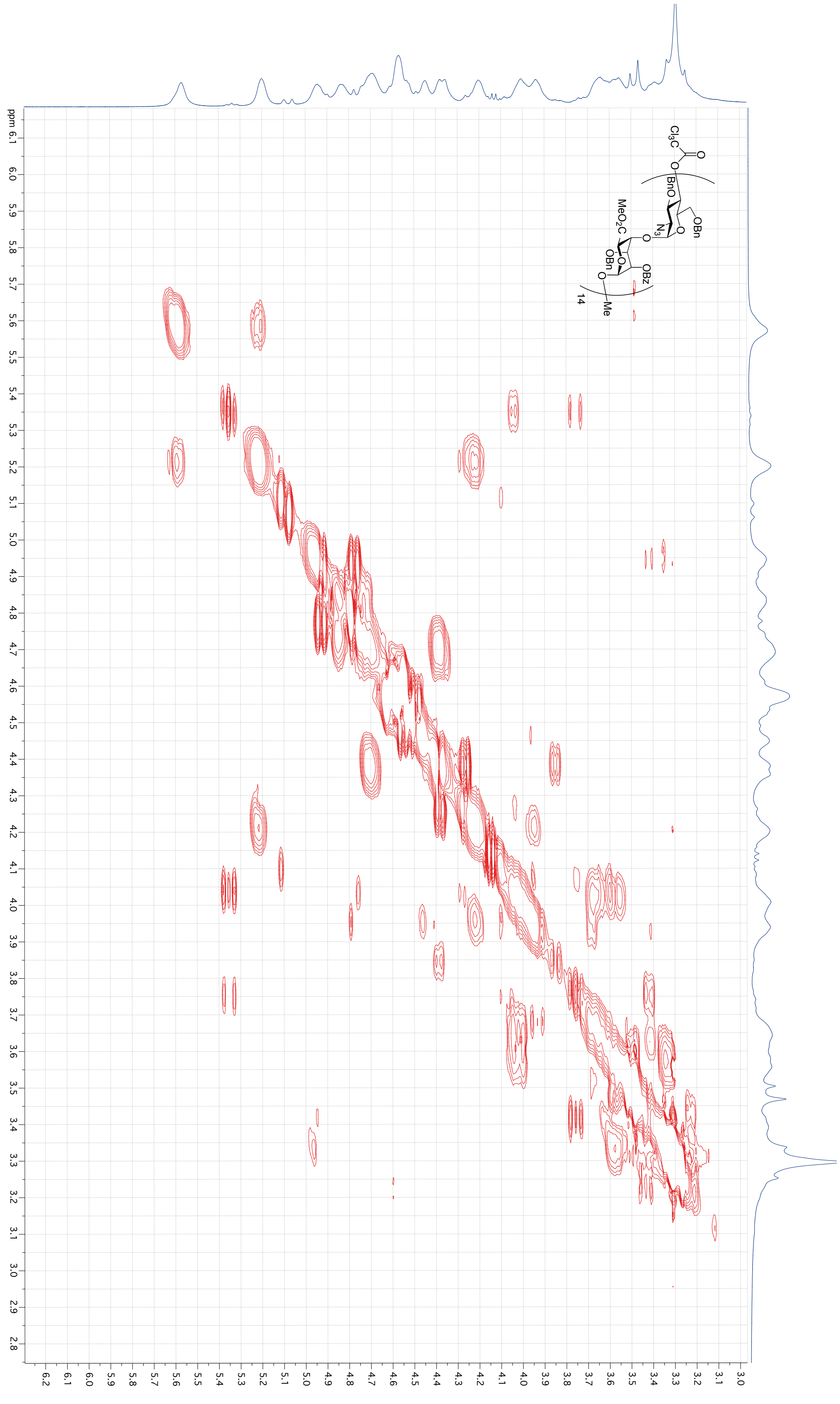

Supplementary Figure 42: HSQC NMR (400 MHz; $CDCl_3$) spectrum for **10**

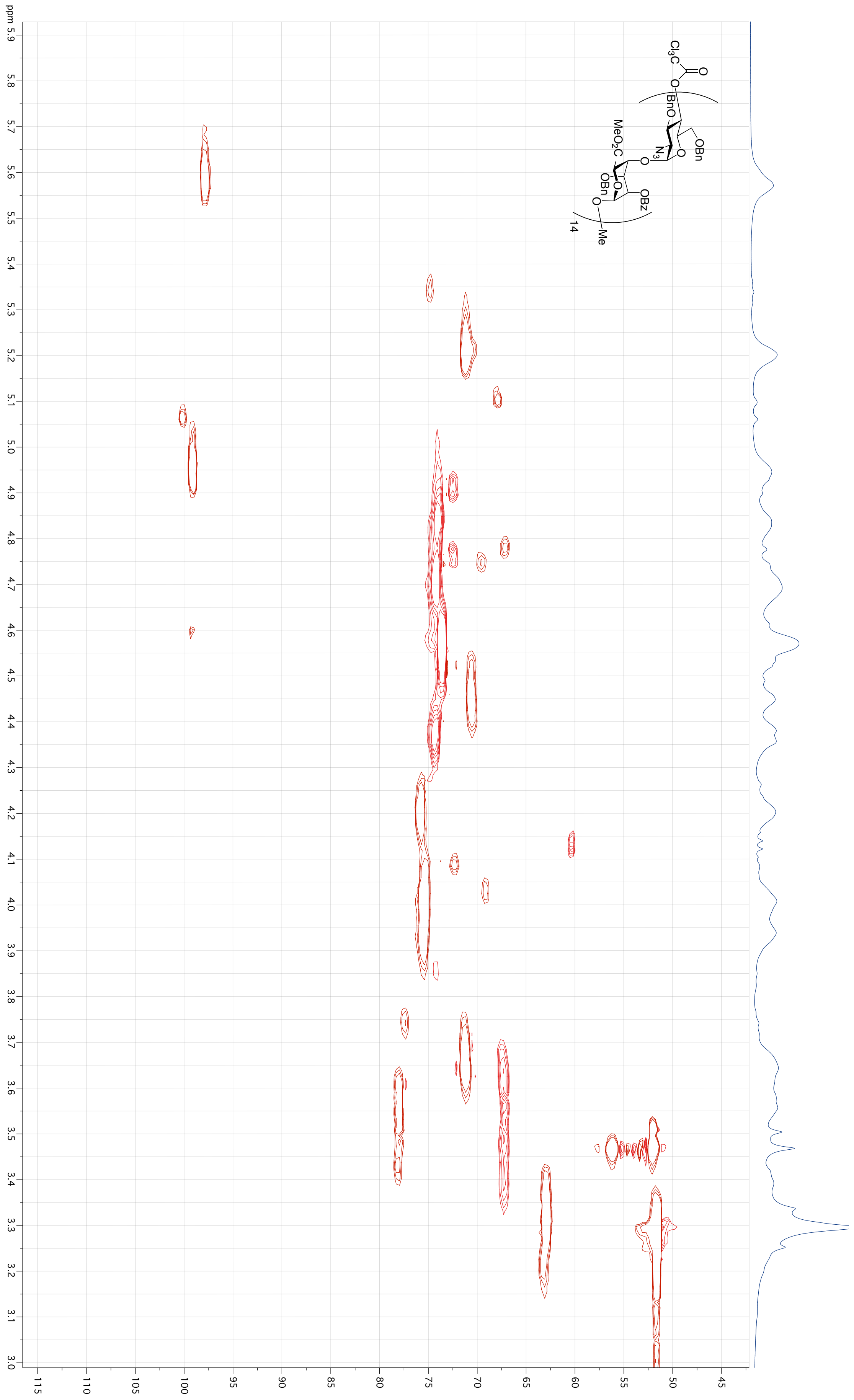

Supplementary Figure 43: MALDI MS spectrum for **10**

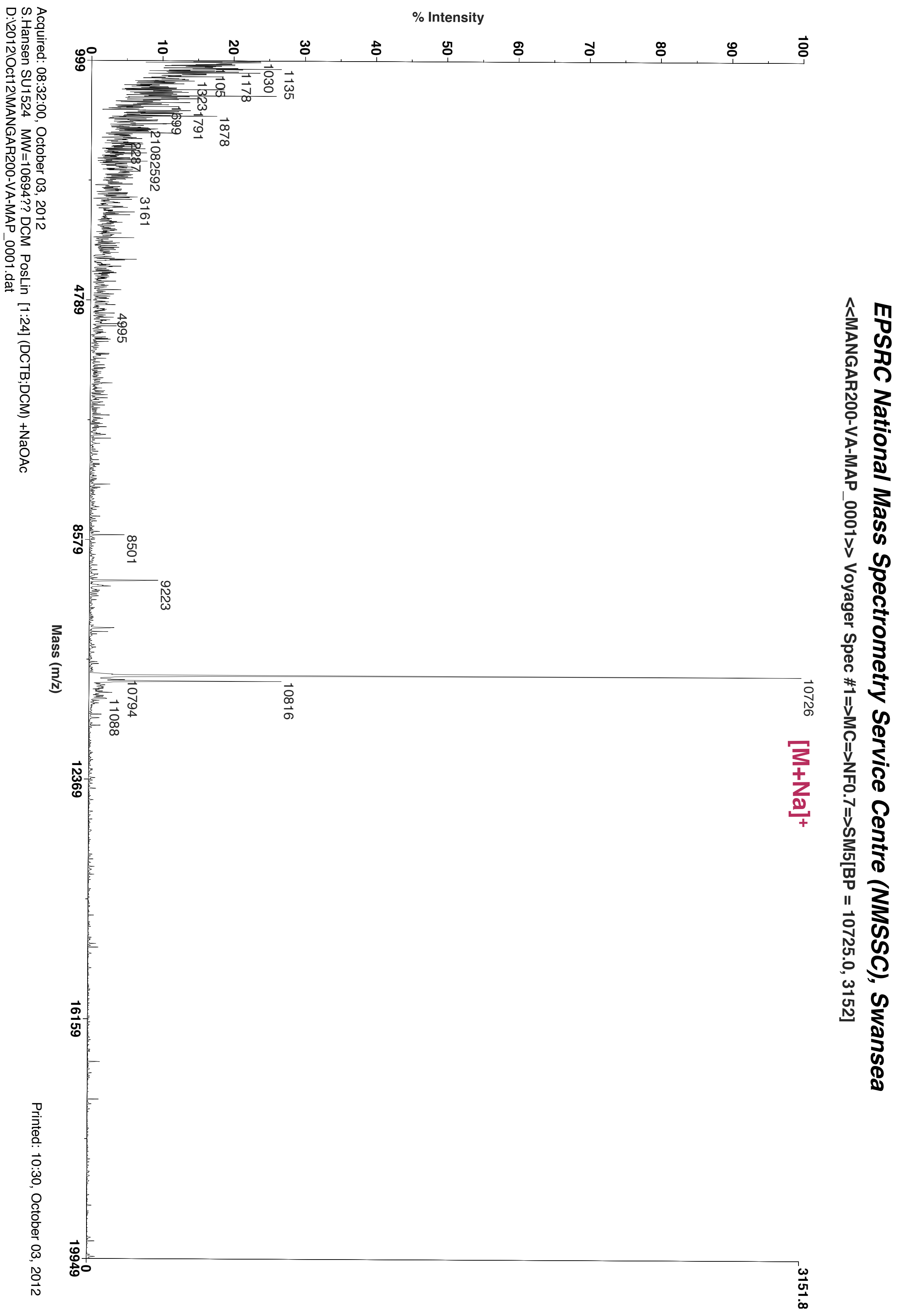

Supplementary Figure 44: ^{1}H NMR (400 MHz; $CDCl_3$) spectrum for **11**

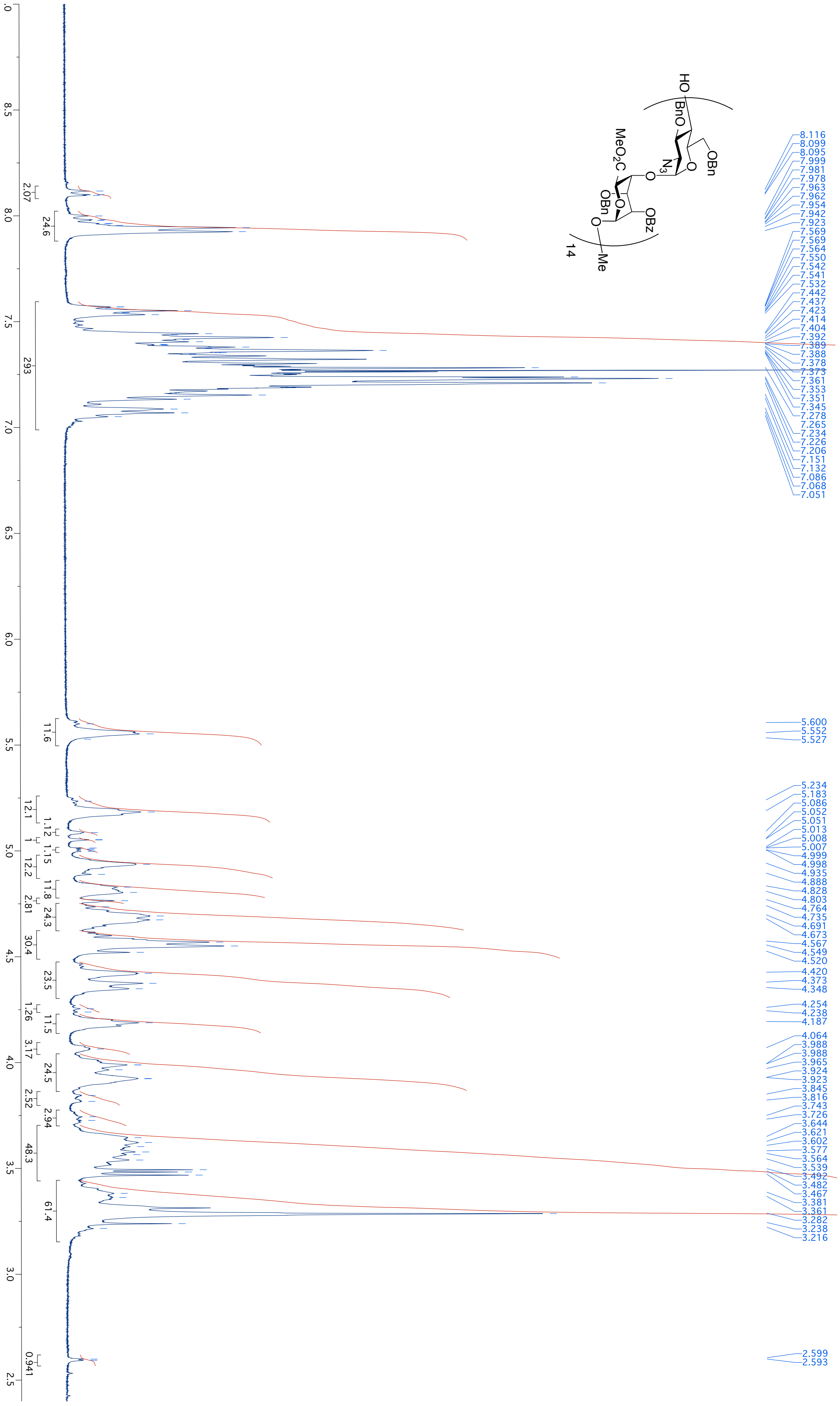

Supplementary Figure 45: COSY NMR (400 MHz; $CDCl_3$) spectrum for **11**

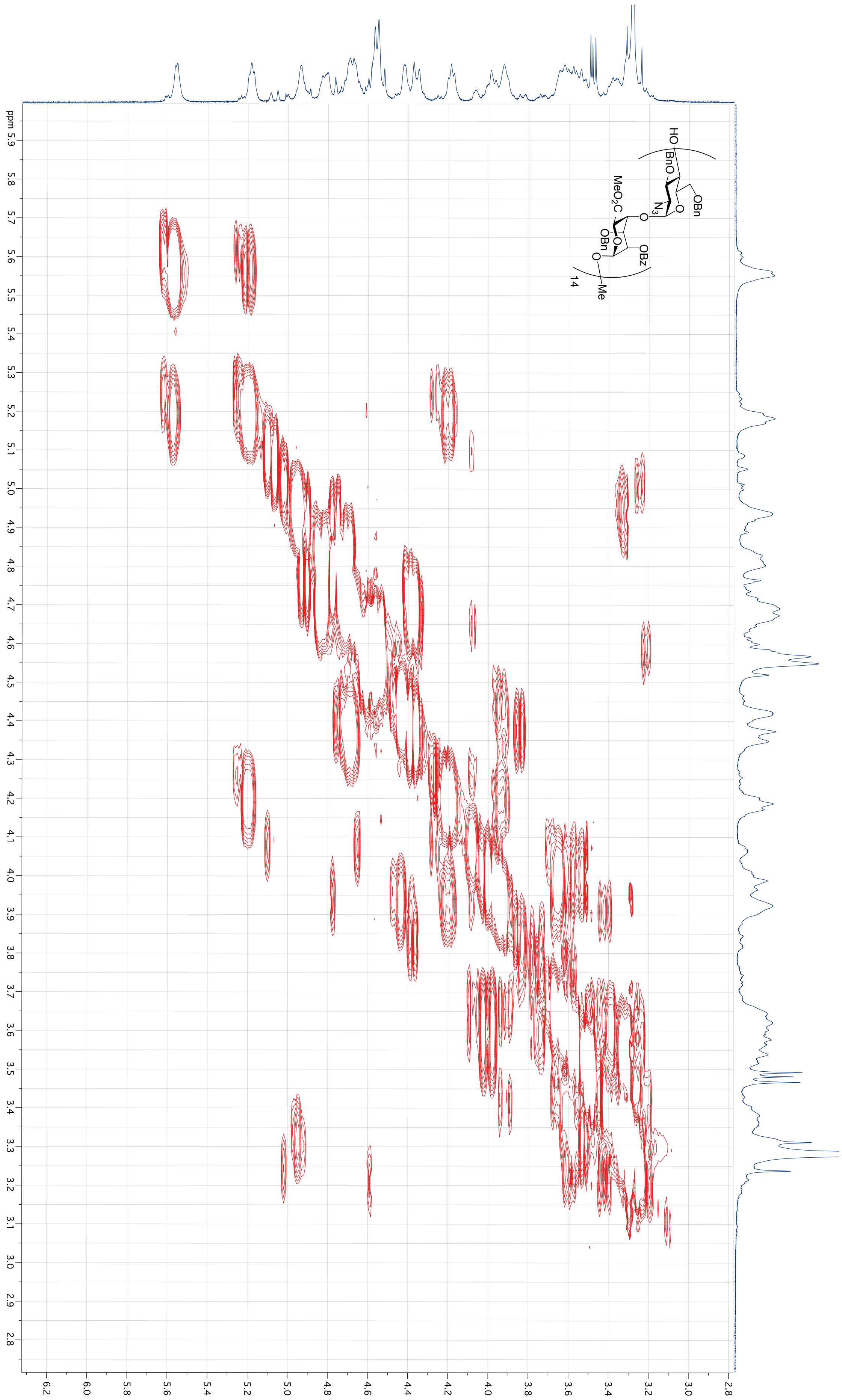

Supplementary Figure 46: MALDI MS spectrum for **11**

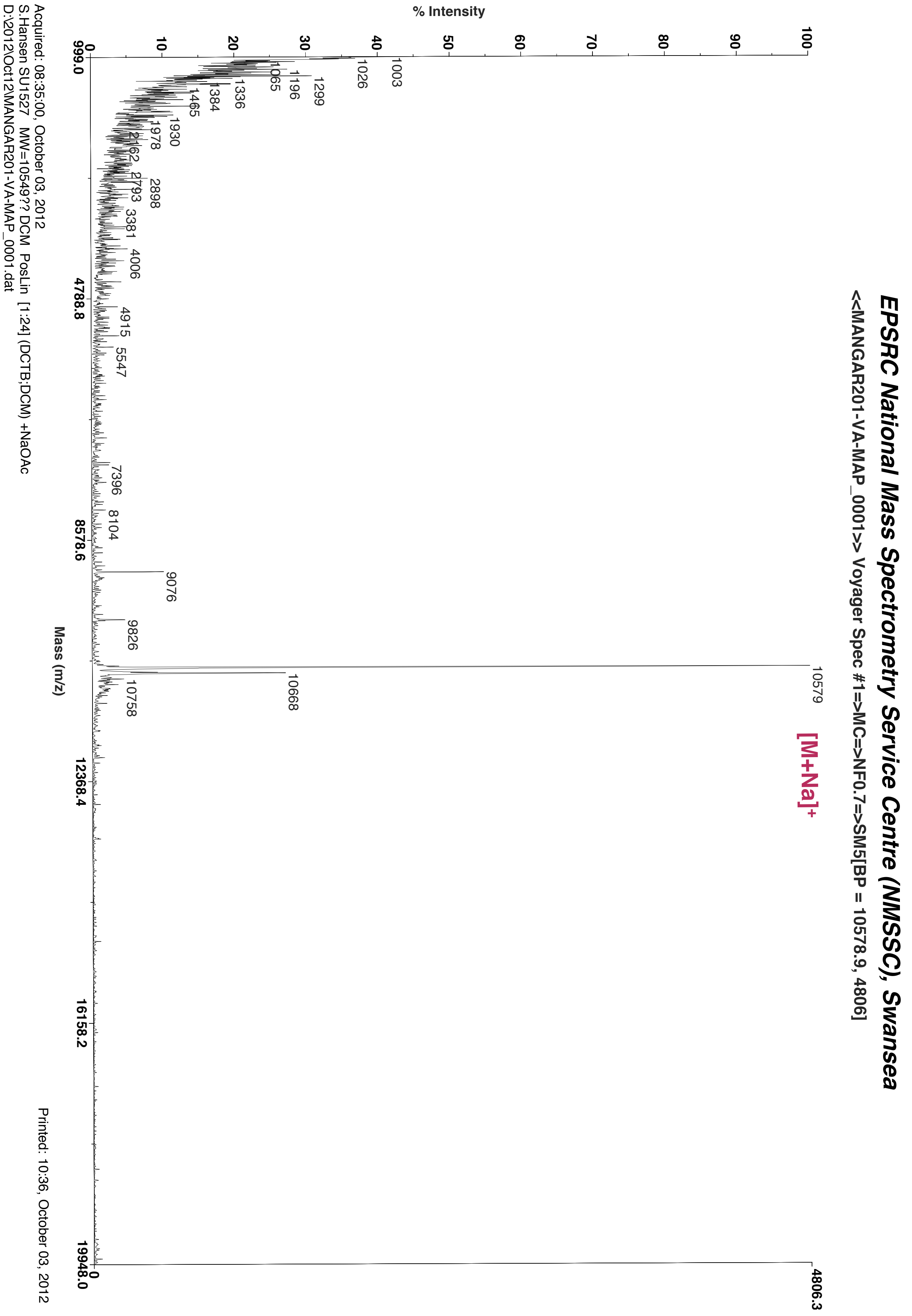

Supplementary Figure 47: ^{1}H NMR (400 MHz; $CDCl_3$) spectrum for **12**

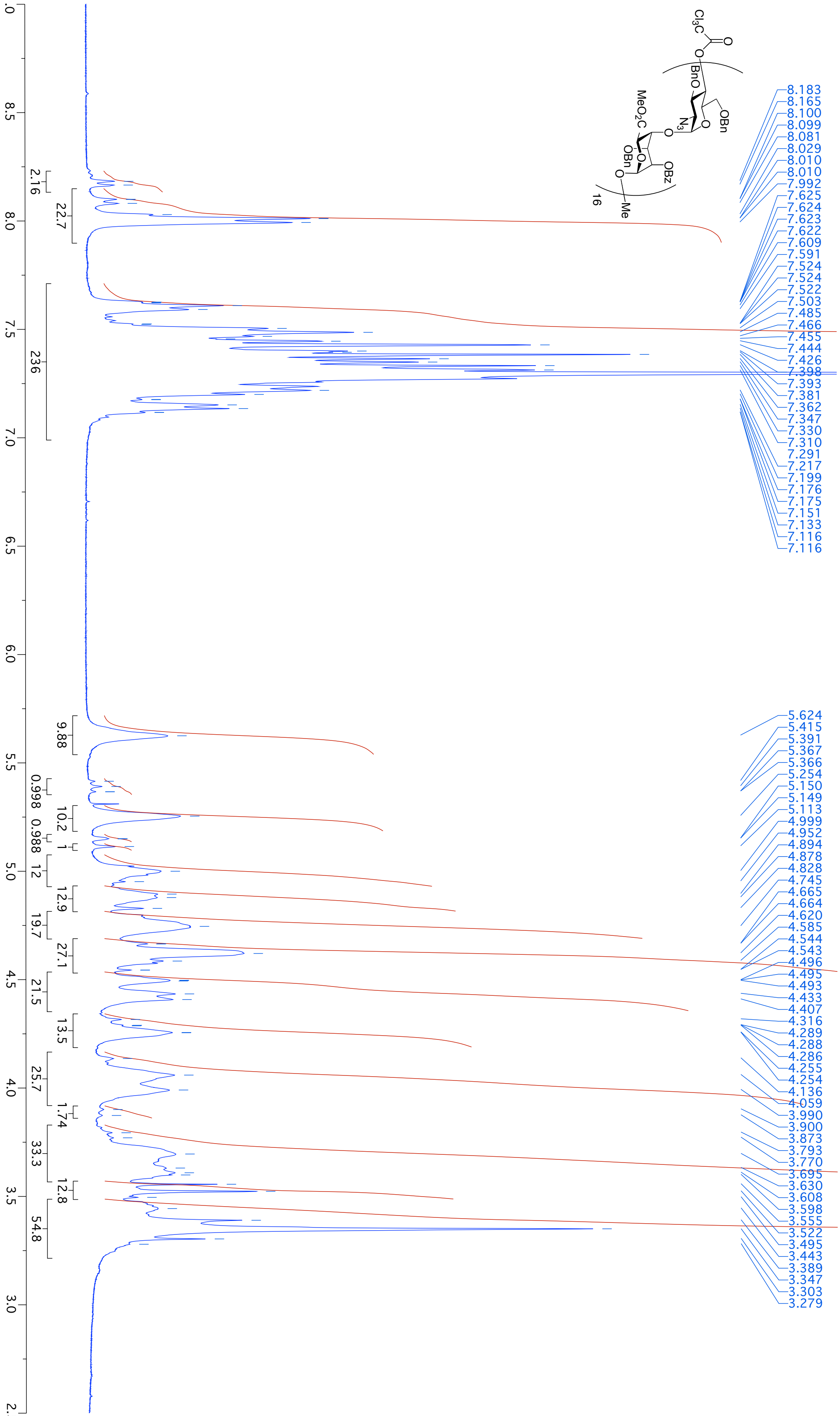

Supplementary Figure 48: COSY NMR (400 MHz; $CDCl_3$) spectrum for **12**

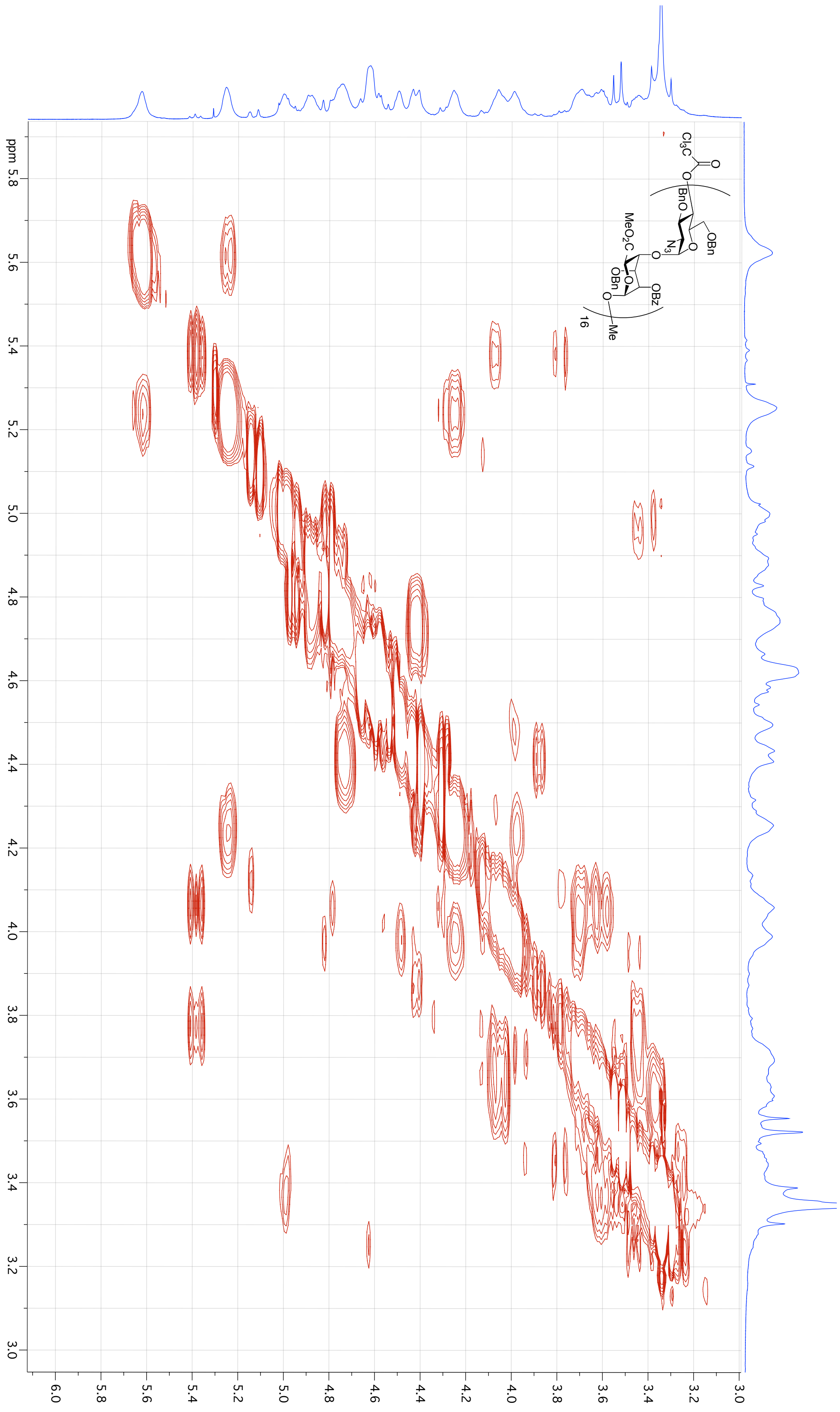

Supplementary Figure 49: HSQC NMR (400 MHz; $CDCl_3$) spectrum for **12**

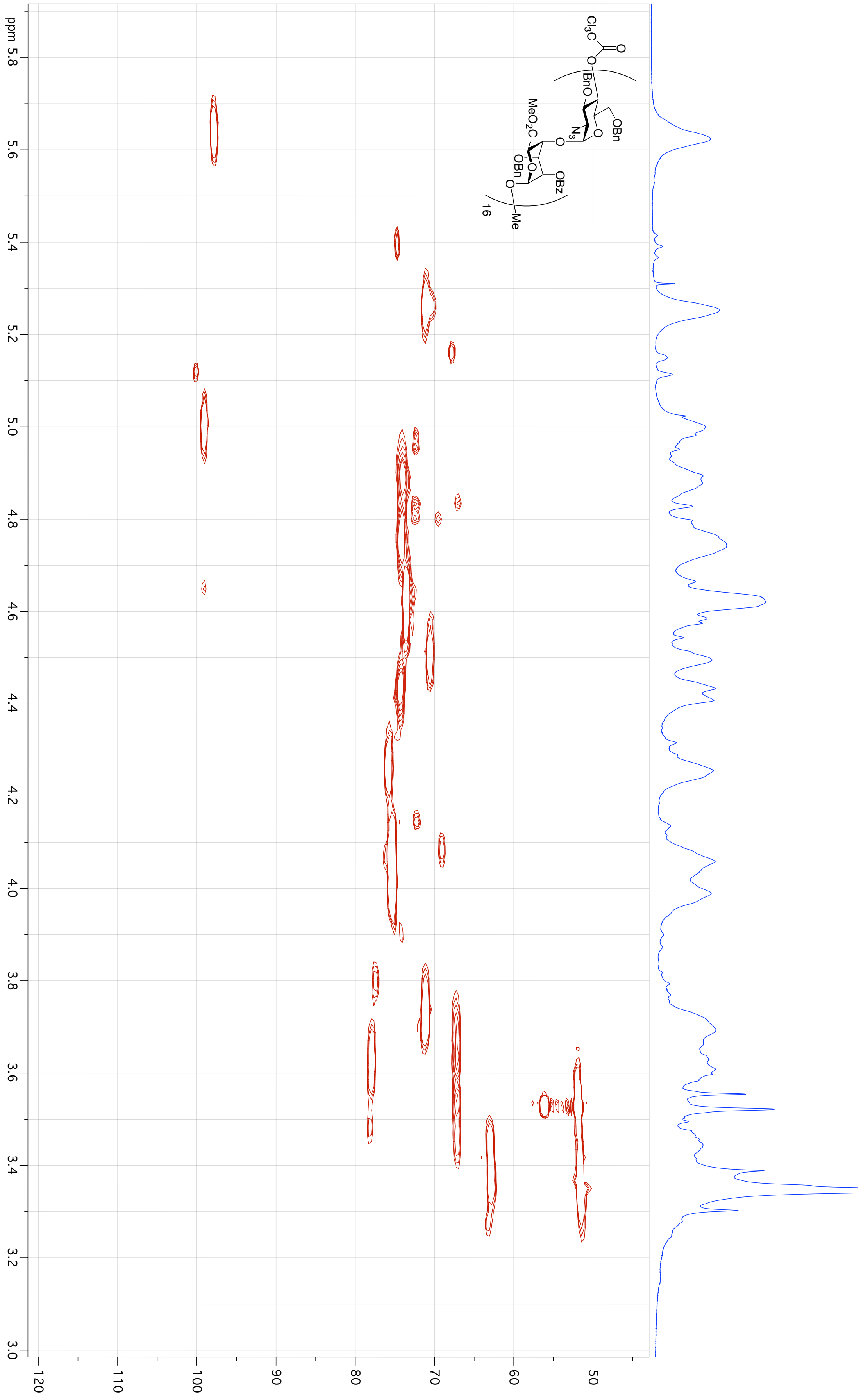

Supplementary Figure 50: MALDI MS spectrum for **12**

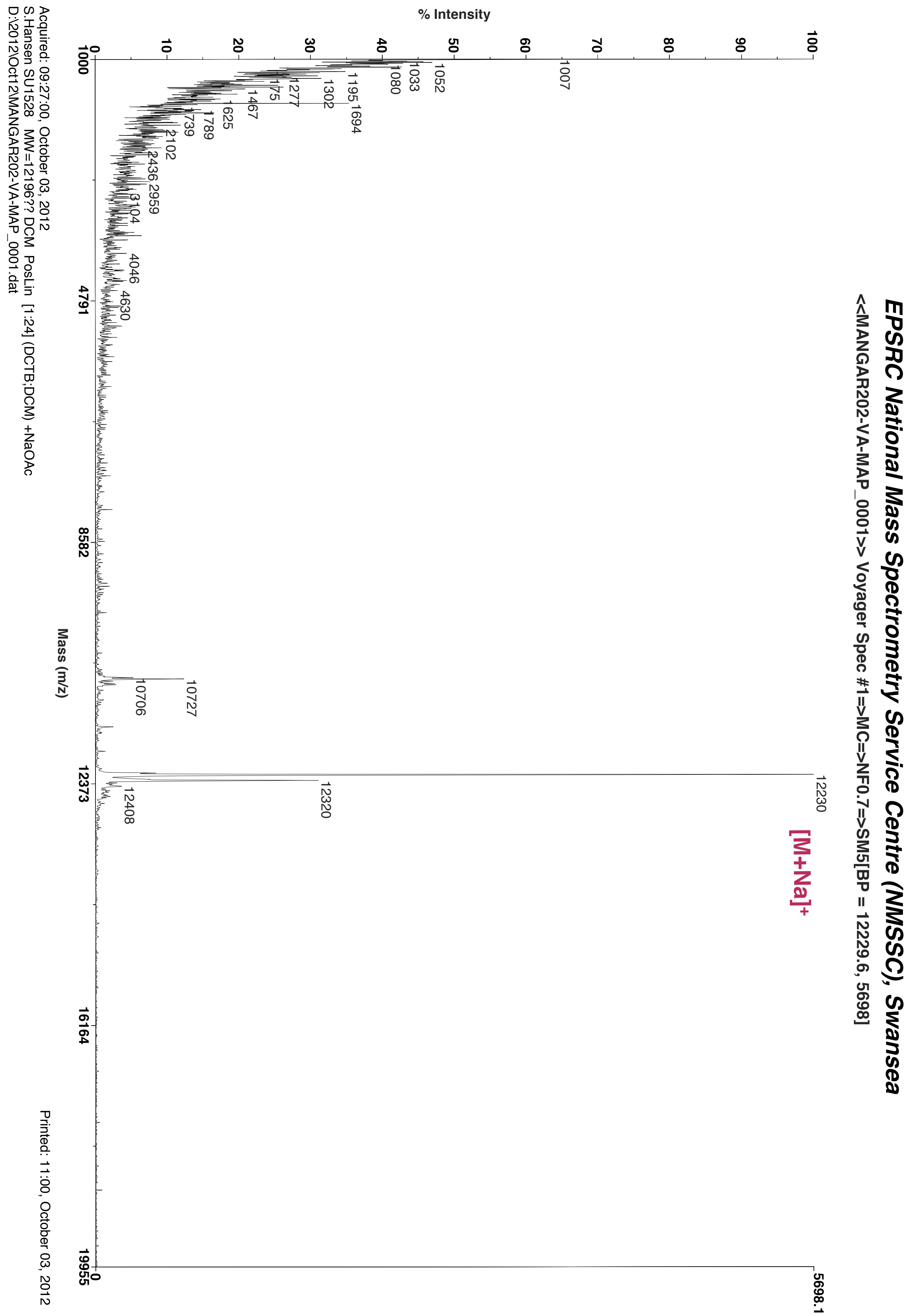

Supplementary Figure 51: ^{1}H NMR (400 MHz; $CDCl_3$) spectrum for **12**

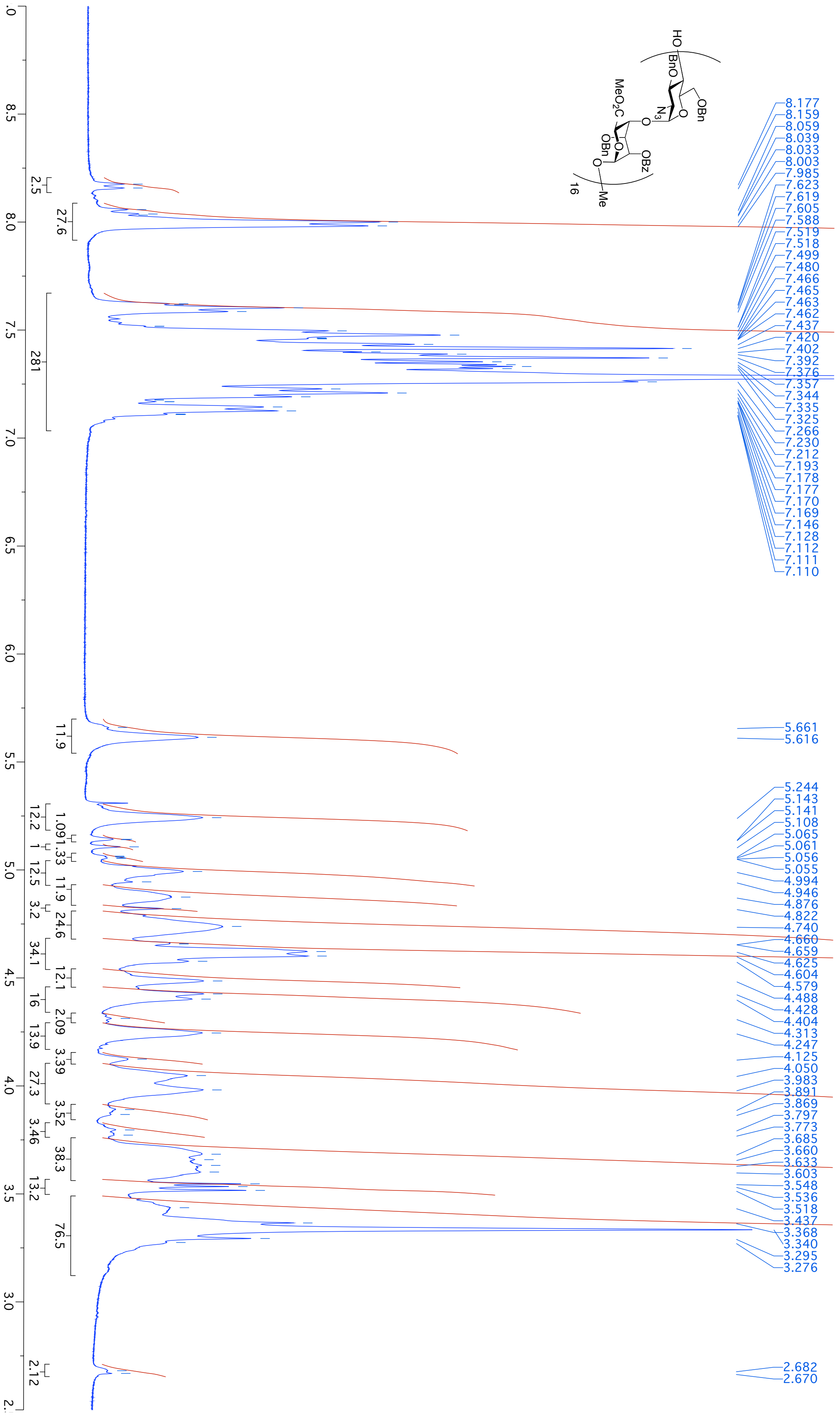

Supplementary Figure 52: COSY NMR (400 MHz; $CDCl_3$) spectrum for **13**

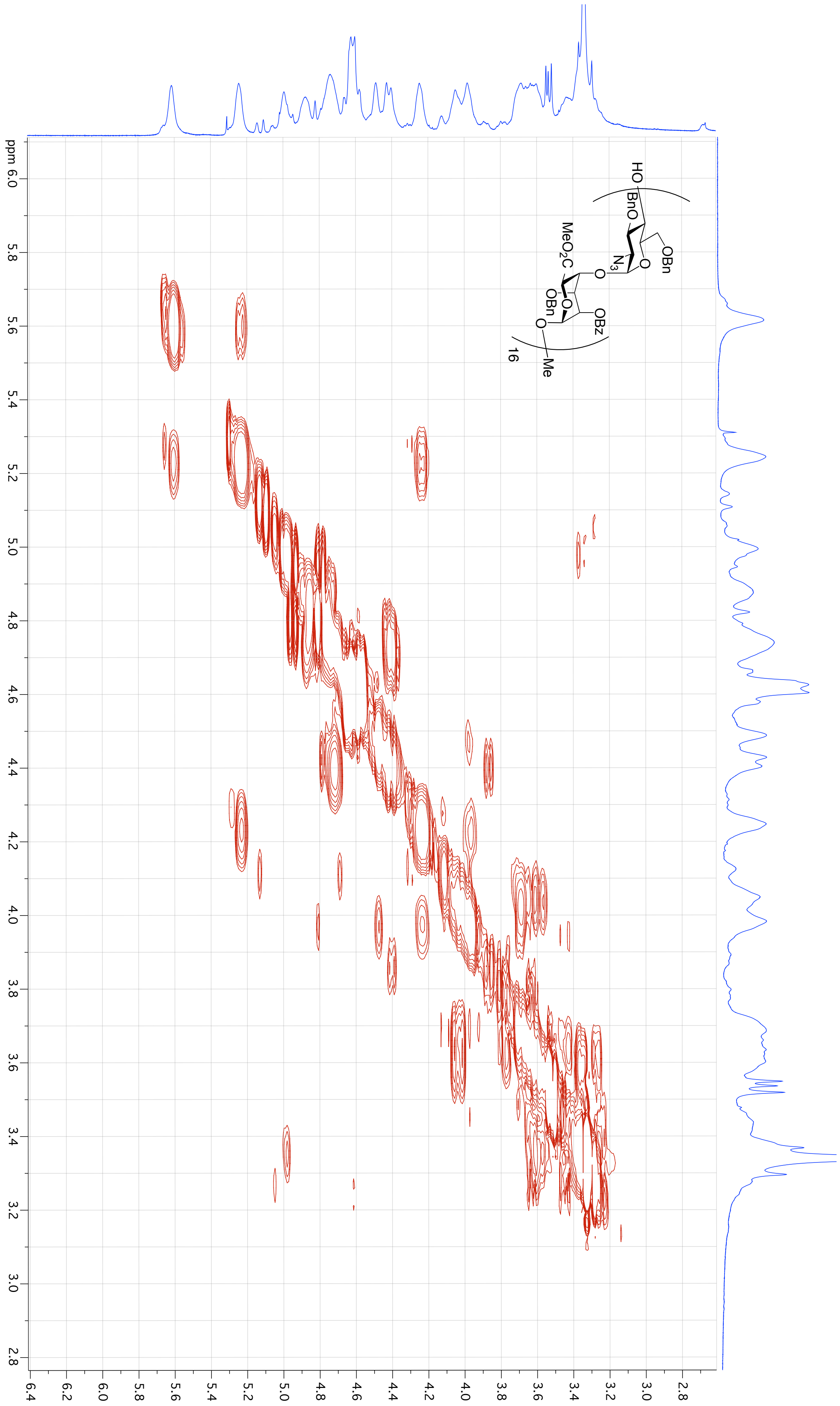

Supplementary Figure 53: MALDI MS spectrum for **13**

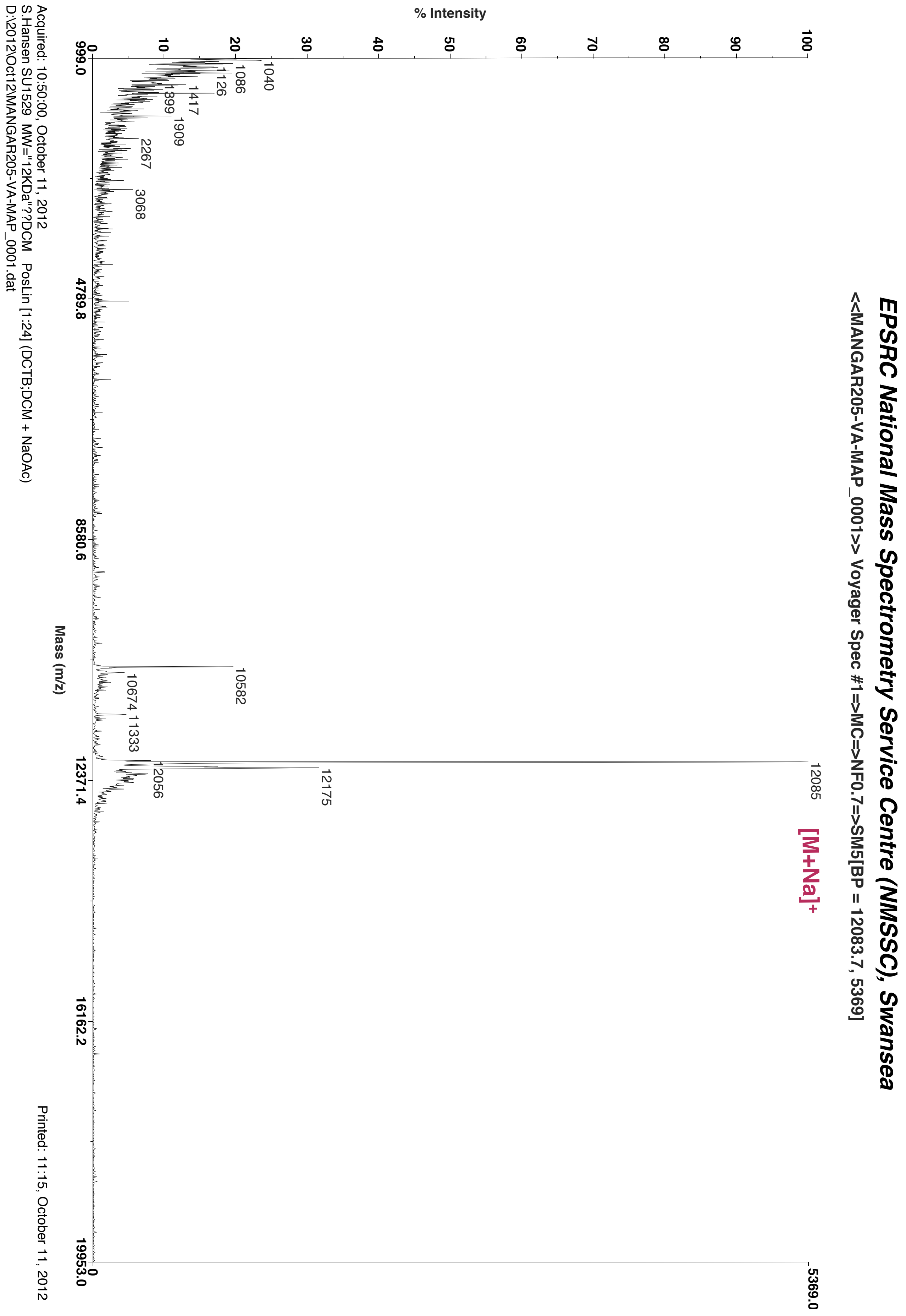

Supplementary Figure 54: ^{1}H NMR (800 MHz; $CDCl_3$) spectrum for **14**

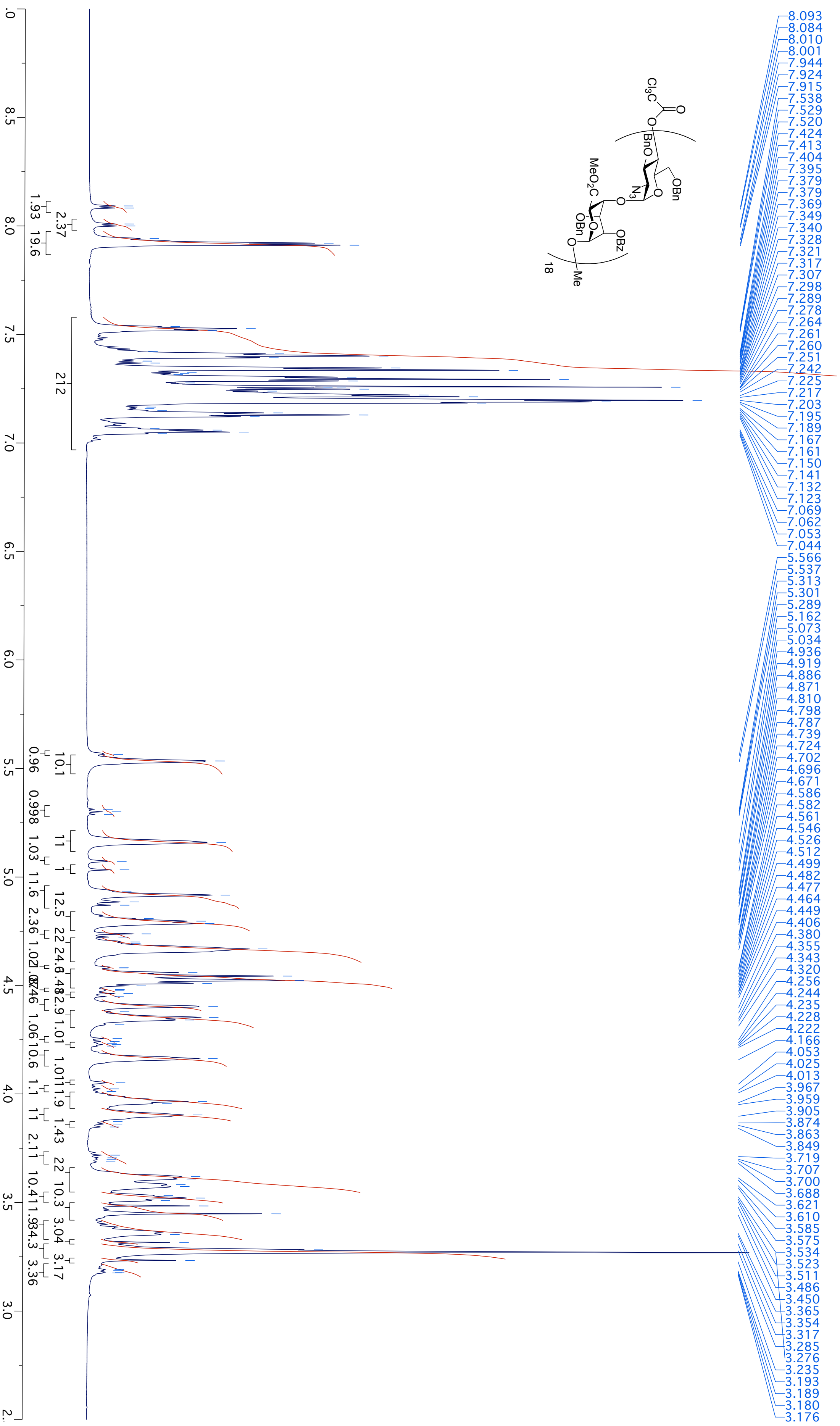

Supplementary Figure 55: HSQC NMR (800 MHz; $CDCl_3$) spectrum for **14**

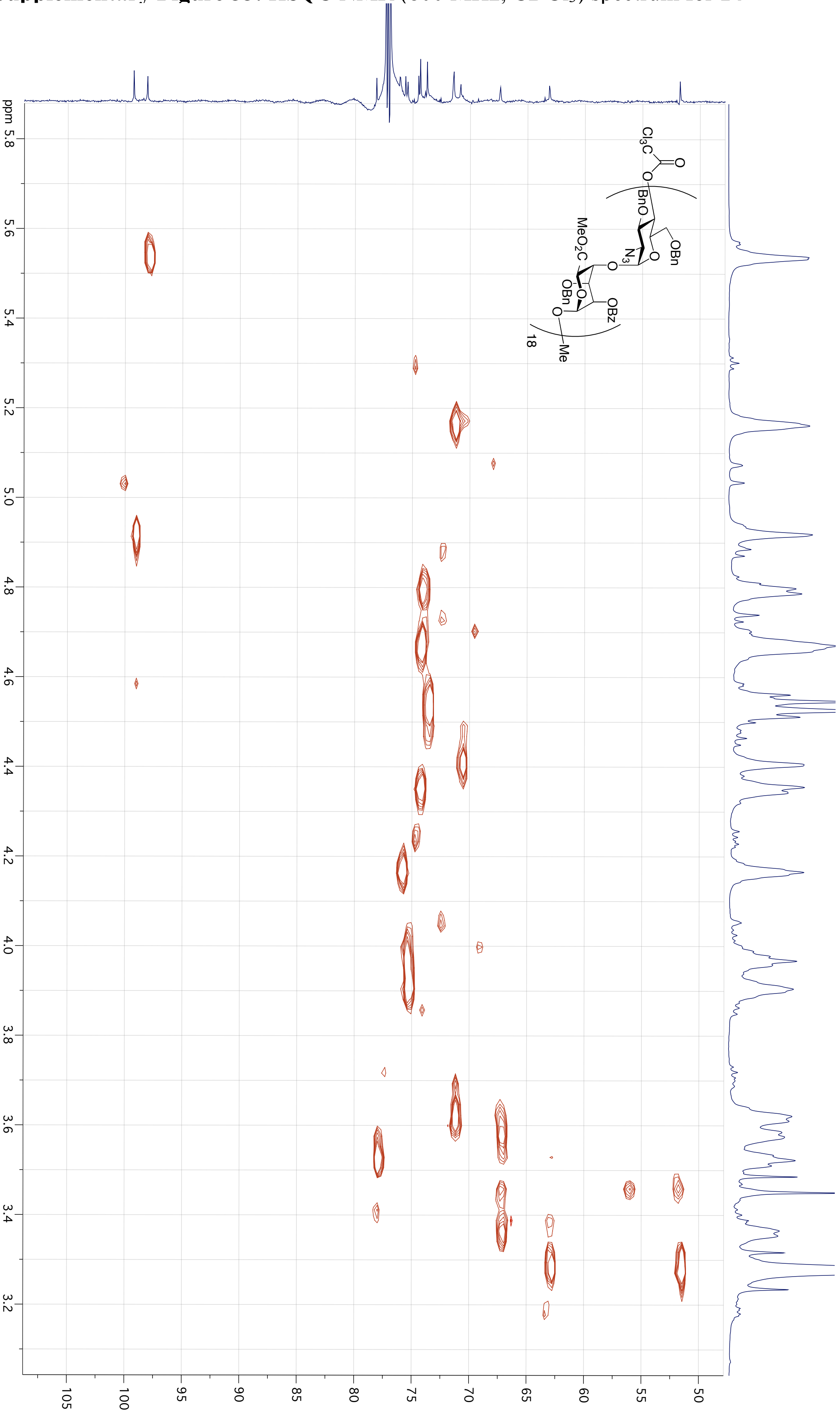

Supplementary Figure 56: NUS Pure Shift HSQC NMR (800 MHz; $CDCl_3$) spectrum for **14**

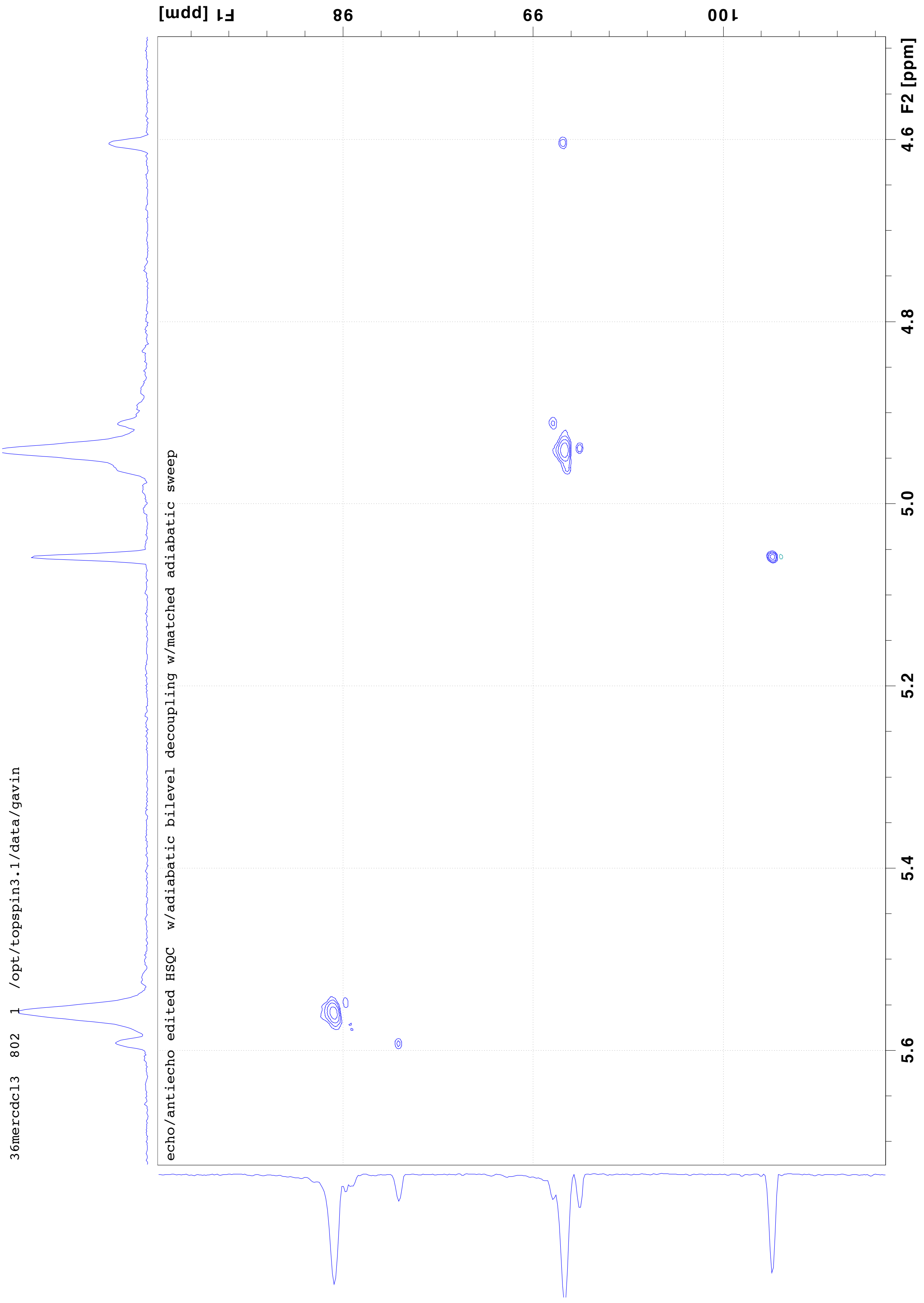

Supplementary Figure 57: (a) NUS Pure Shift HSQC NMR (800 MHz; $CDCl_3$) spectrum for **14 (red) overlayed with 12mer** 2 **(blue) - anomeric centres region**. (b) sum of the crosspeak intensities of the 3 peaks at 4.94 H 99.15 C, relative to that at 4.91 H 99.1 C, as a function of oligomer length.

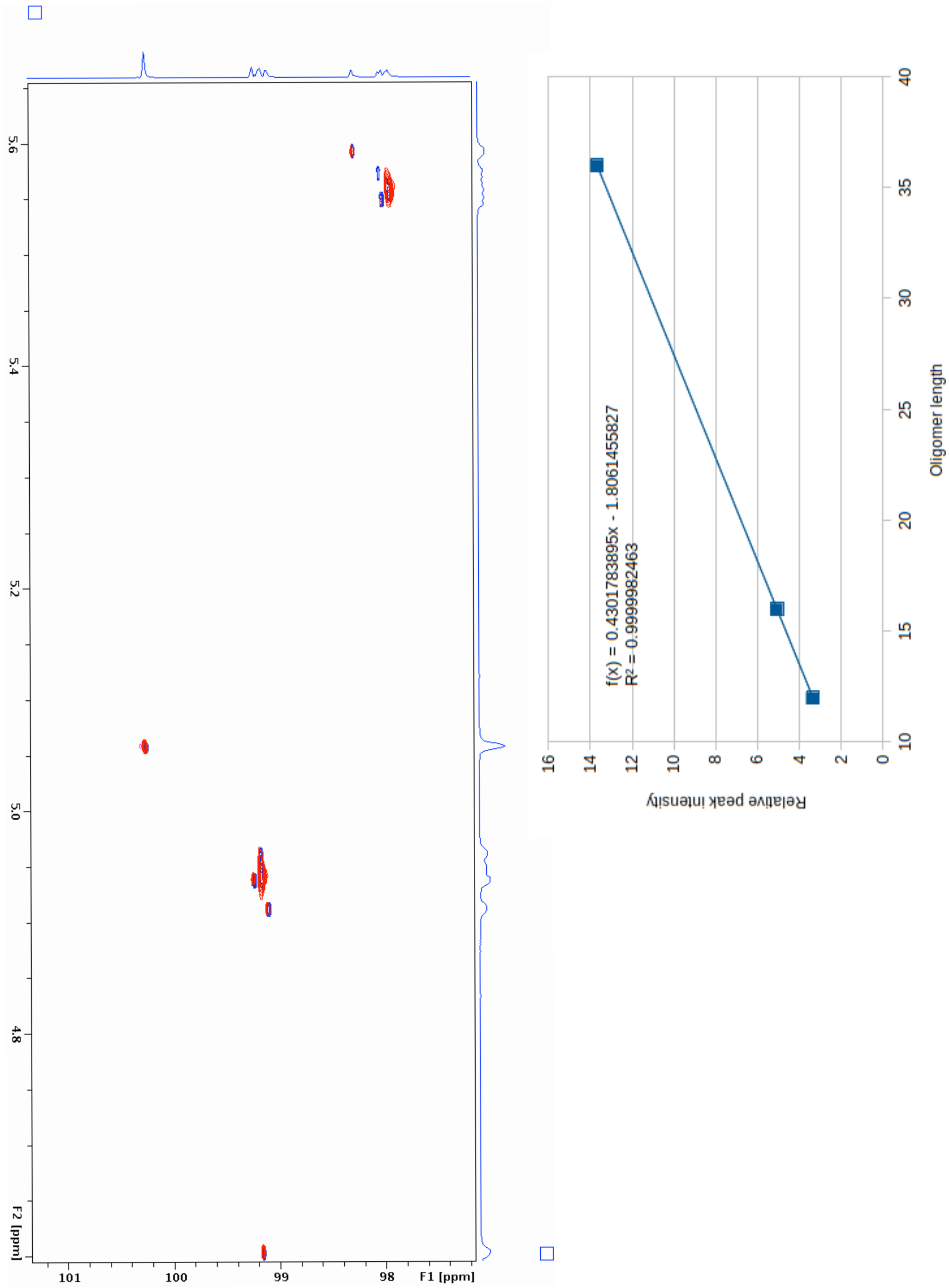

Supplementary Figure 58: MALDI MS spectrum for **14**

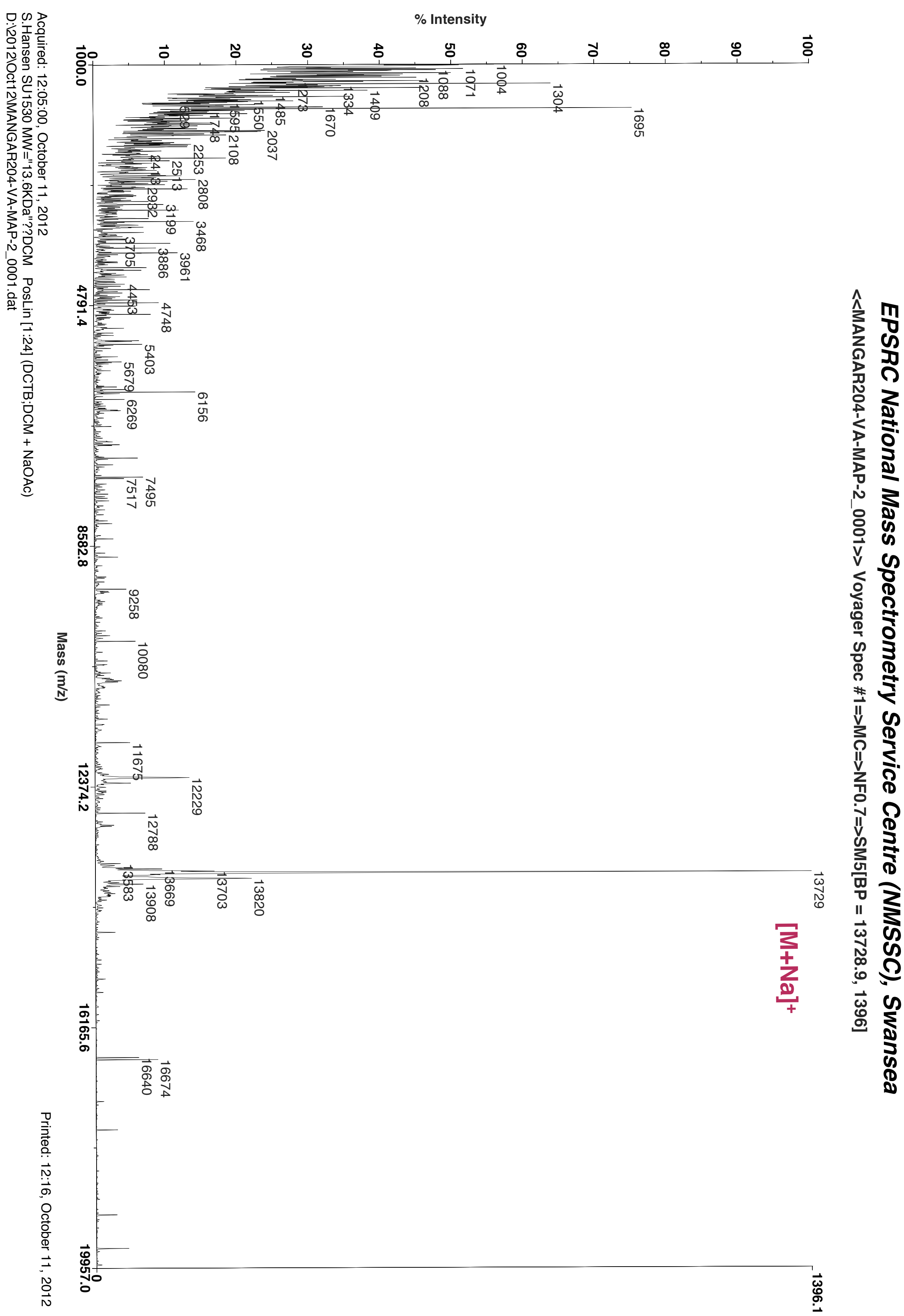

Supplementary Figure 59: ^{1}H NMR (800 MHz; $CDCl_3$) spectrum for **16**

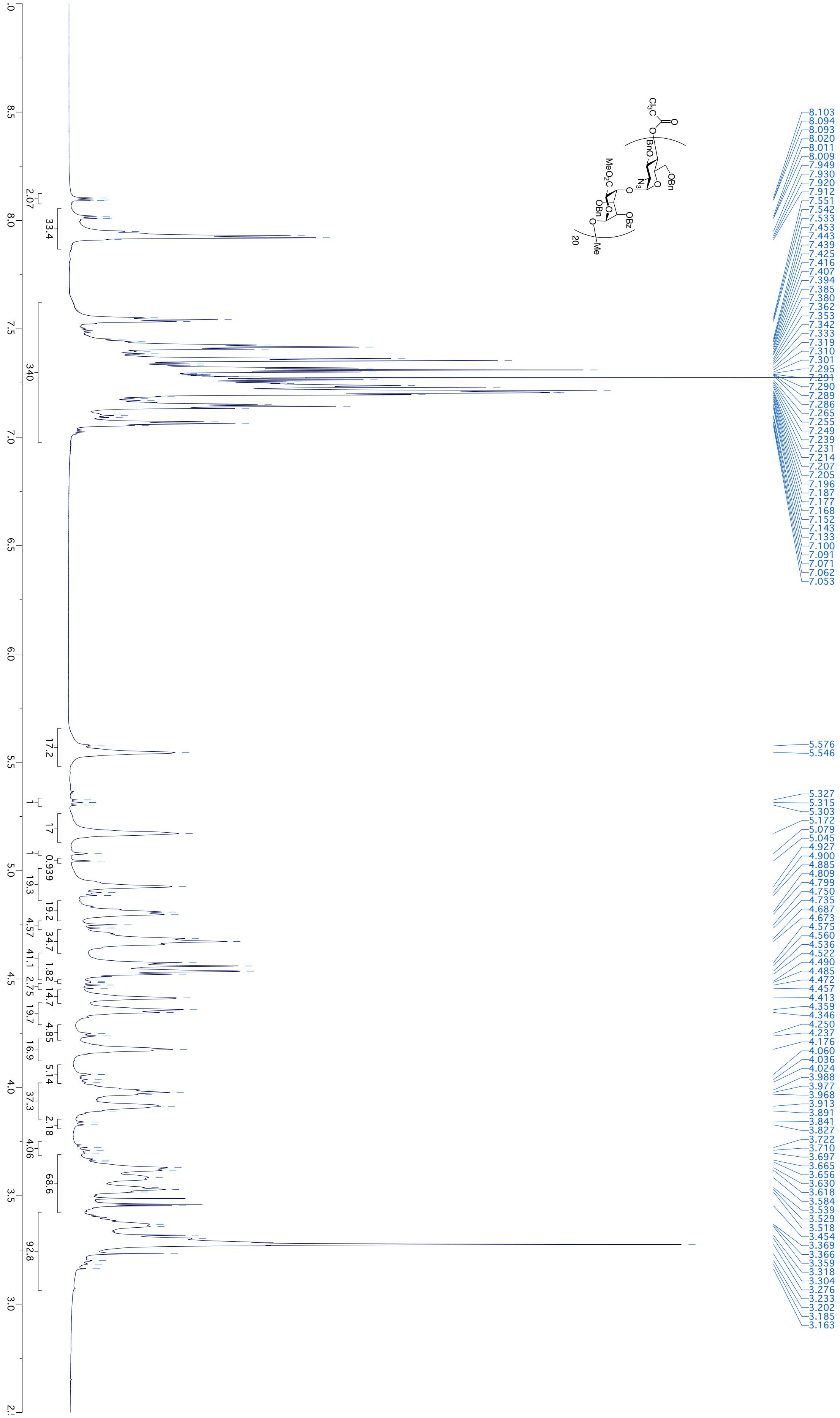

Supplementary Figure 60: COSY NMR spectrum for **16**

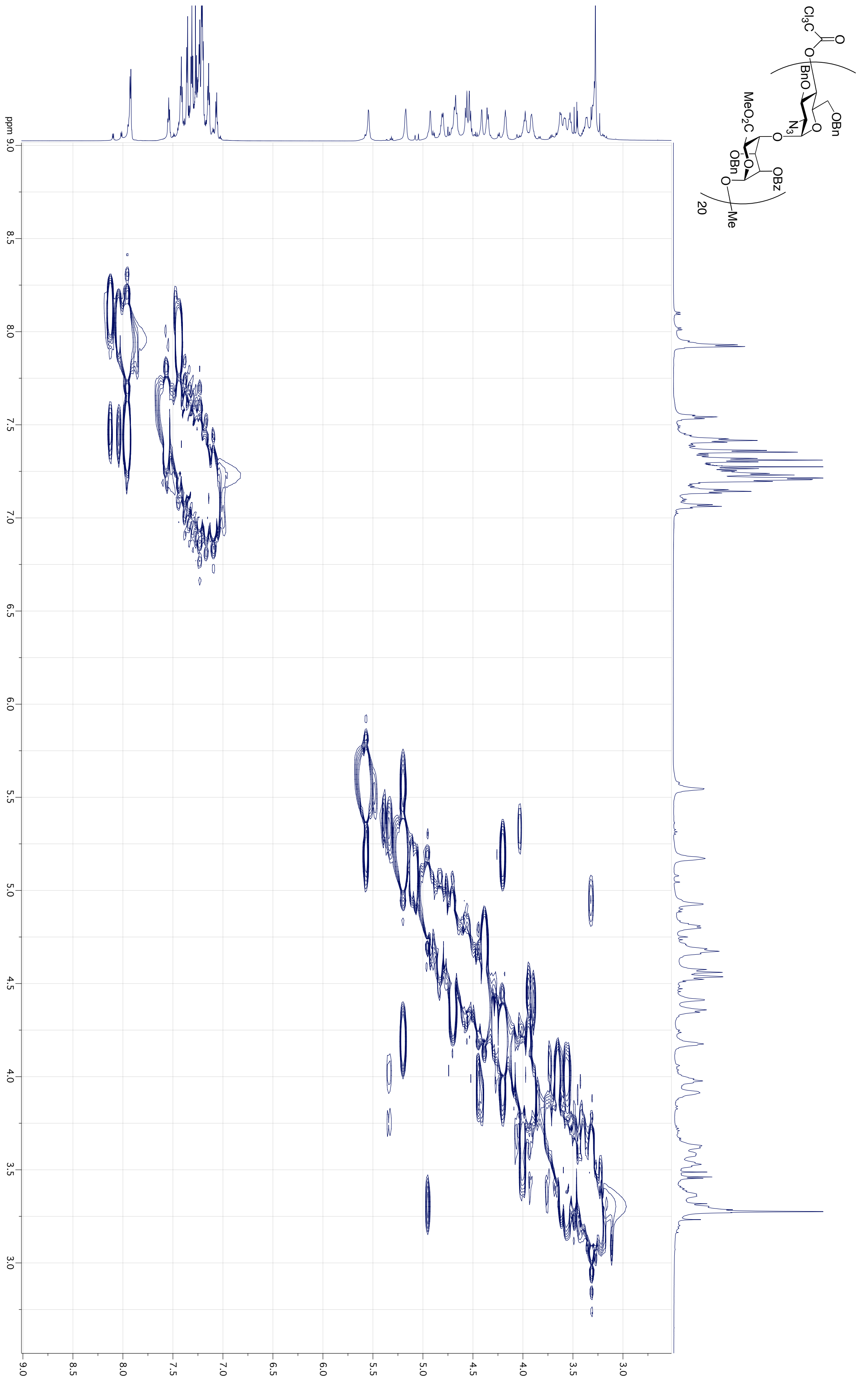

Supplementary Figure 61: HSQC NMR spectrum for **16**

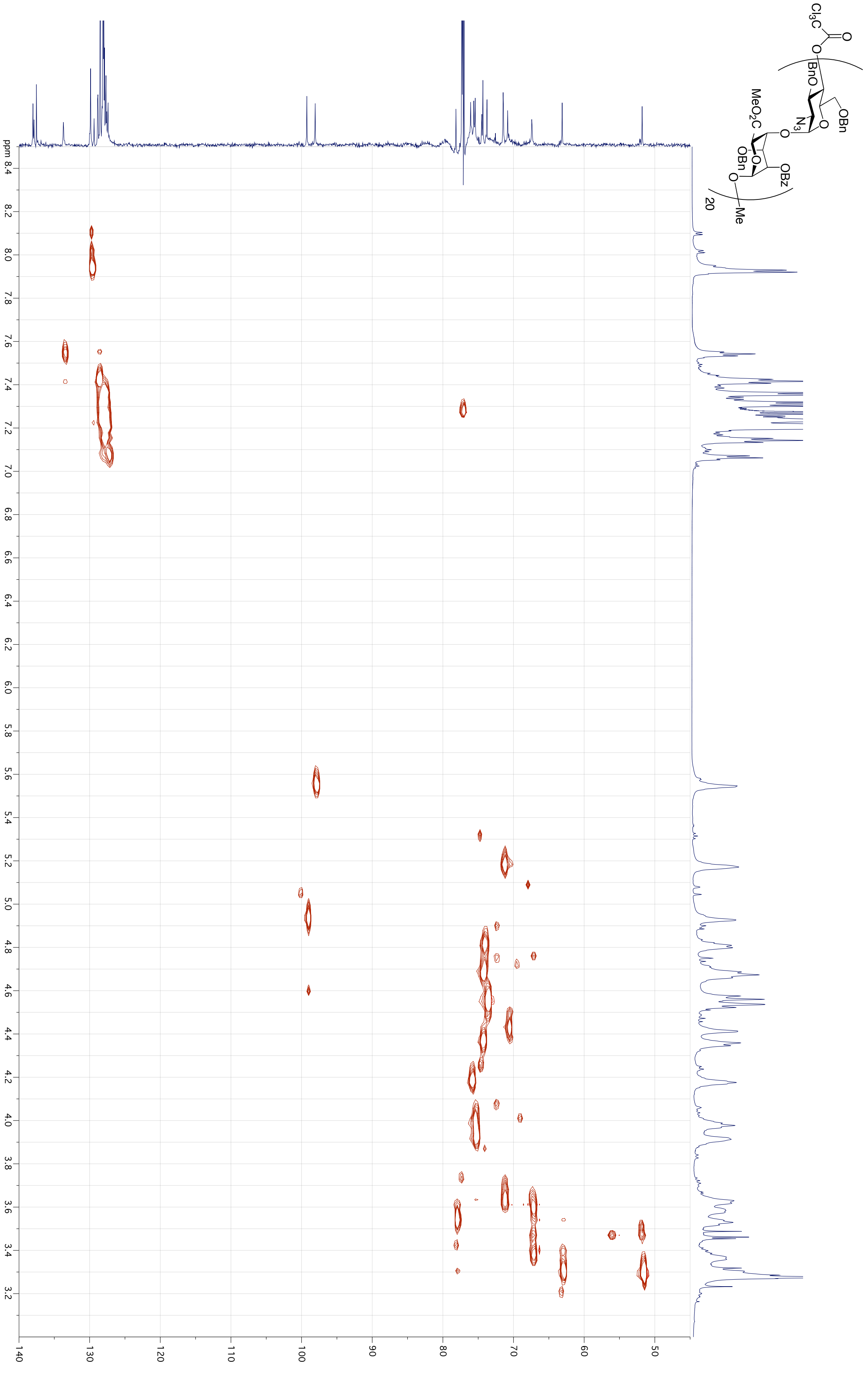

Supplementary Figure 62: ^{13}C NMR (201 MHz; $CDCl_3$) spectrum for **16**

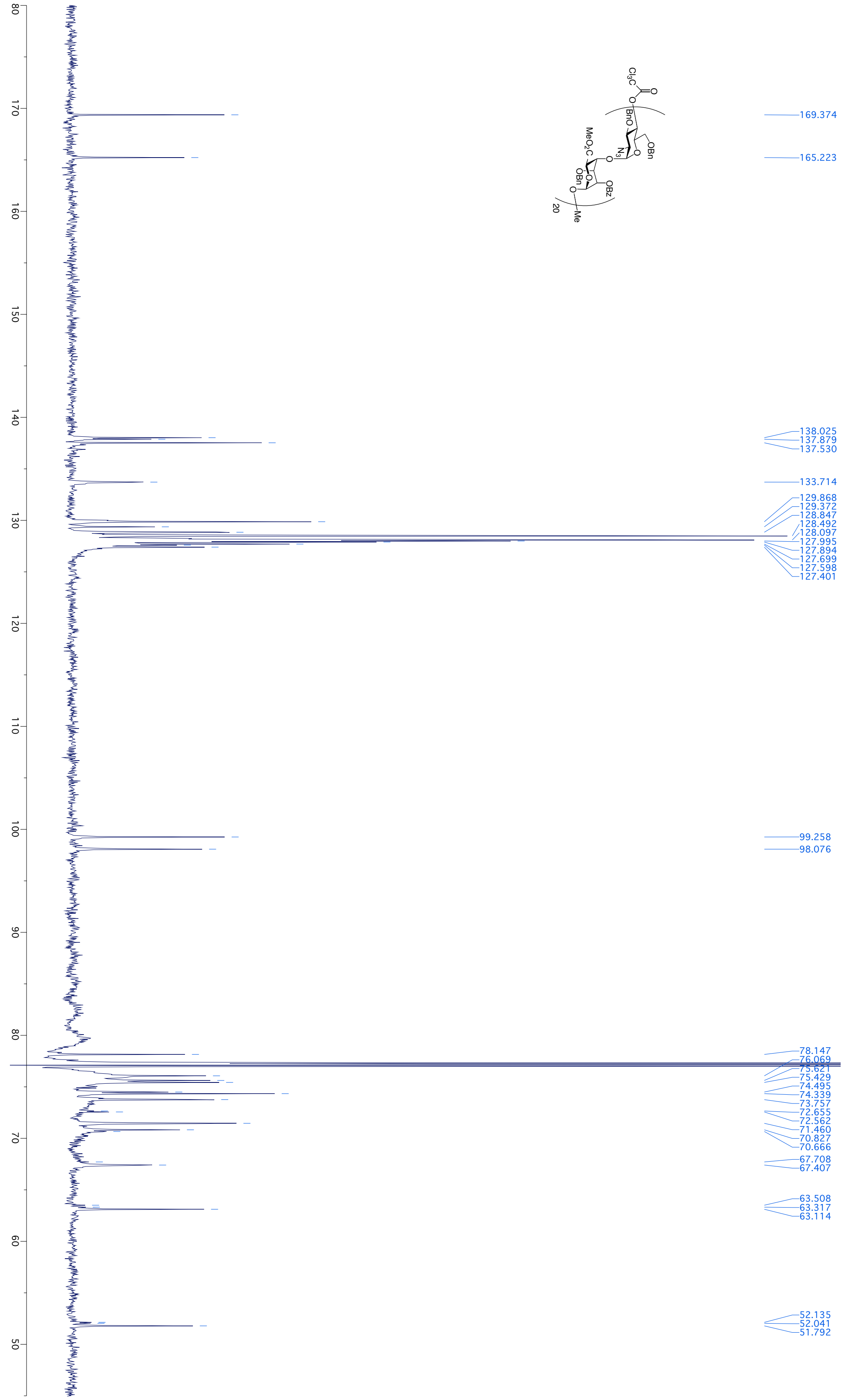

Supplementary Figure 63: MALDI MS spectrum for **16**

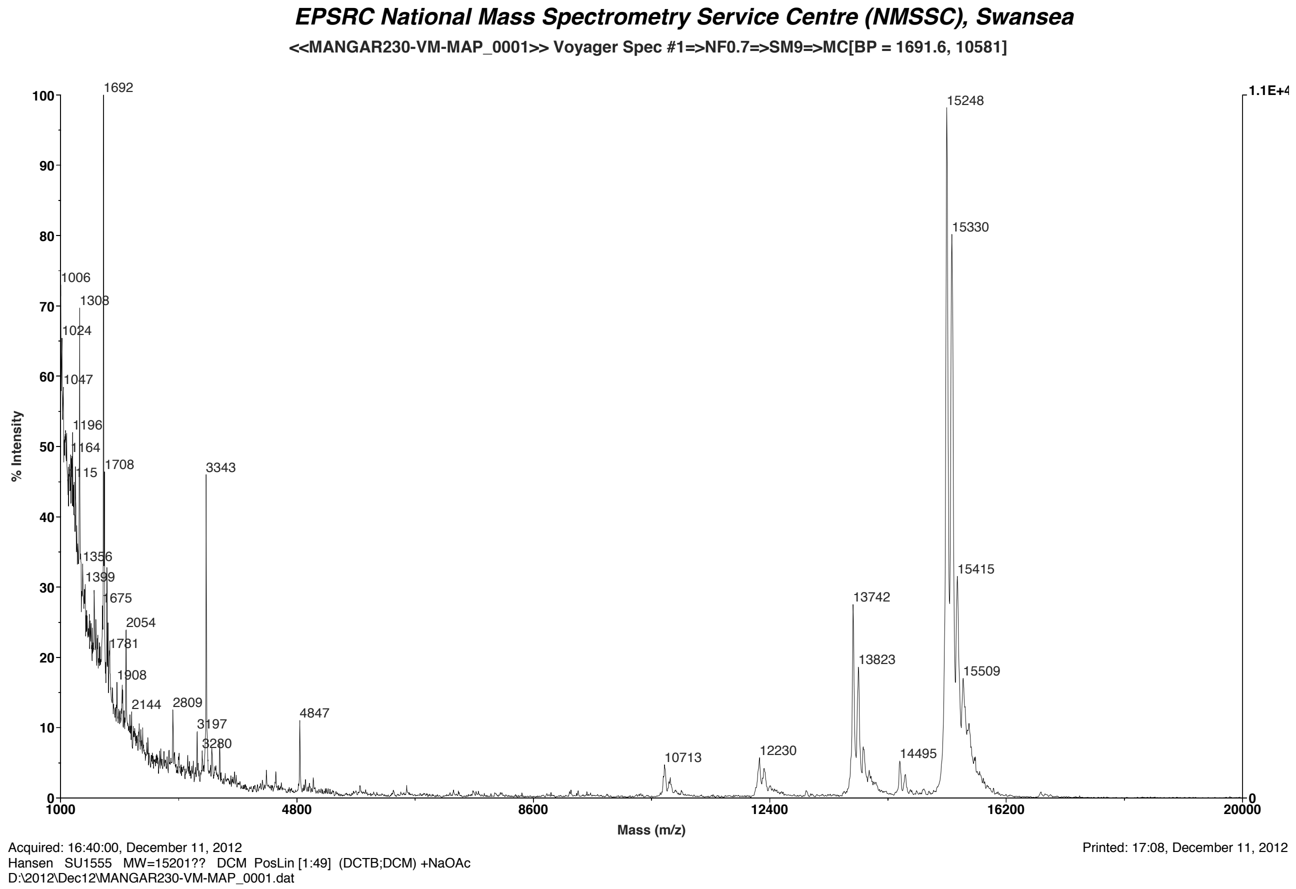

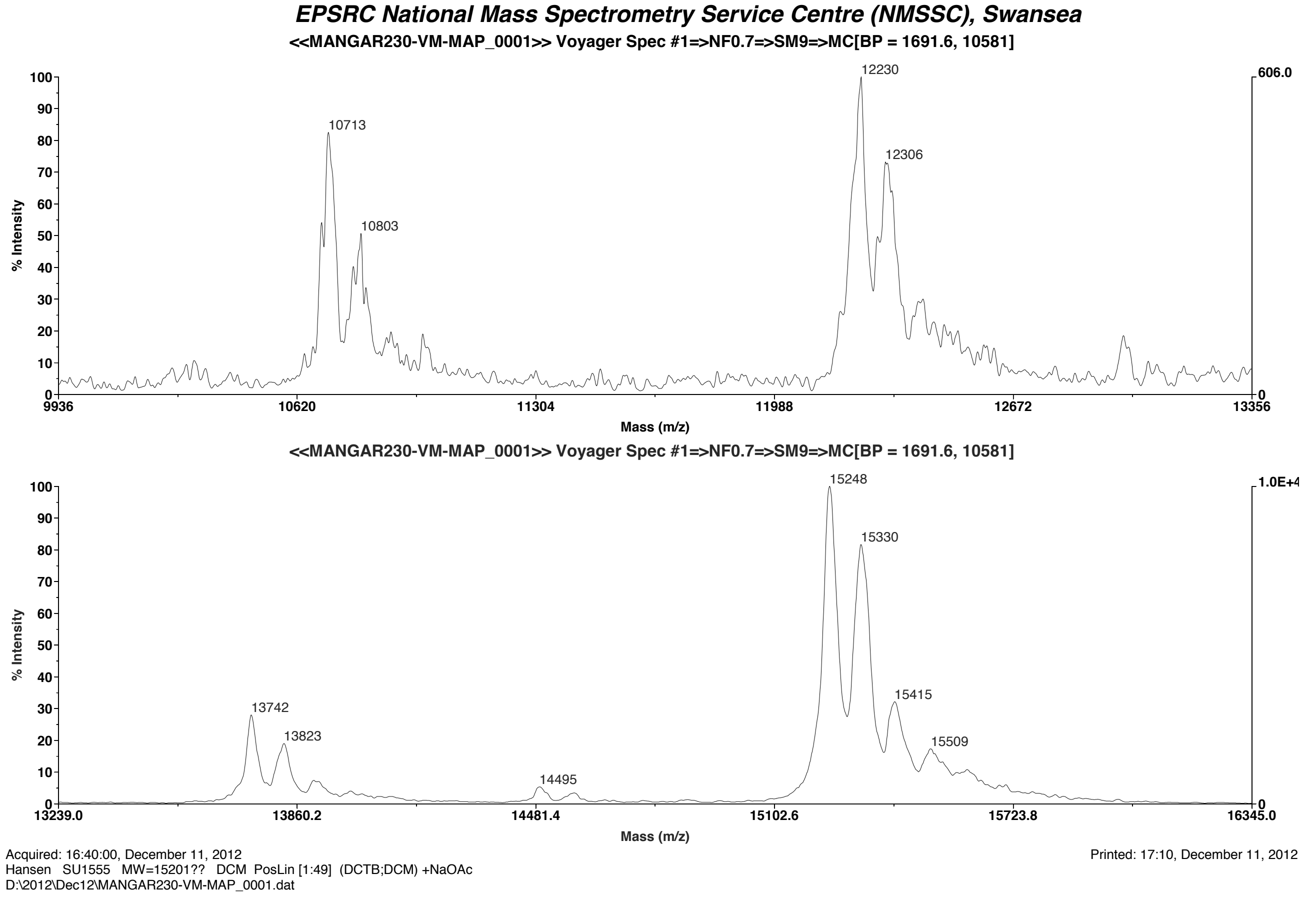

Supplementary Figure 64: ^{1}H NMR (800 MHz; D_2O) spectrum for **18**

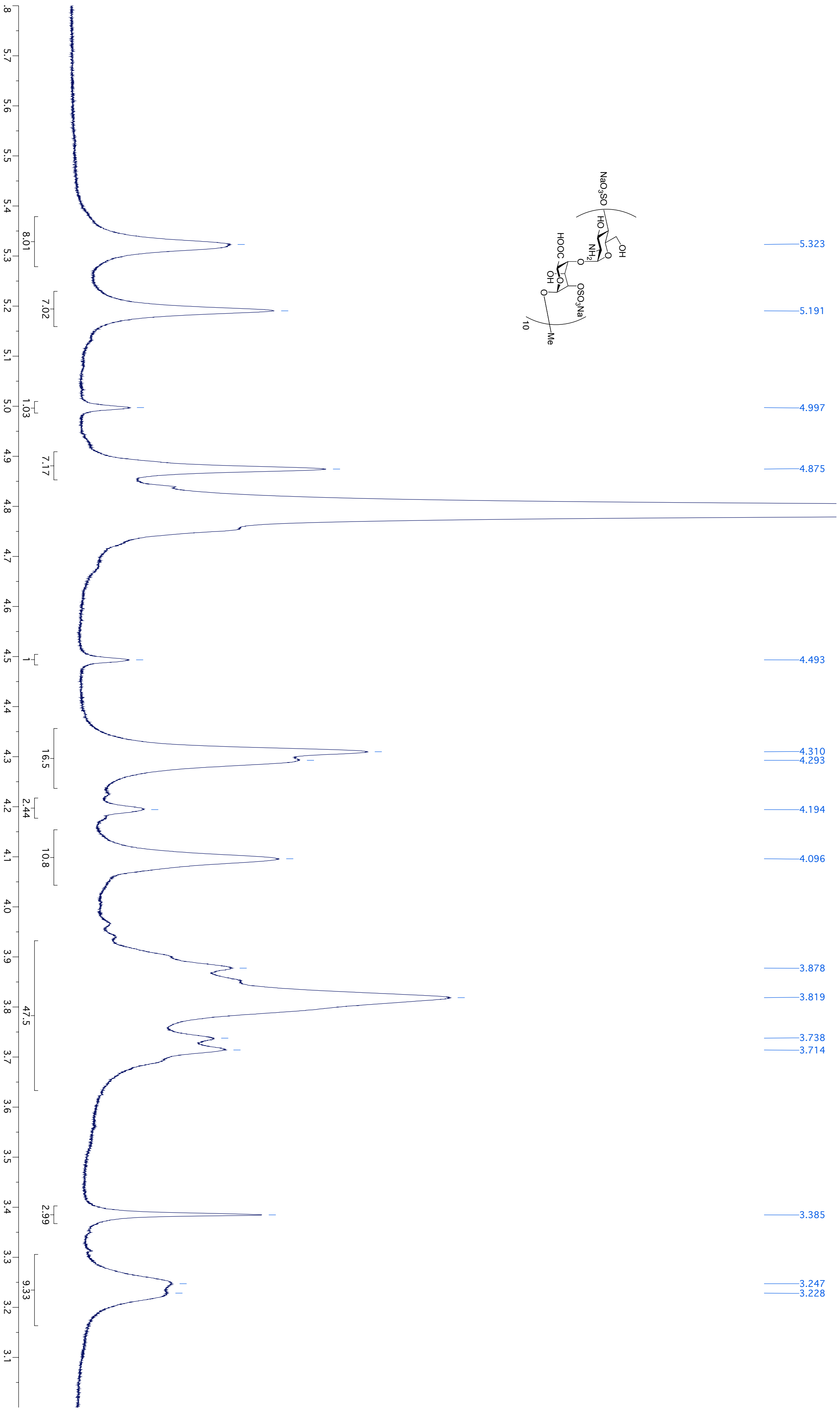

Supplementary Figure 65: ^{1}H water suppression NMR (800 MHz; D_2O) spectrum for **18**

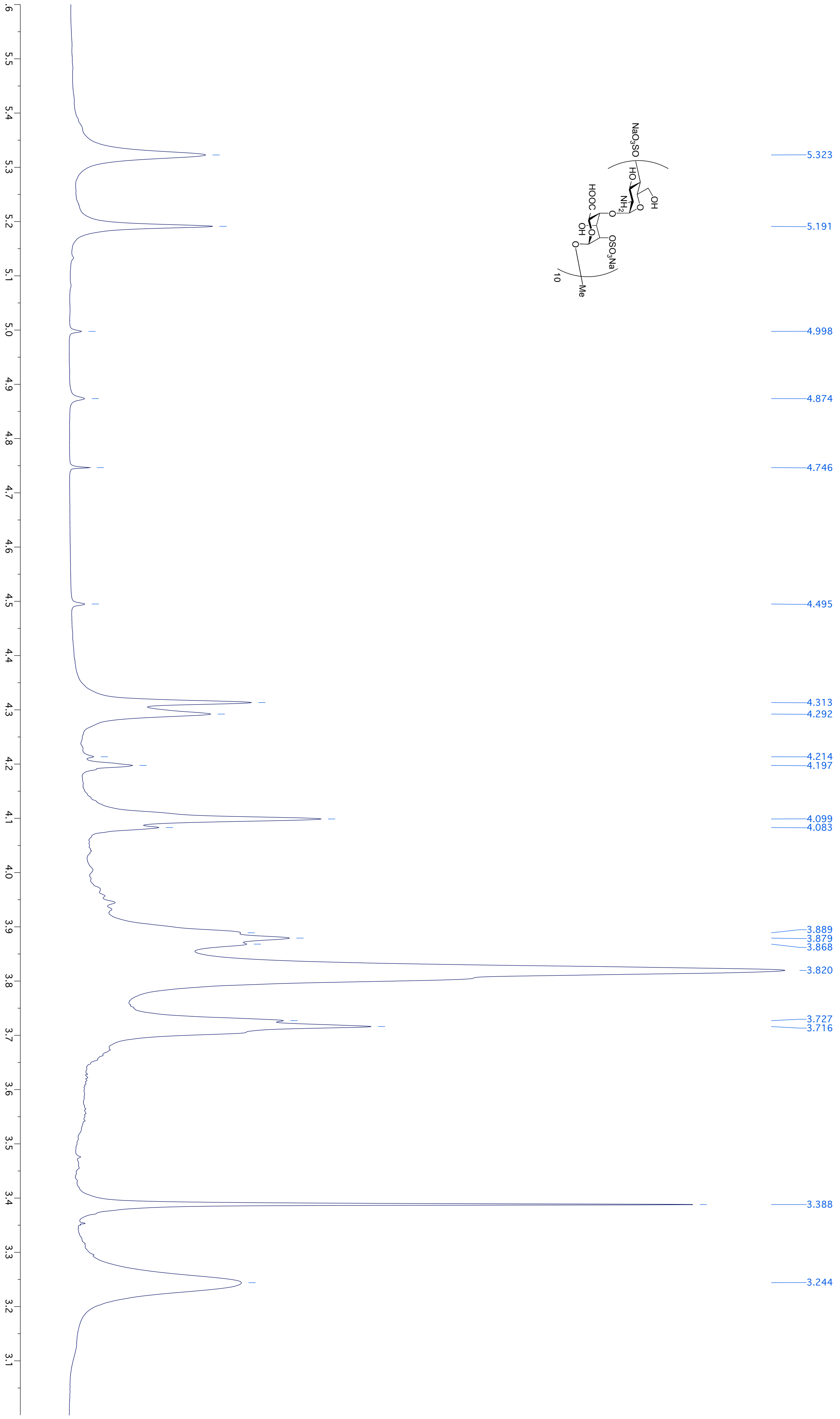

Supplementary Figure 66: HSQC NMR (800 MHz; D_2O) spectrum for **18**

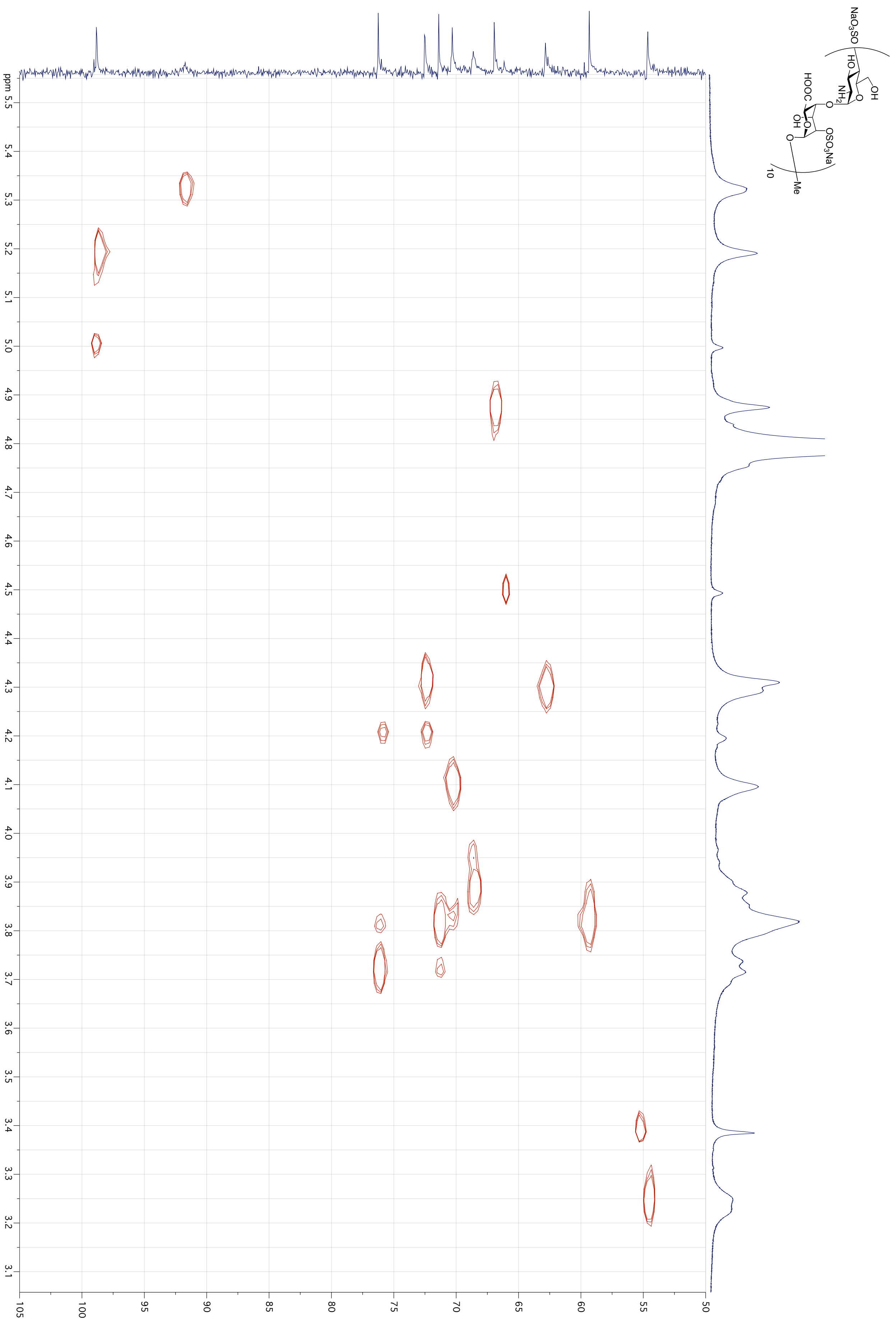

Supplementary Figure 67: FT NSI MS spectrum for **18**

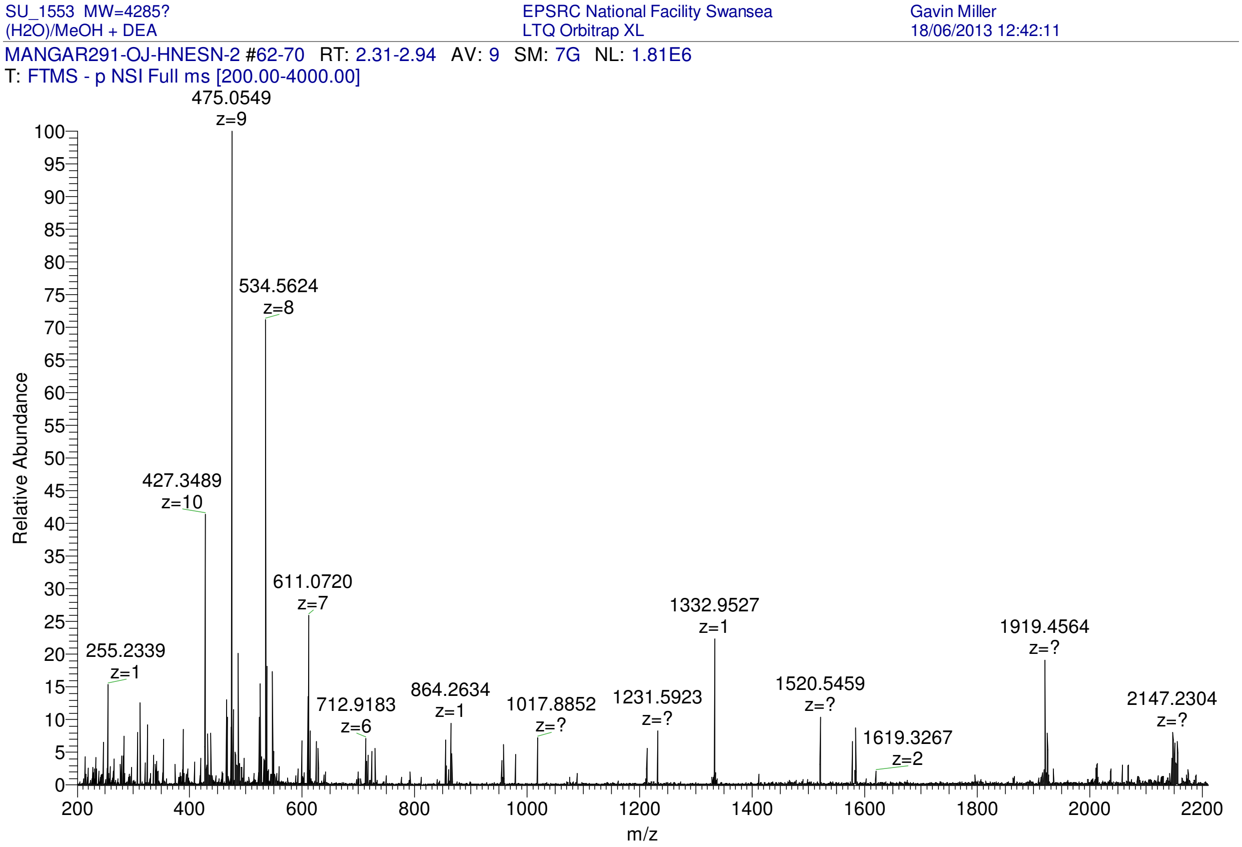

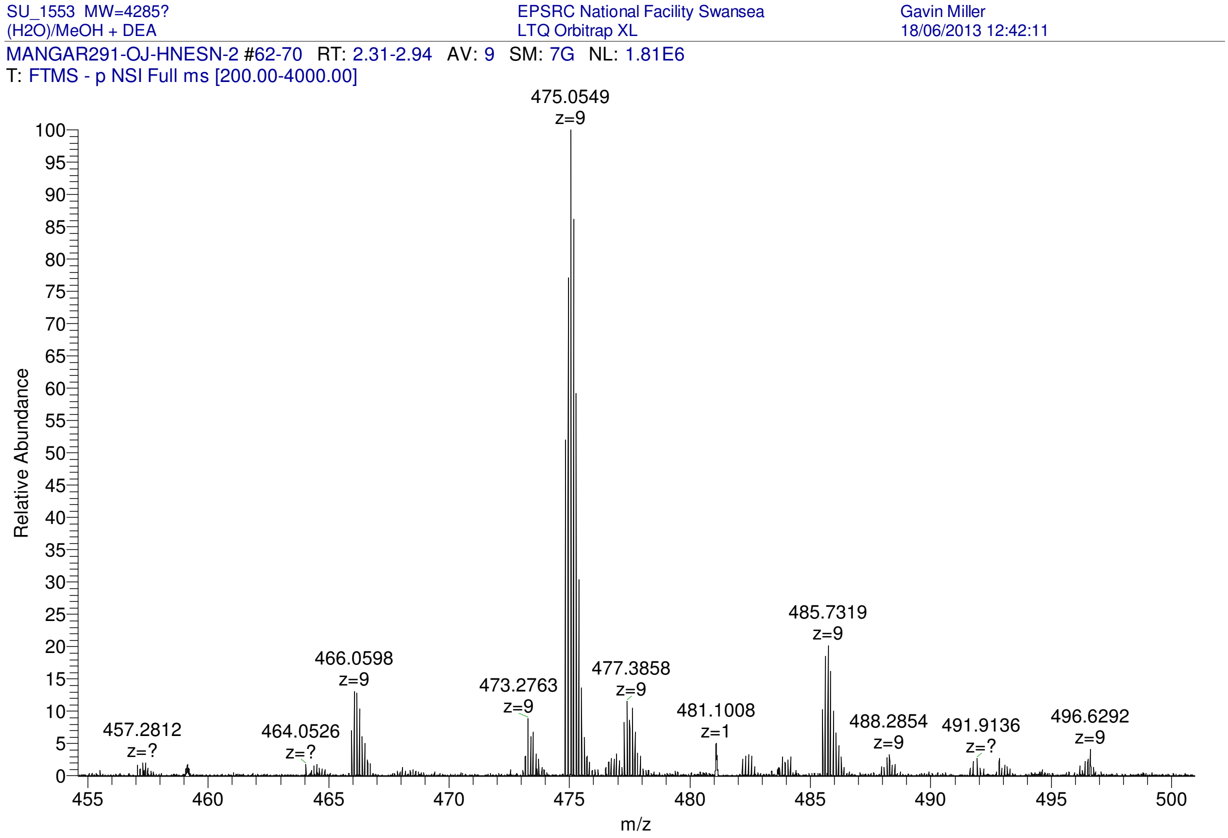

Supplementary Figure 68: Isotope Pattern for **18**

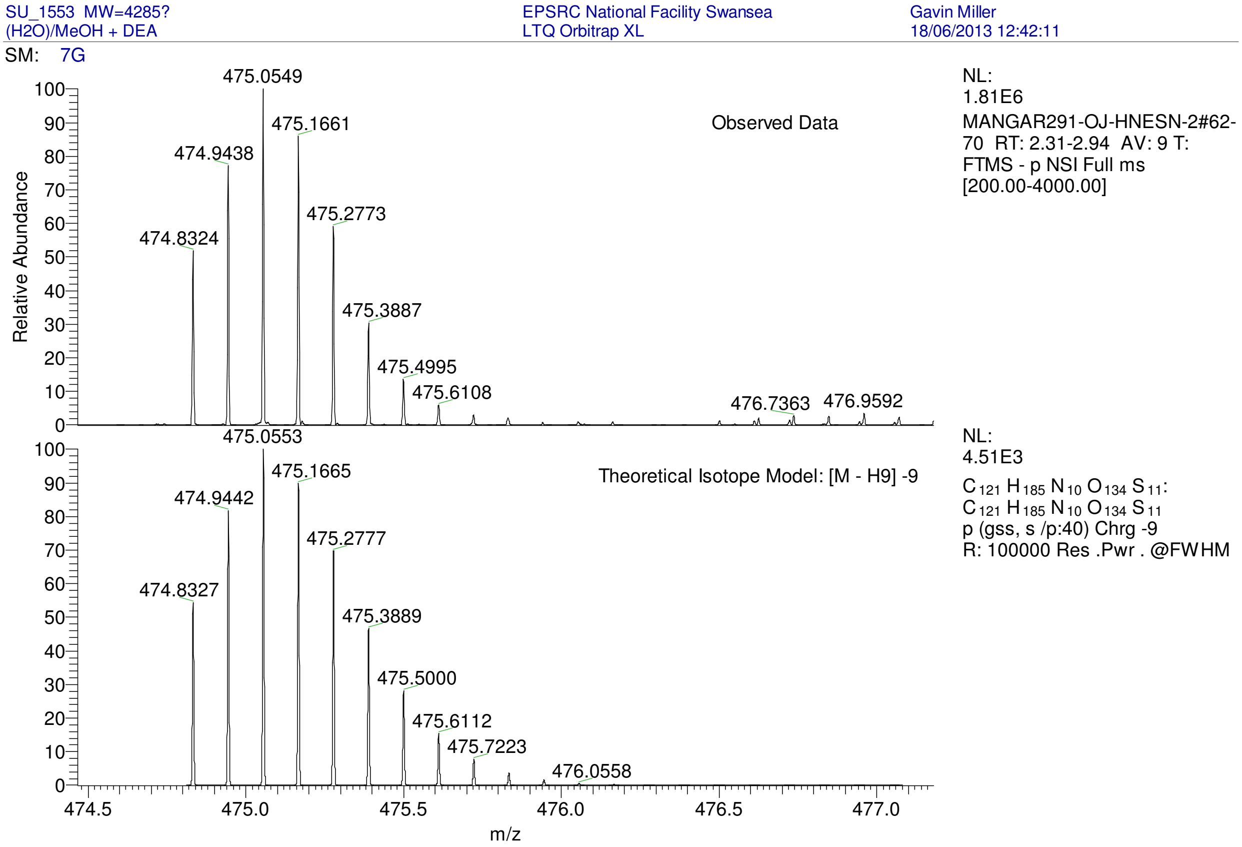

Supplementary Figure 69: ^{1}H NMR (800 MHz; D_2O) spectrum for **19**

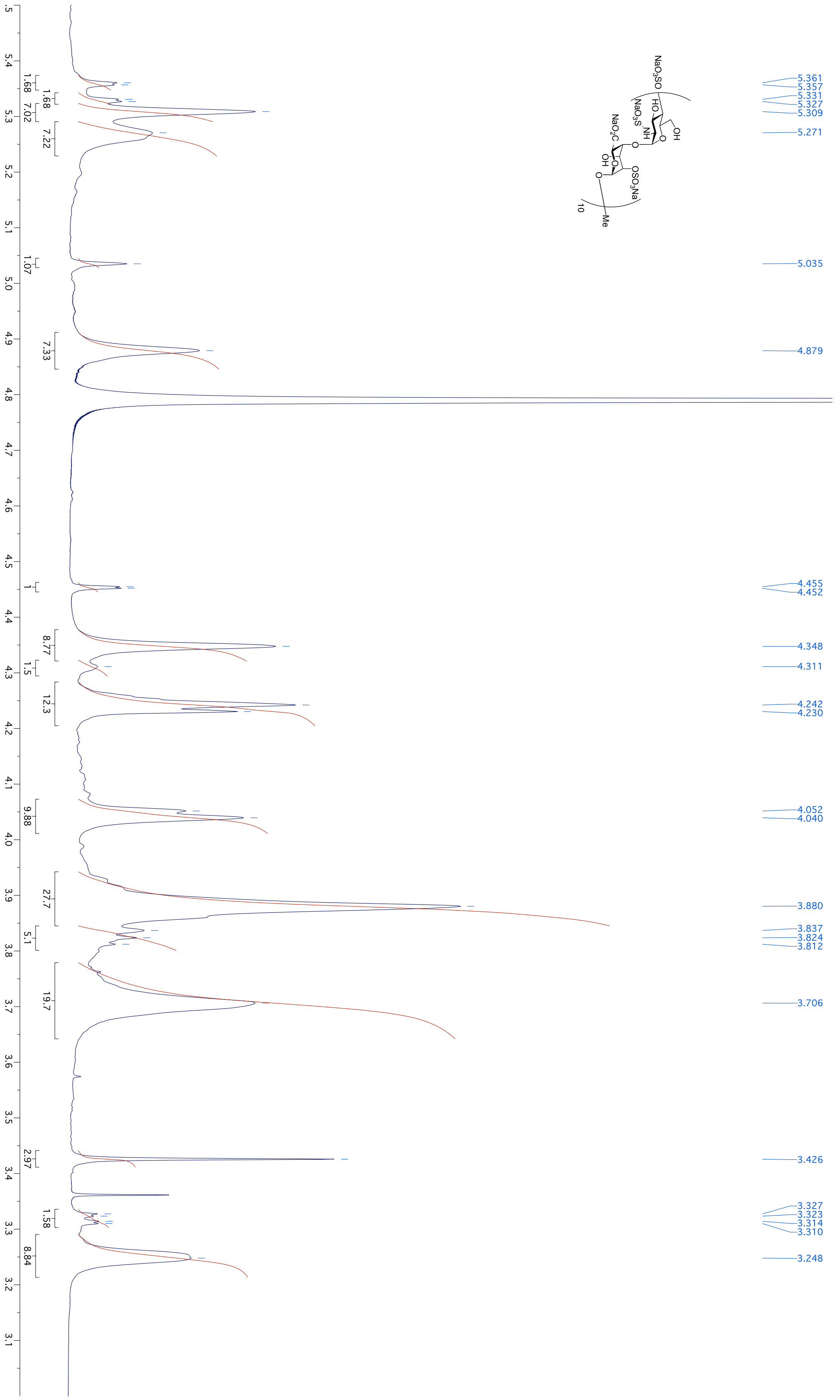

Supplementary Figure 70: NMR analysis of GlcN *N*-sulfation of icosasaccharide (A4I2-[A0I2]9)-OMe **18** to **19.**

COSY and HSQC correlation of icosasaccharide (S4I2-[S0I2]9)-OMe **19** confirms peak (blue arrow) is additional H-2 NS correlated to H-1. See overlay spectra for **18** (blue) and **19** (red), showing coincidence of terminal methoxy methyl resonances, and definitive shift of H-2 signals due to *N*-2-sulfation in **19**.

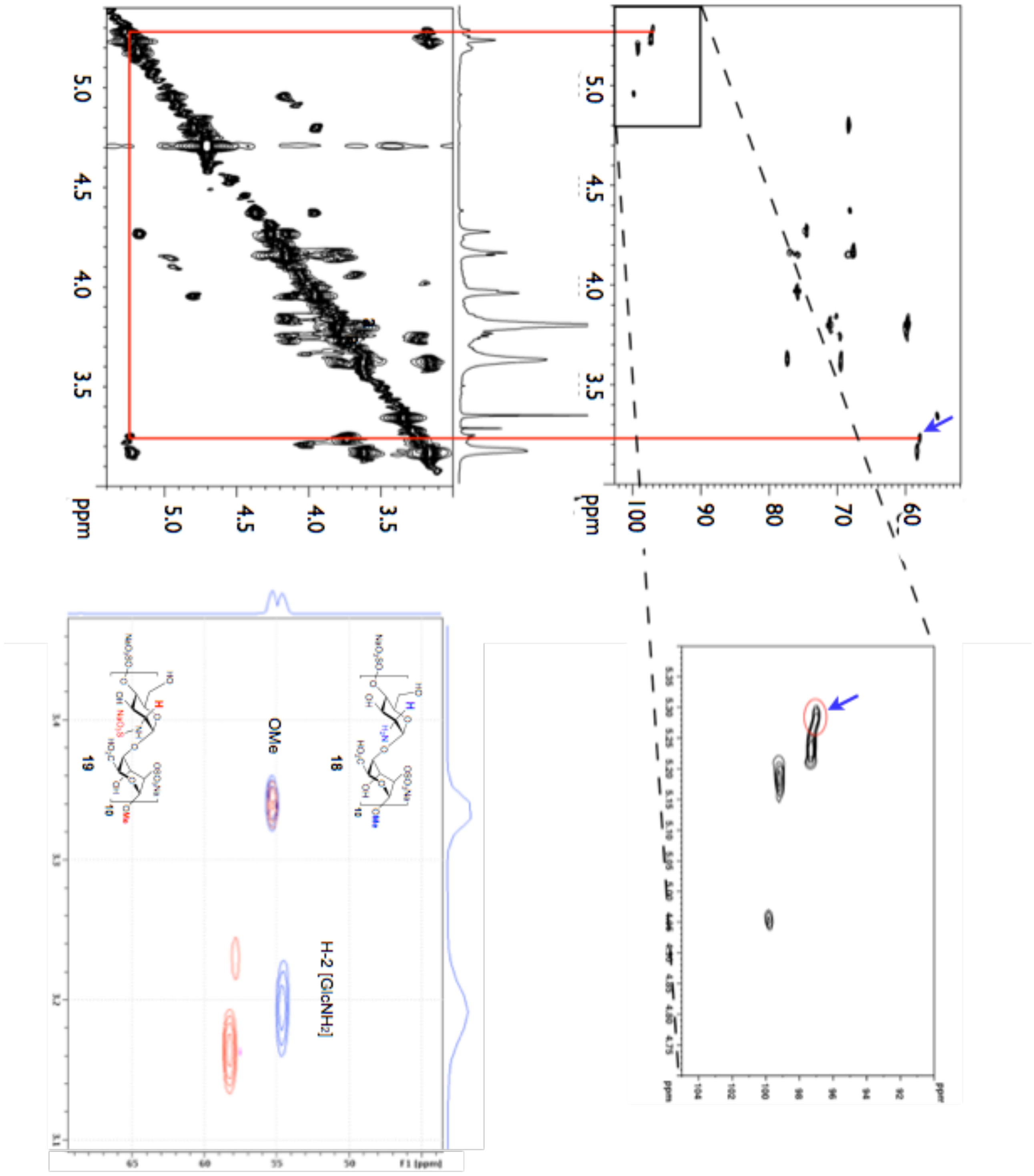

Supplementary Figure 71: HSQC NMR spectrum for **19**

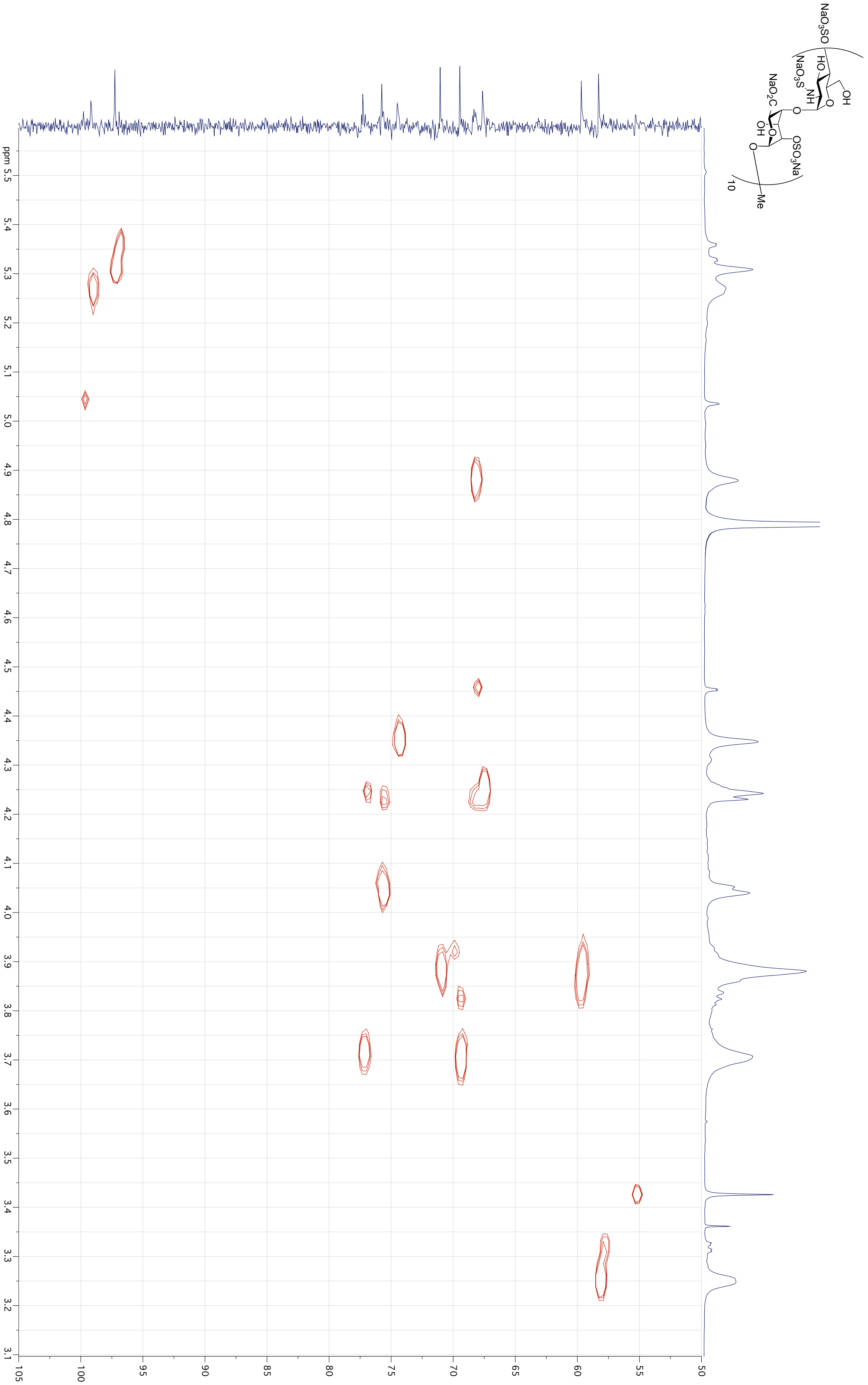

Supplementary Figure 72: HSQC (800 MHz, D_2O) spectra expansions for 18 (a) and 19 (b) to confirm ^{13}C chemical shift changes upon icosaccharide N-sulfation

a)

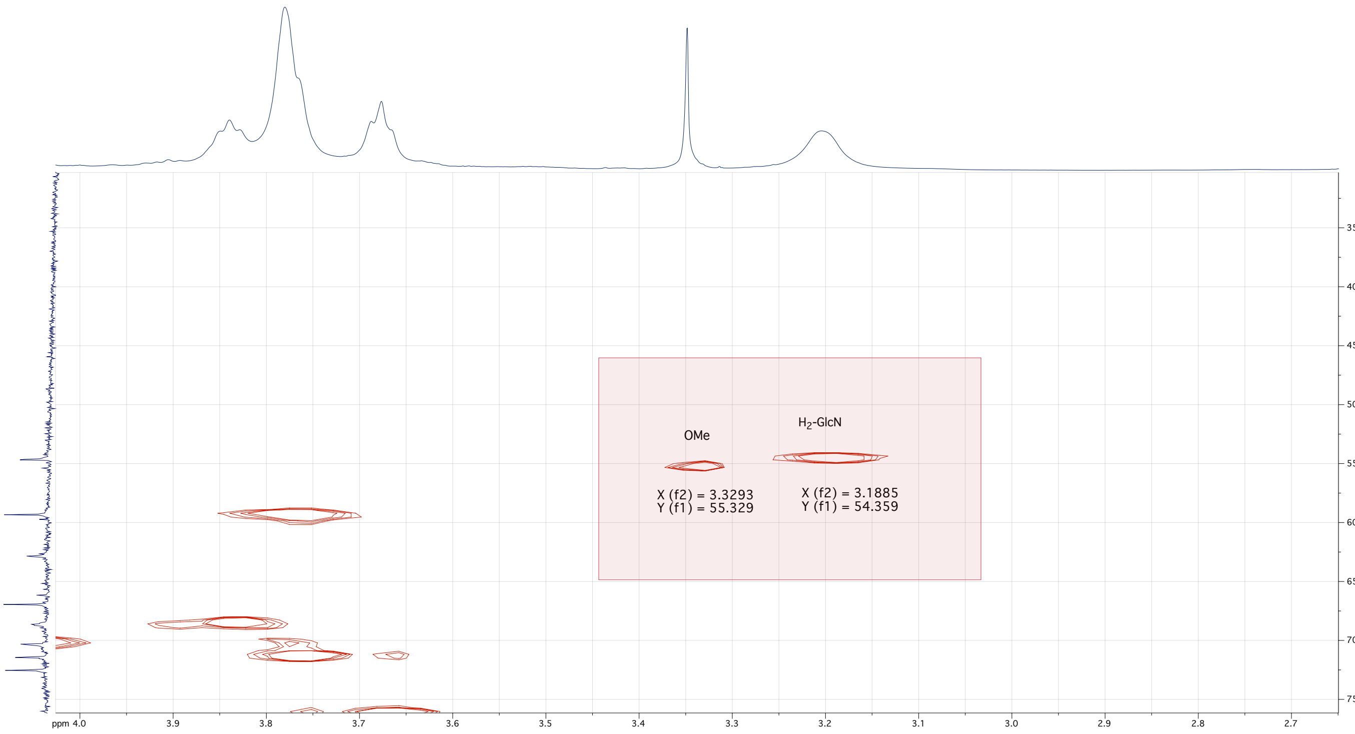

b)

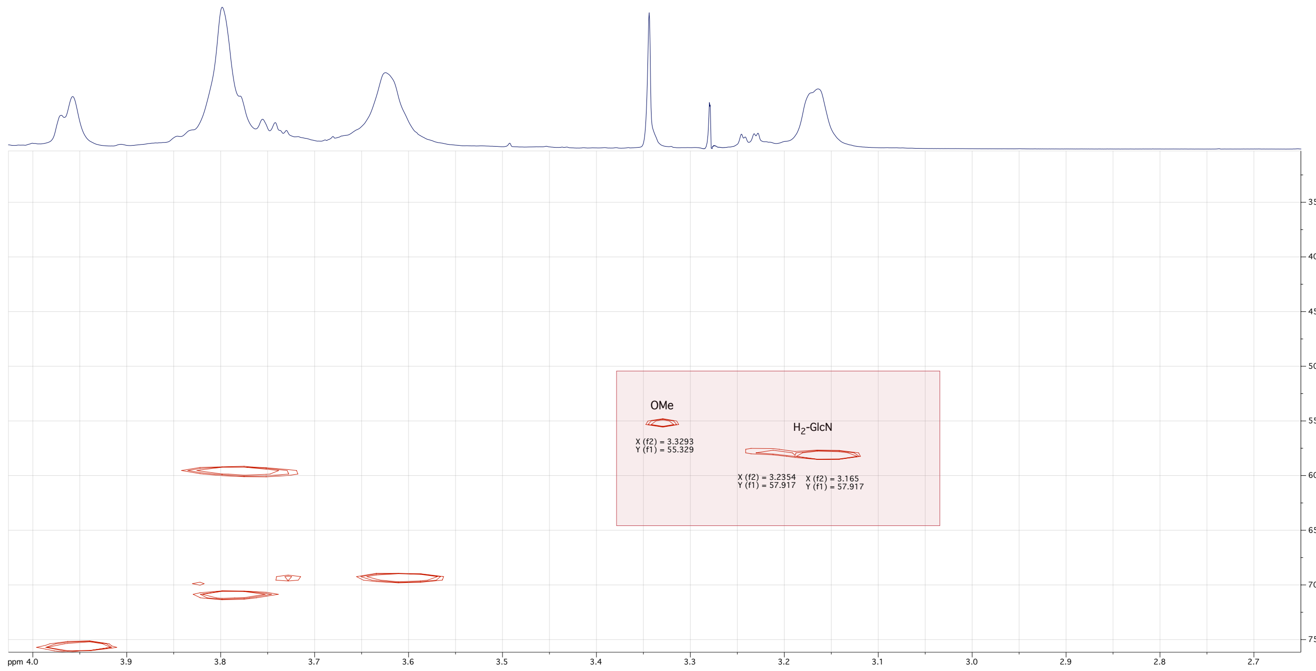

Supplementary Figure 73: PAGE analysis of 19

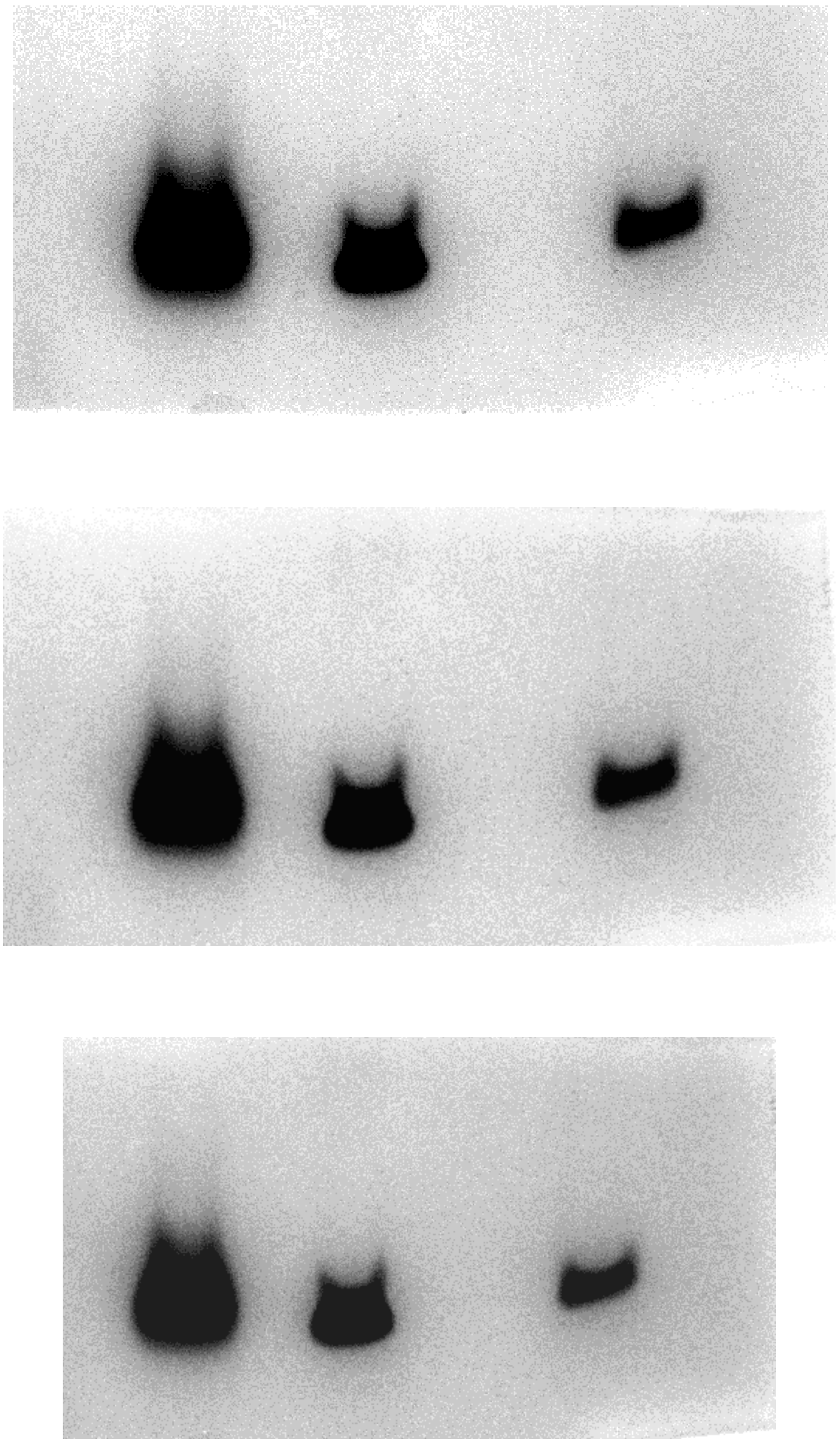

LHS is mixture of 18-22mers
middle and RHS are 20mer at
different concs..

Supplementary Figure 74: TLC system for differentiation and separation of long oligosaccharides

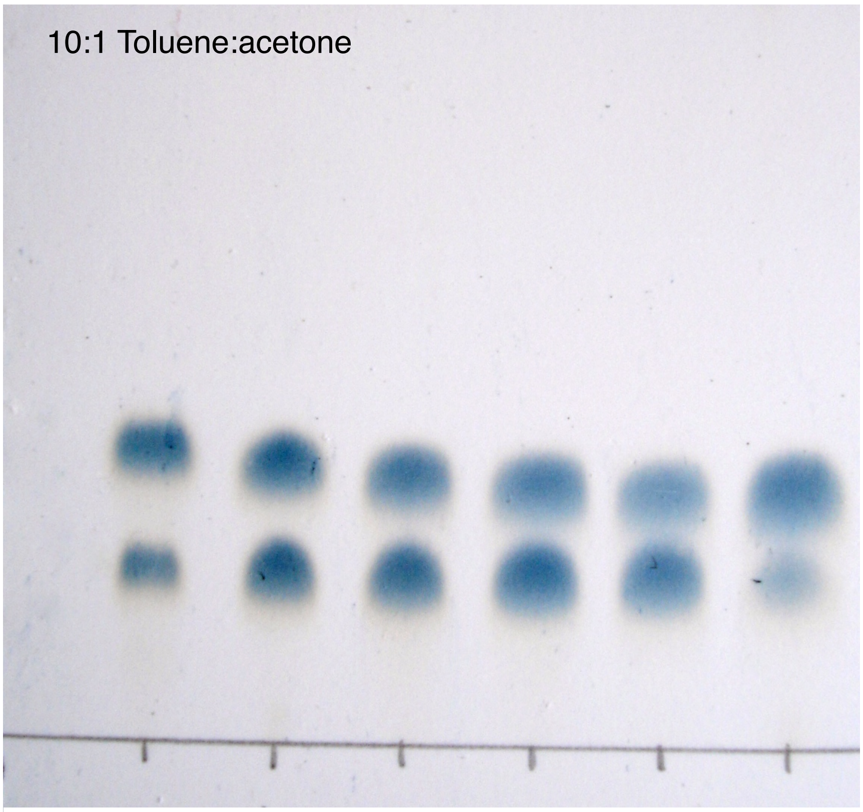

Product 4-O-TCA oligosaccharide

Acceptor substrate 4-OH oligosaccharide

4-TCA	16	20	24	28	32	36
4-OH	12	16	20	24	28	32
